# In Vitro and In Silico Evaluation of the Anti-Infective Potential of EF24-Analogous Curcuminoids: Antiprotozoal Activity and HIV-1 Ribonuclease H Inhibition

**DOI:** 10.3390/pathogens15080876

**Published:** 2026-08-20

**Authors:** Tariq A. Khan, Ibrahim S. Al Nasr, Waleed S. Koko, Kamal A. Qureshi, Nhat Quang Tu, Clémence Richetta, Federica Putzu, Laura Dettori, Olivier Delelis, Rainer Schobert, Angela Corona, Bernhard Biersack

**Affiliations:** 1 Department of Basic Health Sciences, College of Applied Medical Sciences, Qassim University, Buraydah 51452, Saudi Arabia; tara.khan@qu.edu.sa; 2 Department of Pharmacology and Toxicology, Institute for Nutrition and Translational Research in Metabolism (NUTRIM), Maastricht University, 6229 ER Maastricht, The Netherlands; 3 Department of Biology, College of Science, Qassim University, Buraydah 51452, Saudi Arabia; insar@qu.edu.sa (I.S.A.N.); wa.mohamed@qu.edu.sa (W.S.K.); 4 Department of Pharmaceutics, College of Pharmacy, Qassim University, Buraydah 51452, Saudi Arabia; ka.qurishe@qu.edu.sa; 5 Laboratoire de Biologie et Pharmacologie Appliquée (LBPA), ENS-Paris-Saclay, Centre National de la Recherche Scientifique (CNRS) UMR 8113, Université Paris-Saclay, 91190 Gif-sur-Yvette, France; tunhatquang2@gmail.com (N.Q.T.); clemence.richetta@ens-paris-saclay.fr (C.R.); delelis@lbpa.ens-cachan.fr (O.D.); 6 Laboratory of Molecular Virology, Department of Life and Environmental Sciences, University of Cagliari Biomedical Section, E Block, First Floor, Cittadella Universitaria di Monserrato SS554, 09042 Monserrato, CA, Italy; federica05putzu@libero.it (F.P.); laura.dettori@unica.it (L.D.); angela.corona@unica.it (A.C.); 7 Organic Chemistry Laboratory, University of Bayreuth, Universitätsstrasse 30, 95447 Bayreuth, Germany; rainer.schobert@uni-bayreuth.de

**Keywords:** curcuminoids, EF24 analogs, halogen, antiparasitic agents, leishmaniasis, toxoplasmosis, HIV, RNase H inhibitor, molecular docking

## Abstract

Curcumin derivatives (curcuminoids) exhibit considerable antiparasitic and antiviral activities. In this study, EF24-like bis-2-fluorobenzylidene piperidone derivatives were synthesized and evaluated for antiprotozoal activity and inhibition of the human immunodeficiency virus (HIV)-1 reverse transcriptase (RT)-associated RNase H (HIV-1 RNase H). A series of bis-arylidene piperidones and tetrahydro(thio)pyranones bearing halogen or nitro substituents were tested against *Leishmania major* (promastigotes and amastigotes) and *Toxoplasma gondii. L. major* promastigotes were the most sensitive parasitic model, with several compounds exhibiting low-nanomolar IC_50_ values, while *ortho*- or *para*-halogenated analogues demonstrated submicromolar activity against *T. gondii*. Among them, 3,4-dichlorophenyl (HPip-DC) and 4-bromophenyl (HPip-4Br) derivatives showed potent activity against *L. major* and HIV-1 RNase H. The entropy-uncorrected MM-GBSA effective binding energy estimates were −33.41, −9.29, −8.37, and −4.27 kcal/mol for the predicted *L. major* squalene monooxygenase–HPip-DC, bovine cytochrome bc_1_–DiFiD (2,4-difluorophenyl derivative), RNase H–HPip-4NO (4-nitrophenyl derivative), and RNase H–HPip-DC complexes, respectively. During 300 ns simulations, the ligands remained associated with their binding pockets. The Arg557 residue was crucial for HPip-4NO-mediated RNase H inhibition while the inhibitory activity of HPip-DC was much less dependent on this amino acid. These findings identify EF24-like curcuminoids with potent in vitro antiprotozoal activity and biochemical inhibition of HIV-1 RNase H. Antiviral activity was observed but confounded by host cell toxicity.

## 1. Introduction

Infectious diseases are a considerable global human health problem [1]. In particular, viral infections can emerge and spread quickly leading to pandemics threatening health and economic systems all over the world [2]. But parasitic diseases are also a global problem and require the development of new drugs [3]. In particular, the World Health Organization (WHO) list of neglected tropical diseases (NTDs) includes various viral, bacterial, fungal, protozoal, and worm infections where treatments are either unavailable or extremely toxic [4].

Leishmaniasis is currently listed as an NTD that is endemic in Latin America, South Europe, Africa, and Asia which manifests as cutaneous leishmaniasis (CL), mucocutaneous leishmaniasis (MCL), and visceral leishmaniasis (VL). Several kinetoplastid *Leishmania* species such as *L. major*, *L. tropica*, *L. mexicana*, *L. amazonensis*, etc., are the causative agents of CL leading to often stigmatizing skin lesions in up to one million, mostly young, patients every year [5,6]. CL patients are currently treated with pentavalent antimonials, miltefosine, amphotericin B or pentamidine [7]. In addition to the considerable toxicities of these drugs, a growing problem is the appearance of drug-resistant *Leishmania* forms [8]. Thus, new drugs against leishmaniasis are required to meet the clinical challenges of this NTD.

Apicomplexan parasites such as *Plasmodium* spp. and *Toxoplasma gondii* belong to the most perilous protozoal pathogens [9]. Toxoplasmosis can have severe outcomes in immunocompromised people and current therapy options include various antibiotics (sulfadiazine, pyrimethamine, trimethoprim, spiramycin, clindamycin) and the naphthoquinone atovaquone. The known adverse effects of these drugs limit their application and need to be addressed by the development of new therapies [10].

Globally, a big group of immunocompromised people in danger of acquiring lethal infectious diseases is HIV-positive. About 42 million people have died of the acquired immunodeficiency syndrome (AIDS) as a consequence of HIV infection since the 1980s. Worldwide, 39.9 million people including ca. 1.4 million children under 15 were infected in 2023 leading to 630.000 annual deaths, which are alarming numbers since HIV funding has also dropped by 7.9% between 2020 and 2023 [11]. Characteristic and perilous co-infections in HIV patients include fungal (*Pneumocystis jirovecii*, *Candida* and *Cryptococcus* spp.) and viral infections (cytomegalovirus), toxoplasmosis, and tuberculosis [12,13]. Moreover, co-infection with leishmaniasis is a health issue in tropical and sub-tropical regions and countries including South Europe, South Asia, sub-Saharan Africa, and Brazil [14]. Since there is still no cure of HIV infections available, the current therapies aim at a suppression of virus. For 30 years now, since the mid-1990s, the highly active antiretroviral therapy (HAART) based on a cocktail of drugs targeting HIV protease, HIV reverse transcriptase (RT), HIV integrase (IN), and viral entry is applied for the treatment of HIV, but its application is limited by adverse effects and emerging drug resistance [15]. Inhibition of the RNase H domain of RTs can become a suitable strategy to overcome resistance to classical RT inhibitors which target the DNA polymerase domain of RTs [16]. Several promising HIV-1 RNase H inhibitors have been disclosed, which coordinate the HIV-1 RNase H active-site Mg^2+^ ions in a competitive way or destabilize the protein as allosteric inhibitors [17]. Notably, some natural products also acted as inhibitors of HIV-1 RT-associated RNase H [18,19,20].

**Figure 1:**
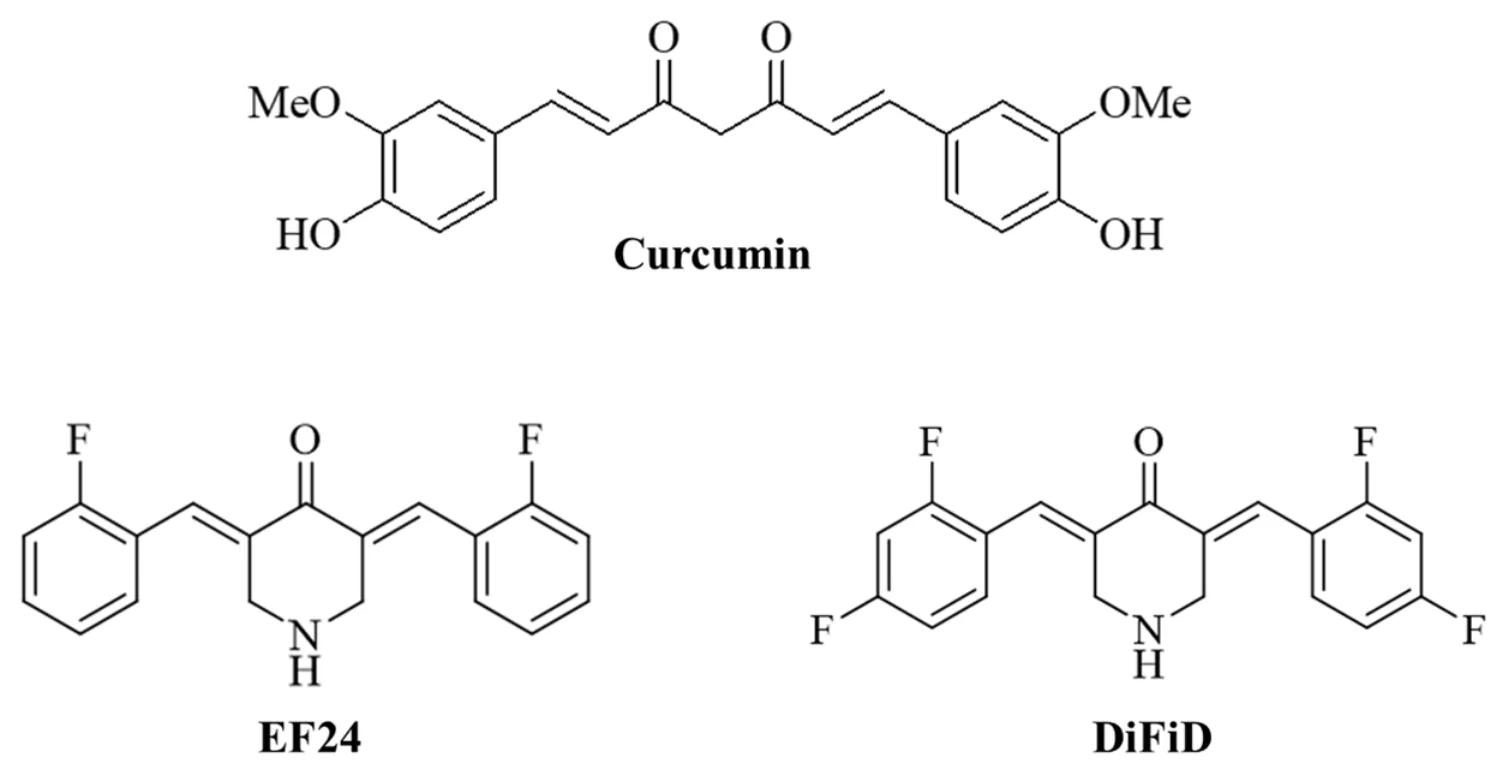
Chemical structures of the natural product curcumin and the synthetic anticancer active fluoro-substituted monoketone-based curcuminoids EF24 and DiFiD.

The natural phenol curcumin, 1,7-bis(4-hydroxy-3-methoxyphenyl)-1,6-heptadien-3,5-dione, exhibited promising anticancer activities and is a major ingredient of the root powder of turmeric (*Curcuma longa*), which is used as a spice in Indian cuisine and as a drug in Ayurveda [21,22]. Curcumin also showed potential as a drug against various tropical diseases [23]. It was active against protozoal *Leishmania*, *Plasmodium*, and *Toxoplasma* species [24]. Moreover, curcumin displayed antiviral properties including anti-HIV activities [25,26]. However, the medicinal application of natural curcumin is limited by its low bioavailability which might be improved by suitable formulations [27]. Aside of formulations, chemical modifications of the curcumin molecule including the reduction of the β-diketone to a monoketone moiety led to fluoroarylidene-piperidin-4-one curcuminoids such as EF24 with improved anticancer activities along with a higher bioavailability (Figure 1) [28,29]. The closely related curcuminoid DiFiD also displayed considerable anticancer activities against problematic pancreatic and head-and-neck cancers (Figure 1) [30,31]. In terms of infectious pathogens, monoketone-based curcuminoids displayed antibacterial, antifungal, and antiprotozoal activities [32,33,34,35,36]. Moreover, 3,4,5-trihydroxybenzylidene-based curcuminoids inhibited HIV-1 IN and viral replication [37]. Based on these studies and our previous works on curcuminoids, we investigated the antiparasitic and HIV-1 RT-associated RNase H-inhibitory activities of EF24, DiFiD, and their analogs using an integrated in vitro and in silico approach. In addition to EF24 and DiFiD, the study included 19 symmetric monocarbonyl curcuminoids based on central piperidin-4-one and tetrahydro(thio)pyran-4-one scaffolds, which are known from previous publications, and one new tetrahydrothiopyran-4-one analog (see Section 3.1) [38,39,40,41,42,43,44,45].

## 2. Materials and Methods

### 2.1. Curcuminoids

The syntheses of EF24 and DiFiD were described before [38]. The other 3,5-bis-(di/tri-)fluorobenzylidene piperidin-4-one and tetrahydro(thio)pyran-4-one curcuminoids, as well as the nitro- and chloro/bromobenzylidene derivatives (see Section 3.1) were also published before [38,39,40,41,42,43,44,45]. The preparation and analysis of the new compound Thio-DiFiD can be found in the Supplementary Materials File.

### 2.2. Toxoplasma gondii Cells Drug Testing Assay

This assay was done as mentioned previously [33]. Cells of the Vero cell line (ATCC^®^ CCL81™, Manassas, VA, USA) were used for the cultivation of *T. gondii* RH strain tachyzoites in 96-well plates with 5 × 10^3^ cells per well in 200 μL of RPMI 1640 medium (Invitrogen, Waltham, MA, USA). Cells were kept at 37 °C and 5% CO_2_ for 24 h, followed by the removal of media and washing with PBS. Next, RPMI 1640 medium with 2% FBS and parasites were added at a ratio of 5:1 parasite/cell. After incubation for 1 day, the cultures were subjected to test compounds at different concentrations (30, 10, 3.3, 1.1 and 0.37 μM). RPMI 1640 medium with 1% DMSO used as negative control while the experimental compounds and the positive control drug (atovaquone) were dissolved in 1% DMSO with RPMI 1640 medium. After 3 days incubation, the cells were washed with PBS, fixed in 10% formalin, and stained with 1% toluidine blue. An inverted photomicroscope was used to assess cell infection by determination of the infection index (number of infected cells out of 200 counted cells). The inhibition percentage was calculated as follows:

Inhibition (%) = [(I Control − I Experimental)/I Control] × 100

The effects of the compounds on parasite growth were expressed as IC_50_ values derived from three independent experiments [33].

### 2.3. Leishmania Major Drug Testing Assays

This assay was done according to our previously published report [33]. Promastigotes of *L. major* were obtained from an indoor patient at Ar Rass Public Hospital, Saudi Arabia (February 2016). The promastigotes were cryopreserved in liquid nitrogen at a concentration of 3 × 10^6^ parasites/mL. Serial passage of the parasite promastigotes was done by the injection of 1 × 10^6^ cells in the hind footpads of female BALB/c mice. Amastigote forms of the parasite were isolated from the mice after 8 weeks following the ethical guidelines of the Qassim University Research Ethics Committee (permission number 20-03-20).

*L. major* promastigotes were cultured in phenol red-free RPMI 1640 medium (Invitrogen, Waltham, MA, USA) with 10% FBS in 96-well plates at 10^6^ cells/mL (200 μL/well), and counted with a hemocytometer. Test compounds and positive control amphotericin B were added at final concentrations of 30, 10, 3.3, 1.1 and 0.37 μM. Negative controls contained 1% DMSO without test compounds. After incubation at 26 °C for 72 h, viable promastigotes were counted using the MTT colorimetric assay. Formed formazan was solubilized in detergent and the absorbance was measured at 570 nm using an ELISA reader (xMark, Bio-Rad, Hercules, CA, USA). IC_50_ values were determined from three independent experiments.

For the assessment of test compound activities against amastigotes in macrophages, peritoneal macrophages were harvested from 6 to 8-week-old BALB/c mice of different sex and plated at 5 × 10^4^ cells/well in 96-well plates containing phenol red-free RPMI 1640 medium with 10% FBS. After 4 h at 37 °C and 5% CO_2_ to promote adhesion, cells were washed with PBS, and 200 μL of an *L. major* promastigote solution (10 promastigotes per macrophage) was added. Plates were incubated for 24 h at 37 °C in a 5% CO_2_ environment to allow infection and amastigote differentiation. Infected cells were then washed with PBS and overlaid with RPMI 1640 medium containing the test compounds and positive control at the same concentrations as used for the promastigotes assay, followed by incubation at 37 °C and 5% CO_2_ for 72 h. Negative controls contained 1% DMSO only. The percentage of infected macrophages was determined microscopically after fixation, Giemsa staining, and removal of medium. IC_50_ values were calculated from three independent experiments [33].

### 2.4. Vero Cell and Macrophage Cytotoxicity

To assess the cytotoxicity of test compounds, the MTT assay was used. The Vero cells and macrophages (isolated from BALB/c mice as mentioned above in Section 2.3) were cultivated for 24 h at 37 °C in RPMI 1640 media with 10% FBS and 5% CO_2_ in 96-well plates (5 × 10^3^ cells/well/200 μL). Following a PBS washing step, the cells were treated with test compounds at different concentrations (30, 10, 3.3, 1.1 and 0.37 μM) in 10% FBS medium for 72 h. Negative controls consisted of cells that were only given media with 2% FBS and 1% DMSO. The cells were cultured for 4 h after the supernatant was disposed of and 50 μL of RPMI 1640 media containing 14 μL MTT (5 mg/mL) was added. After removing the supernatant once again, 150 μL of DMSO was added. IC_50_ values were determined from three independent experiments [33].

### 2.5. HIV1-RDDP-Independent-RNase H Inhibition

HIV-1 RT was expressed and purified as previously reported [16]. HIV-1 RT-associated RNase H inhibition assays were carried out according to a previously published method in a 384 multi-well format, in 30 µL reaction volume containing 50 mM Tris-HCl buffer (pH 7.8), 6 mM MgCl_2_, 1 mM dithiothreitol (DTT), 80 mM KCl, increasing concentrations of inhibitor, whose dilutions were made in DMSO, using 0.25 µM hybrid RNA/DNA 5′ GAUCUGAGCCUGGGAGCU-Fluorescin-3′5′-Dabcyl-AGCTCCCAGGCTCAGATC-3′ and 50 nM HIV-1 RT [46]. The compound RDS1759 was used as positive control of inhibition [47]. The reaction was monitored with a multilabel counter plate reader VictorNivo5 (PerkinElmer, Waltham, MA, USA) equipped with filters for 490/528 nm. To discard eventual false positive results. The samples were checked at time 0 to verify if the tested compounds were fluorescent in the reaction mix. Then the reaction was stopped at min 15 post activation. After blank subtraction and results normalization as percentage of positive DMSO controls, the IC_50_ values, i.e., the concentration able to inhibit 50% of enzymatic activity, was calculated by non-linear regression curve fit using Prisme v9.0 (log inhibitor vs. normalized response, variable slope). Results were reported as average and standard deviation of at least two independent experiments (each experiment performed at a different day) conducted in triplicate.

### 2.6. HIV Replication Assay

The HIV replication assay (CPRG assay) was carried out using cervix carcinoma-derived HeLa-P4 cells (10^4^ cells/well) [48] infected with HIV-1 (7.5 ng p24) and the viability of the cells was determined using the MTT assay as published previously [46].

### 2.7. Molecular Docking Studies

#### 2.7.1. Rationale for the Selection of Target Protein and Ligand

Molecular docking simulations were performed using the Molecular Operating Environment (MOE) software (version 2022.02; Chemical Computing Group, Montreal, Canada) [49]. The detailed docking methodology was adopted from our previous studies [50,51,52,53]. The ligands selected for docking were chosen based on their in vitro biological activity and selectivity profiles. For antitoxoplasmal analysis, DiFiD was selected because it exhibited the highest selectivity index among the tested curcuminoids against *T. gondii*. DiFiD and the reference drug atovaquone were screened against 19 *T. gondii*-associated proteins and the experimentally resolved bovine mitochondrial cytochrome *bc*_1_ complex (PDB ID: 6XVF). The bovine cytochrome *bc*_1_ complex was included as a mammalian structural reference to evaluate the accommodation of DiFiD within an experimentally resolved Complex III binding pocket. Its use should not be interpreted as direct evidence of DiFiD binding to the corresponding *T. gondii* orthologs. For the antileishmanial analysis, HPip-DC and the reference drug amphotericin B were screened against 19 putative *Leishmania*-associated protein targets. For the anti-HIV-1 analysis, HPip-DC and HPip-4NO were selected because both compounds exhibited potent inhibition of HIV-1 RT-associated RNase H activity in vitro. Both ligands, along with the reference RNase H inhibitor RDS1759, were docked against the HIV-1 RT-associated RNase H domain.

#### 2.7.2. Preparation of Target Protein and Ligand

The experimentally determined three-dimensional (3D) structures of the selected *T. gondii* and *Leishmania* target proteins, together with the HIV-1 reverse transcriptase-associated RNase H target, were retrieved from the Protein Data Bank (PDB), as specified in Tables S1–S3 [54]. For protein targets lacking suitable experimentally determined structures, homology models were generated using the SWISS-MODEL server, as listed in Table S2 [55].

In particular, the *L. major* squalene monooxygenase structure used for molecular docking and MD simulations was a computational homology model generated using SWISS-MODEL from the amino acid sequence of *L. major* strain Friedlin squalene monooxygenase-like protein (locus LMJF_13_1620; UniProt accession Q4QFZ2; NCBI RefSeq accession XP_001681906.1). Thus, the structure employed in this study was a predicted homology model rather than an experimentally determined X-ray crystallographic structure.

Before molecular docking, water molecules and other non-essential crystallographic components were removed, and co-crystallized ligands were temporarily retained to define binding sites. Protein structures were prepared using the QuickPrep module implemented in the Molecular Operating Environment (MOE), version 2022.02 [56]. During preparation, structural inconsistencies and missing atoms were corrected where applicable, hydrogen atoms were added, and protonation and tautomeric states were assigned using Protonate3D at pH 7.4 and 300 K [57]. The structures were subsequently energy-minimized using the Amber10 force field. Binding sites were defined based on the positions of co-crystallized ligands when available; otherwise, potential ligand-binding pockets were identified using the Site Finder module in the MOE [49].

The structures of DiFiD, HPip-DC, HPip-4NO, atovaquone (PubChem CID: 74989), amphotericin B (PubChem CID: 5280965), and RDS1759 (PubChem CID: 85470605) were converted to Structure Data File (SDF) format using OpenBabel [58]. The ligands were subsequently prepared in MOE 2022.02, with hydrogen atoms added and protonation states assigned using Protonate3D at pH 7.4 and 300 K. Energy minimization was performed using the Amber10 force field prior to molecular docking [49,57].

#### 2.7.3. Docking Protocol

Molecular docking simulations were performed using the MOE software (version 2022.02) following a standard induced-fit docking workflow adapted from previous studies [50,51,52,53]. Ligands were placed in the binding site using the Triangle Matcher placement method, an alpha triangle algorithm that generates ligand poses by aligning triplets of ligand atoms with triplets of receptor site points, achieving exhaustive and orientation-biased coverage of the binding pockets. The initial poses were ranked using the London *dG* scoring function, an empirical force-field-based function that estimates the free energy of binding in the gas phase by decomposing the ligand–receptor interaction into contributions from hydrogen bonding, metal–ligand interactions, hydrophobic contacts, and entropic terms [50,51,52,53]. For each compound, 30 docking poses were generated, and the top five best-ranked conformations were refined using the Induced Fit protocol and subsequently rescored using the Generalized Born Volume Integral/Weighted Surface Area (GBVI/WSA) *dG* scoring function, which accounts for solvation effects and receptor flexibility. For each protein–ligand pair, the pose with the most favorable docking score that retained chemically plausible protein–ligand contacts was selected for subsequent interaction analysis and MD simulations [50,51,52,53]. The reference ligands, atovaquone, amphotericin B, and RDS1759, were included as comparative controls for the *Toxoplasma*, *Leishmania*, and HIV-1 RNase H systems, respectively, to provide a baseline for comparing the predicted docking scores and interaction patterns. Their inclusion did not consider the formal validation of docking accuracy [50,51,52,53]. No formal crystallographic re-docking with RMSD-based pose-recovery assessment, broader docking-accuracy benchmarking, or consensus-scoring validation was performed for the present target panel. Therefore, the docking results were interpreted as comparative and hypothesis-generating predictions rather than mechanistic evidence of target engagement.

### 2.8. MD Simulations and MM-GBSA Binding Energy Analysis

Four representative protein–ligand complexes were selected for MD simulation based on their docking scores, in vitro activities, and binding-site interactions: the DiFiD complex with the experimentally resolved bovine mitochondrial cytochrome *bc*_1_ structural surrogate/reference (PDB ID: 6XVF), the *Leishmania* squalene monooxygenase model–HPip-DC complex, and the HIV-1 RT-associated RNase H (PDB ID: 3K2P) complexes with HPip-DC and HPip-4NO [50,52]. MD simulations were performed using the GROMACS software 2024.2 [56]. The protein parameters were assigned using the CHARMM36m force field [59], and the ligand parameters were generated using the CHARMM General Force Field [60]. The procedure was adapted from our previous study [50,52]. Each protein–ligand complex was placed in a truncated octahedral box, solvated with TIP3P water, and neutralized with ions to a final KCl concentration of 0.15 M to approximate physiological ionic strength. Energy minimization was performed using the steepest-descent algorithm for up to 50,000 steps or until the maximum force dropped below 1000 kJ mol^−1^ nm^−1^ [50,52]. After minimization, each system underwent a two-step equilibration procedure comprising 1000 ps under constant volume and temperature (NVT) conditions, followed by 1000 ps under constant pressure and temperature (NPT) conditions for a total equilibration time of 2 ns. The temperature was maintained at 310.15 K using a velocity-rescaling thermostat, and the pressure was controlled using a Parrinello–Rahman barostat. System equilibration was assessed based on the stabilization of the backbone RMSD, temperature, pressure, and density prior to the production run. All covalent bonds involving hydrogen atoms were constrained using the LINCS algorithm, allowing a 2 fs integration time step. Production simulations were performed for 300 ns under periodic boundary conditions in all directions. Long-range electrostatic interactions were treated using the Particle Mesh Ewald (PME) method, and a cutoff of 1.2 nm was applied to short-range electrostatic and van der Waals interactions. The coordinates were saved every 10 ps for each trajectory. Trajectory analysis was performed using standard GROMACS tools, and the final graphics were refined using custom Python (version 3.13.9) scripts for publication-quality presentations. Post-simulation analyses were performed to evaluate the structural stability, residue-level flexibility, and overall compactness of the protein complexes. Global stability was monitored by RMSD, ligand retention by ligand RMSD, local flexibility by root-mean-square fluctuation (RMSF), and structural compactness by the radius of gyration (RoG). The solvent-accessible surface area (SASA) was calculated to monitor changes in protein surface exposure during the simulations [50,52]. Protein–ligand interactions were further characterized by hydrogen bond and hydrophobic contact analyses and time-resolved distance measurements of selected residue–ligand atom pairs. These analyses were used to evaluate the persistence and fluctuation of key contacts within the binding pocket and to describe ligand accommodation over time [50,52]. The temporal behavior of these structural descriptors was used to assess the trajectory stability over the simulated 300 ns timescale; however, no formal statistical or thermodynamic convergence analysis was performed. Each selected protein–ligand complex was represented by a single, independently initiated 300 ns production trajectory; therefore, reproducibility across replicate simulations was not evaluated. The binding free energies were estimated using the Molecular Mechanics Generalized Born Surface Area (MM-GBSA) approach implemented in the gmx MMPBSA package [61]. For this purpose, a 100 ns segment of the trajectory corresponding to 200–300 ns was selected from which 200 evenly distributed, backbone-aligned frames were extracted after removing the translational and rotational motions. The calculated energy terms included van der Waals (E_vdW_), electrostatic (E_ele_), polar solvation (G_polar_), and nonpolar solvation (G_nonpolar_) energies. The total binding free energy (ΔG_binding_) was determined using the following equation:

*ΔG_binding_* = *E_vdW_* + *E_ele_* + *G_polar_* + *G_nonpolar_*

### 2.9. Protein–Ligand Distance Analysis

Protein–ligand distance analysis was performed using the production MD trajectories of each of the complexes. The final trajectory frame was extracted as end.pdb and used to identify representative polar and hydrophobic atom pairs between the protein and ligand. Polar pairs consisted of protein and ligand nitrogen or oxygen atoms, whereas hydrophobic pairs consisted mainly of carbon atoms. The distances were calculated throughout the simulation using the GROMACS gmx distance tool. The resulting .xvg files were converted to .dat format and plotted against the simulation time. Polar contacts with distances of ≤0.35 nm were considered hydrogen-bond-like interactions, although no angular criterion was applied, and hydrophobic contacts were evaluated primarily within the 0.40–0.45 nm range. The automated workflow used end.pdb for atom pair selection and generated individual distance plots for each selected interaction.

## 3. Results

### 3.1. Curcuminoid Test Compound Series

**Figure 2:**
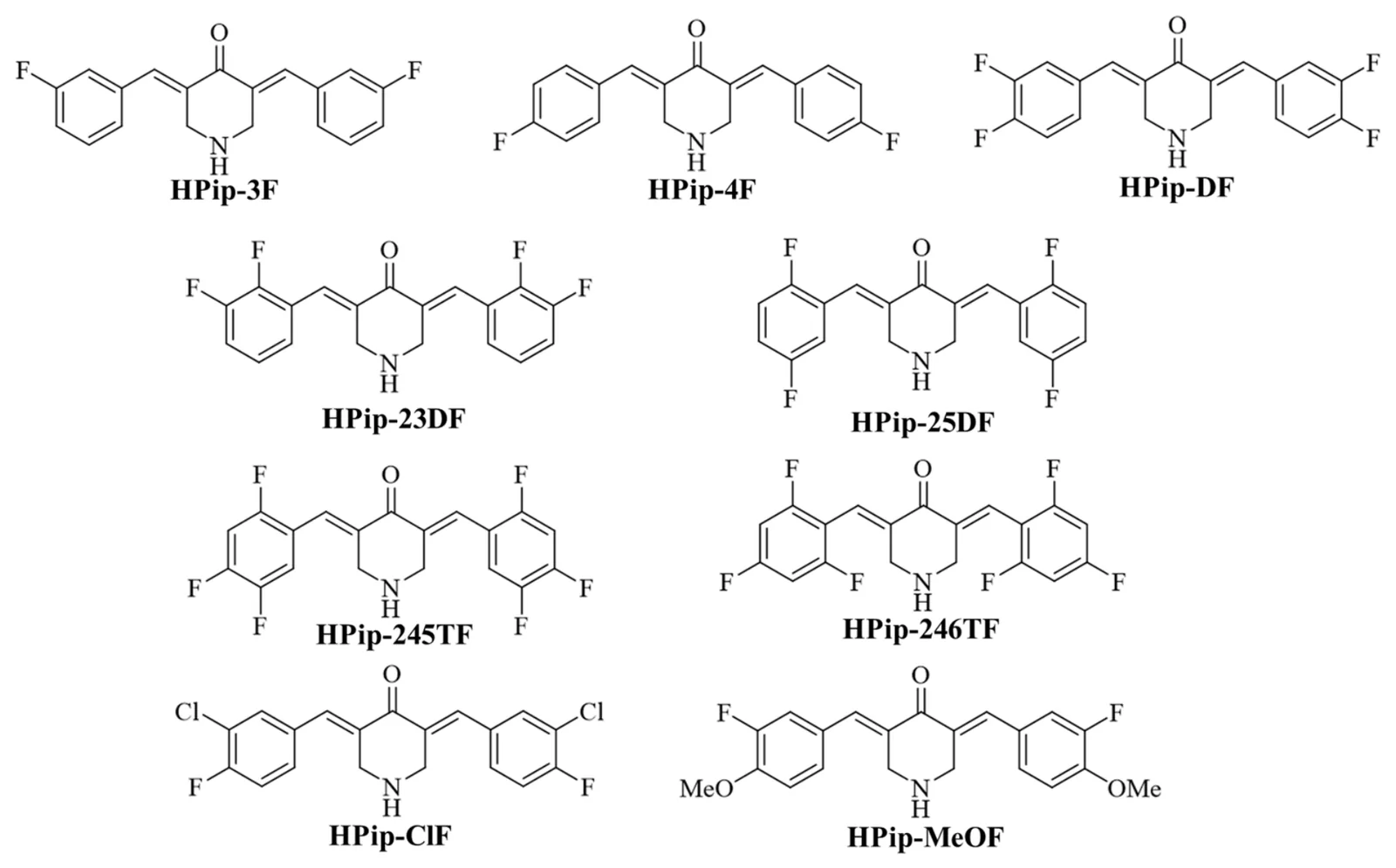
Chemical structures of synthetic fluoro-substituted piperidin-4-one-based curcuminoids.

**Figure 3:**
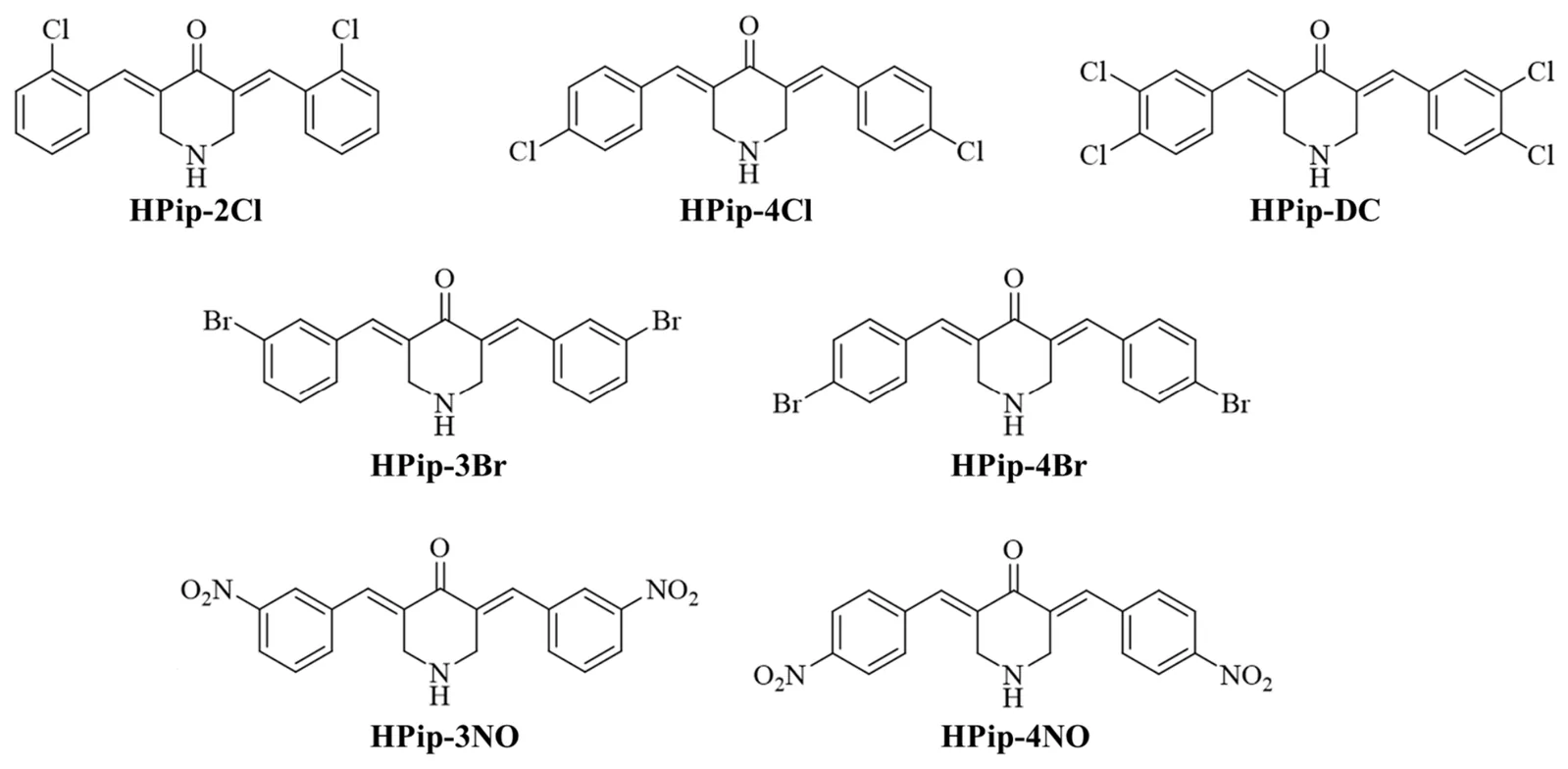
Chemical structures of synthetic chloro-/bromo-/nitro-substituted piperidin-4-one-based curcuminoids.

**Figure 4:**
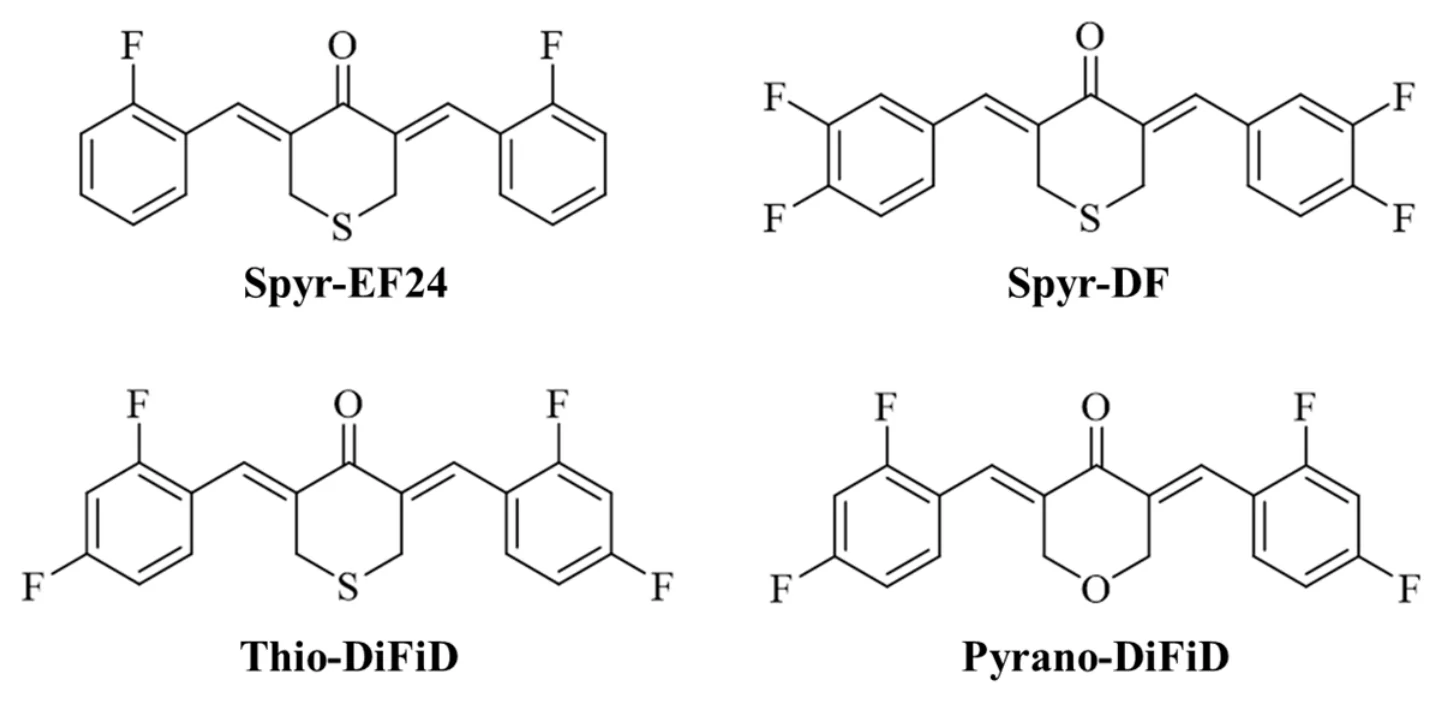
Chemical structures of synthetic fluoro-substituted tetrahydro-thiopyran-4-one-based curcuminoids and a tetrahydropyran-4-one analog of DiFiD.

The structures of the symmetric EF24- and DiFiD-related curcuminoids with identically substituted benzylidene moieties, which were used in this study, are presented in Figure 2, Figure 3 and Figure 4. Together with EF24 and DiFiD, the investigated compound series comprises 19 known bis-halophenyl- or bis-nitrophenylpiperidin-4-one derivatives labeled as ’’HPip’’ compounds (Figure 2 and Figure 3). In addition, three fluoro-substituted tetrahydrothiopyran-4-ones, among them the new compound Thio-DiFiD, and one tetrahydropyran-4-one analog of DiFiD (Pyrano-DiFiD) were evaluated (Figure 4).

### 3.2. Antiparasitic Activity Against Toxoplasma gondii

DiFiD was initially selected as a member of the EF24-related curcuminoid compound class for antiparasitic testing in *T. gondii* parasites. Since it revealed high antitoxoplasmal activity (IC_50_ = 1.2 µM) and certain selectivity, EF24 and other structurally related curcuminoids were also tested (Table 1). The approved antitoxoplasmal drug atovaquone was used as a positive control. EF24, HPip-4F, HPip-2Cl, HPip-4Cl and Spyr-DF were the most active compounds against *T. gondii* with IC_50_ values below 1 µM. HPip-2Cl performed best (IC_50_ = 0.6 µM) closely followed by HPip-4Cl, Spyr-DF (both IC_50_ = 0.7 µM), and EF24 (IC_50_ = 0.8 µM). However, their selectivity is only low and they were also toxic to Vero kidney cells. The highest selectivity among the tested curcuminoids was determined for DiFiD and it was three-times more active against *T. gondii* than against Vero cells (selectivity index/SI = 3.1). HPip-MeOF was the least active compound against *T. gondii* with an IC_50_ value of 10.5 µM.

Structure–activity relationships of the piperidin-4-ones showed that mono-fluoro and mono-chloro substituents at *ortho* or *para* positions are superior to bromo and nitro substituents and di-/trifluoro/chlorophenyl derivatives. In contrast, the 3,4-difluorophenyl moiety performed best among the (thio)pyran-4-ones while the analogous 2-fluorophenyl and 2,4-difluorophenyl derivatives Spyr-EF24, Thio-DiFiD, and Pyrano-DiFiD were less active than their parent compounds EF24 and DiFiD.

Dose–response curves of the antitoxoplasmal activity assays and the associated toxicity experiments with Vero cells can be found in the Supplementary Materials (Figures S25 and S26).

### 3.3. Antiparasitic Activity Against Leishmania major

The antileishmanial activity of EF24, DiFiD, and their analogs was tested using *L. major* promastigotes and amastigotes (Table 2). Dose–response curves of the antileishmanial activity assays and the associated toxicity experiments with murine macrophages can be found in the Supplementary Materials (Figures S27–S29). The promastigotes turned out to be highly sensitive to the curcuminoids. HPip-DC, HPip-3Br and HPip-4Br were the most active compounds (IC_50_ < 0.1 µM) and more active than the reference drug amphotericin B. Among the fluoro-substituted derivatives, HPip-4F, HPip-245TF and Spyr-DF were most active (IC_50_ = 0.10–0.11 µM). HPip-246TF was the least active derivative (IC_50_ = 10.8 µM). It is noteworthy that HPip-MeOF, the least antitoxoplasmal compound, showed high antileishmanial activity against promastigotes.

The activities of the curcuminoids against amastigotes were generally lower. Spyr-DF was the most active compound against the amastigotes (IC_50_ = 1.9 µM) but still less active than amphotericin B. HPip-DF, HPip-DC, and HPip-4Br were further analogs with distinct activities against amastigotes and IC_50_ values of 5–7 µM. The least active compounds against amastigotes were HPip-3F, HPip-23DF, and HPip-4NO with IC_50_ values above 30 µM.

Several fluorinated piperidin-4-ones and the 4-nitrophenyl derivative HPip-4NO were also tested for toxicity to murine macrophages (Table 2). Notably, the tested curcuminoids were less toxic to macrophages than amphotericin B. Considerable selectivities for *L. major* promastigotes were obtained with SI values above 100 for HPip-245TF and HPip-MeOF, and SI values above 70 for HPip-ClF and HPip-4NO. Obviously, based on their lower activities against amastigotes, the selectivities of the selected curcuminoids for *L. major* amastigotes were much lower.

Because of technical issues we were not able to gain enough murine macrophages to determine the toxicities of all curcuminoids. Thus, the selectivities of the curcuminoids for promastigotes when compared with their toxicities to Vero kidney cells were also investigated (Table 2). Notably, considerable selectivities in terms of the kidney cells were also achieved (SI values of up to 40 and 60 for HPip-3Br and HPip-DC, respectively). But where a comparison with macrophage toxicity was possible, the macrophages appeared to be more tolerant to curcuminoid treatment than the kidney cells.

In terms of structure–activity relationships, bromo- and chloro-substituents led to the highest activities against promastigotes. Among the fluorinated compounds, the 4-fluorophenyl derivative HPip-4F was as active as the 4-chlorophenyl analog HPip-4Cl and more active than other mono-fluorophenyl analogs EF24, Spyr-EF24, and HPip-3F. This suggests a preference for *para*-substituents over *meta*- and *ortho*-substituents. The 2,4,5-trifluorophenyl derivative HPip-245TF was as active as HPip-4F but 100-times more active than its isomer, the 2,4,6-trifluorophenyl analog HPip-246TF. The 3,4-difluorophenyl Spyr-DF was more active than HPip-DF and the other difluorophenyl derivatives (up to 10-times more active than the tetrahydro(thio)pyrano analogs Thio-DiFiD and Pyrano-DiFiD). Notably, the close 2-fluorophenyl analogs EF24 and Spyr-EF24 did not differ much in their activity against promastigotes and amastigotes. Amastigotes were most sensitive to 3,4-difluoro- and 3,4-dichlorophenyl compounds (Spyr-DF, HPip-DF and HPip-DC). 3-Fluoro substituents as in HPip-3F and HPip-23DF appeared to be less beneficial in the amastigotes. Notably, the 3-bromophenyl HPip-3Br was also less active than the analogous 4-bromophenyl HPip-4Br. However, the 3-nitrophenyl HPip-3NO was more active against amastigotes than the 4-nitrophenyl HPip-4NO. Pyrano-DiFiD was less active than DiFiD against promastigotes, but both compounds exhibited the same activity against amastigotes. Both DiFiD and Pyrano-DiFiD were distinctly more active against amastigotes than Thio-DiFiD, indicating a negative effect of the sulfur on the activity of DiFiD compounds.

### 3.4. Inhibition of HIV-1 RT-Associated RNase H

In our quest for the identification of new inhibitors of viral enzymes such as HIV-1 RT-associated RNase H, DiFiD was part of an initial screening of representative compounds of various compound classes and revealed a distinct HIV-1 RNase H inhibition (IC_50_ = 5.37 µM). Thus, EF24 and the other curcuminoids used in the antiparasitic activity assays were also evaluated for their HIV-1 RNase H-inhibitory activities (Table 3, Figure S30). In order to exclude possible interference in the fluorescence readout, the fluorescence or quenching effect of compounds was assessed in the mix prior reaction activation (at t = 0) at the highest concentration tested (100 µM), and no interference was detected (Figure S31). HPip-DC and HPip-4Br were the most active HIV-1 RNase H inhibitors (IC_50_ < 2 µM), closely followed by HPip-4NO, HPip-3NO, Pyrano-DiFiD, and HPip-3Br (IC_50_ < 3 µM). These compounds were also more active than the positive control RDS1759. Among the fluoro-substituted piperidin-4-ones, HPip-ClF, HPip-DF, DiFiD, and HPip-4F were the most active compounds (IC_50_ < 6 µM) with activities in the range of RDS1759. Notably, EF24 (IC_50_ >100 µM) and HPip-2Cl (IC_50_ > 30 µM) were inactive. Further weak inhibitors were HPip-3F and HPip-25DF (both IC_50_ > 20 µM).

The most active compound and 3,4-dichlorophenyl derivative HPip-DC and the 3-chloro-4-fluorophenyl HPip-ClF were more active than the 4-chlorophenyl and 2-chlorophenyl analogs (the latter was inactive) and all fluoro-substituted piperidin-4-ones. The 4-fluorophenyl and 4-bromophenyl derivatives HPip-4F and HPip-4Br were more active than their 2-flourophenyl and 3-bromo/fluoro-substituted analogs, indicating an important role for *para*-substituents. These activity differences were more pronounced in the mono-fluoro- (HPip-4f > HPip-3F > EF24) and mono-chloro-substituted piperidin-4-ones (HPip-4Cl > HPip-2Cl) than in the mono-bromo analogs (HPip-4Br was only slightly more active than HPip-3Br). Indeed, both nitrophenyls HPip-3NO and HPip-4NO showed virtually the same RNase H inhibition. Among the difluorophenyl-based piperidin-4-ones, the 2,4-difluoro- (DiFiD) and 3,4-difluorophenyl (HPip-DF) derivatives were superior to the 2,3-difluoro- and 2,5-difluorophenyl derivatives (Hpip-23DF and HPip-25DF). Again, the 2,4,5-trifluorophenyl compound HPip-245TF was more active than the 2,4,6-trifluoro-isomer HPip-246TF. While the 2-fluorophenyl derivative EF24 was inactive, replacement of the piperidin-4-one NH-group by sulfur led to considerable activity by the EF24 analog Spyr-EF24. In contrast, the 3,4- and 3,5-difluorophenyl tetrahydro-thiopyranones Spyr-DF and Thio-DiFiD were less active than the piperidin-4-ones DiFiD and HPip-DF, while the promising activity of the tetrahydropyrano compound Pyrano-DiFiD indicates a beneficial effect of the pyrano-oxygen in comparison to NH and sulfur, which might be further exploited in future studies.

**Figure 5:**
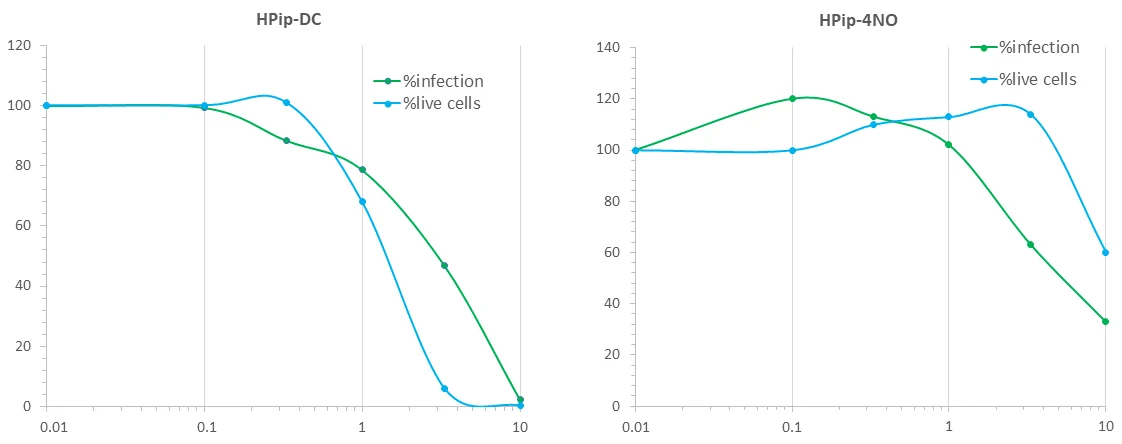
Inhibition of HIV-1 replication (green line/dots, % infection) and toxicity to HeLa-P4 host cells (blue line/dots, % live cells) by the HIV-1 RNase H inhibitors HPip-DC and HPip-4NO (0.1, 0.3, 1.0, 3.3, and 10 µM). *x*-axis, drug concentration (µM); *y*-axis, % infection or % live cells.

Based on their promising HIV-1 RNase H-inhibitory activities the compounds HPip-DC and HPip-4NO were selected and tested for HIV-1 replication inhibition in HeLa-P4 cells by using the well-established CPRG and MTT methods, respectively (Figure 5). Compound HPip-DC was found to be too toxic to the host cells and inhibition of viral replication was accompanied by cytotoxic activity. In contrast to that, HPip-4NO exhibited antiviral activity (approx. 40% inhibition) at the highest non-toxic concentration of 3.3 µM. At higher concentration (10 µM), HPip-4NO showed stronger viral replication suppression (approx. 60% inhibition) but also considerable cytotoxicity (approx. 40% toxicity). How far using a different host cell line (instead of the cervix carcinoma-derived HeLa-P4 cells) might lead to improved selectivity of both compounds remains to be shown.

### 3.5. Molecular Docking Studies and Target Prioritization

**Figure 6:**
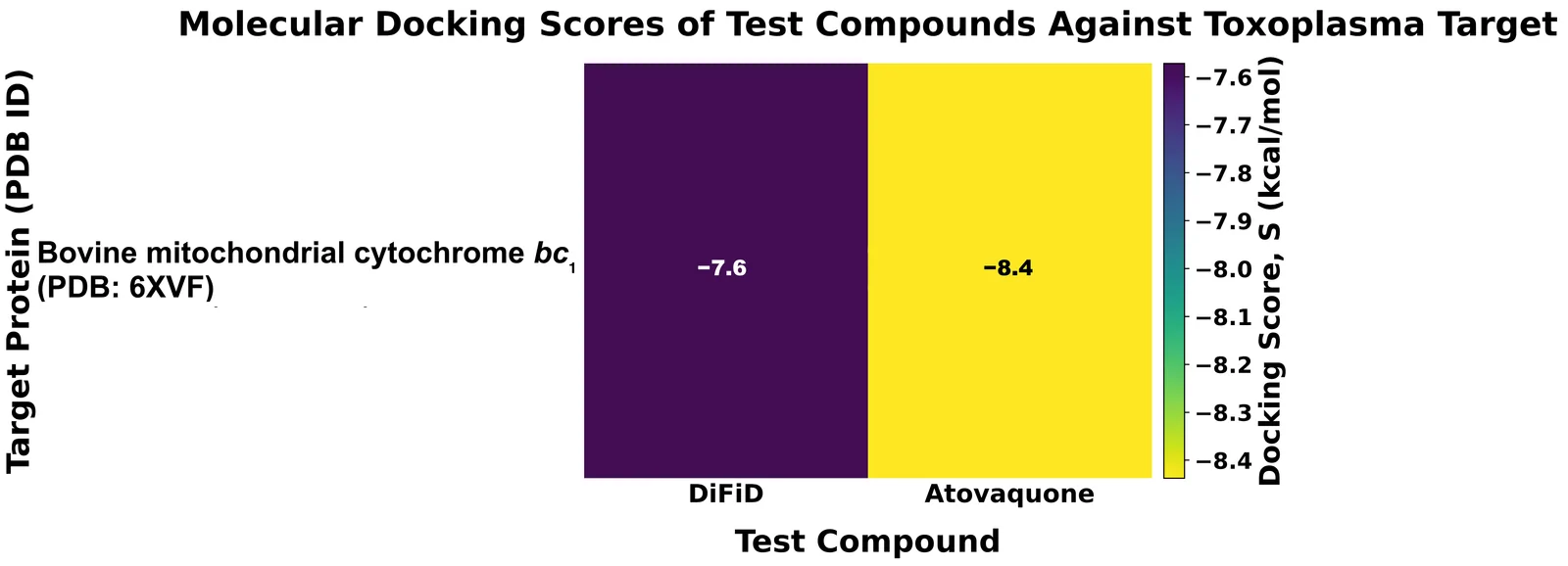
Molecular docking scores of DiFiD and atovaquone against bovine mitochondrial cytochrome *bc*_1_ (PDB ID: 6XVF), which was used as a mammalian structural reference. The heat map compares the predicted docking scores of DiFiD with those of the reference drug, atovaquone. More negative values indicate more favorable predicted docking scores within the applied docking protocol and should not be interpreted as experimental binding affinity.

**Figure 7:**
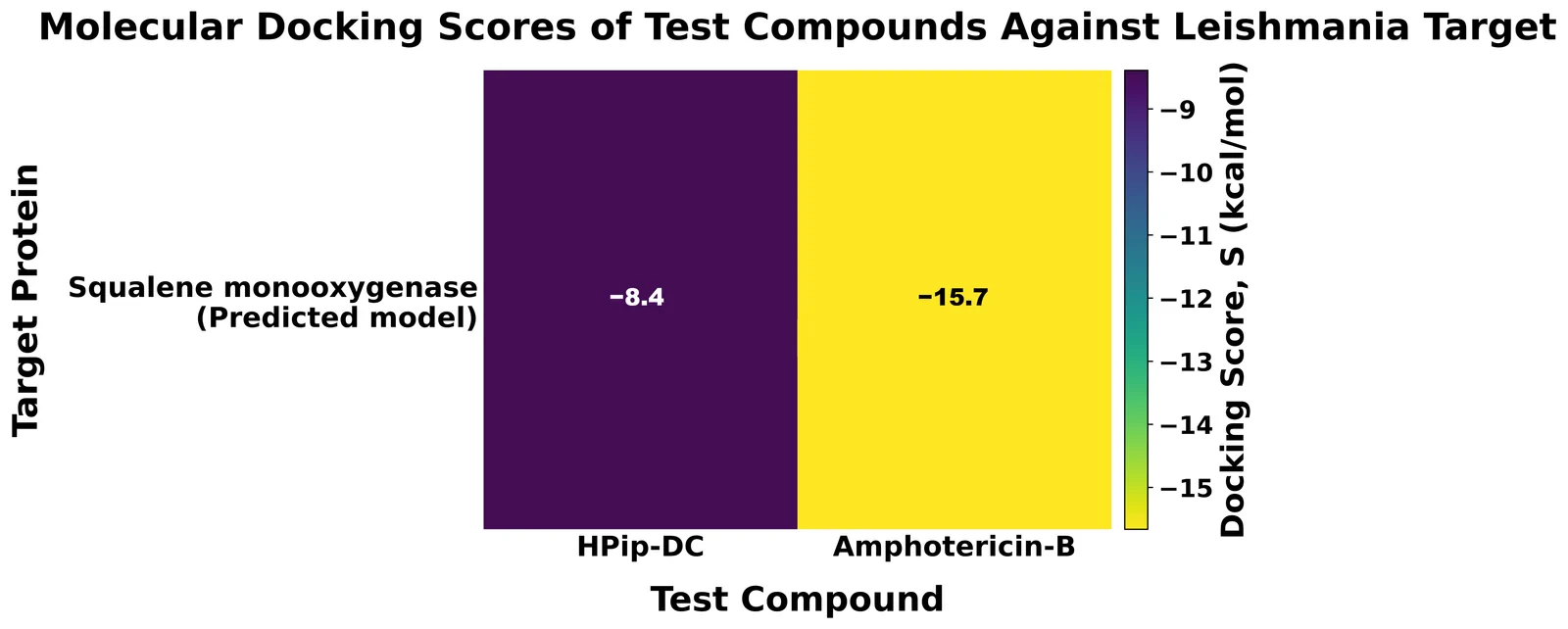
Molecular docking scores of HPip-DC and amphotericin B against the predicted *L. major* squalene monooxygenase model. The heat map compares the predicted docking scores of HPip-DC and the reference drug amphotericin B. More negative values indicate more favorable predicted docking scores within the applied docking protocol and should not be interpreted as experimental binding affinity.

**Figure 8:**
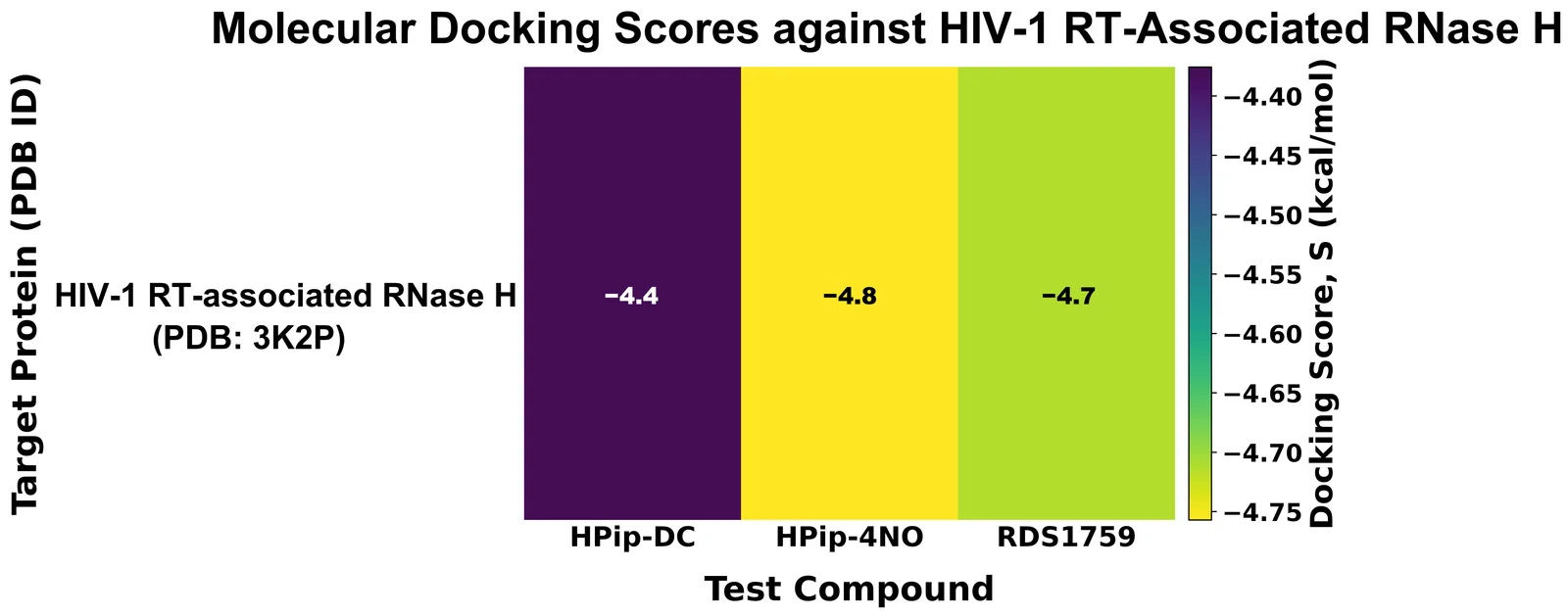
Molecular docking scores of HPip-DC, HPip-4NO, and RDS1759 against HIV-1 RT-associated RNase H (PDB ID: 3K2P). The heat map compares the predicted docking scores of HPip-DC, HPip-4NO, and the reference RNase H inhibitor, RDS1759. More negative values indicate more favorable predicted docking scores within the applied docking protocol and should not be interpreted as experimental binding affinity.

Molecular docking was performed to identify potential targets associated with the antitoxoplasmal, antileishmanial, and HIV-1 RT-associated RNase H-inhibitory activities of the selected compounds. DiFiD was screened against several *T. gondii*-associated proteins and bovine mitochondrial cytochrome bc_1_ (PDB ID: 6XVF), which was used as a mammalian-structure reference (Figure S1). The most favorable docking scores were obtained for bovine mitochondrial cytochrome bc_1_ (−7.6 kcal/mol), ROP5C (−7.5 kcal/mol), TgProRS (−7.4 kcal/mol), and TgCDPK1 (−7.1 kcal/mol). Atovaquone showed a stronger predicted interaction with mitochondrial cytochrome bc_1_ than DiFiD, with docking scores of −8.4 and −7.6 kcal/mol, respectively (Figure 6). The predicted binding orientations and surrounding residues of DiFiD and atovaquone are shown in Figures S3–S6, respectively. HPip-DC was docked onto a panel of *Leishmania*-associated proteins (Figure S2). The most favorable score was obtained for squalene monooxygenase (−8.4 kcal/mol), followed by N-myristoyltransferase (−7.5 kcal/mol), deoxyhypusine hydroxylase (−7.3 kcal/mol), squalene synthase (−7.2 kcal/mol), and GPI mannosyltransferase 1 (−7.2 kcal/mol). Amphotericin B showed a more negative docking score than HPip-DC against the predicted squalene monooxygenase model (−15.7 vs. −8.4 kcal/mol; Figure 7). The corresponding 2D interaction maps and 3D binding poses are shown in Figures S7–S10. However, this comparison should be interpreted cautiously because amphotericin B is substantially larger, and its established mechanism of action primarily involves membrane sterol binding. Docking against HIV-1 RT-associated RNase H produced scores of −4.8, −4.7, and −4.4 kcal/mol for HPip-4NO, RDS1759, and HPip-DC, respectively (Figure 8). HPip-4NO therefore showed a predicted interaction comparable to that of the reference inhibitor. The 2D interaction maps and 3D binding poses of HPip-DC, HPip-4NO, and RDS1759 are shown in Figures S11–S16. The docking ranking did not fully correspond to the biochemical activity data, as HPip-DC was experimentally more active despite its less favorable docking score. Therefore, MD simulations were conducted to further assess the stability of the selected protein–ligand complexes.

### 3.6. Protein–Ligand Interaction Analysis

Protein–ligand interaction analysis was performed to characterize the predicted binding modes of the selected compounds on their respective protein targets (Figures S3–S16). In the predicted squalene monooxygenase model, HPip-DC formed one hydrogen bond with Tyr269 and several hydrophobic contacts with Leu267, Leu279, Leu450, Phe240, and Pro353 residues. Additional close contacts with Tyr107 and Tyr269, along with a halogen interaction involving Met447, further supported accommodation within the binding pocket. Amphotericin B formed a more extensive interaction network, including nine hydrogen bonds and hydrophobic contacts with Leu79, Leu267, Leu279, Phe240, and Pro82, consistent with its more favorable docking score (Table S4). Within bovine mitochondrial cytochrome bc_1_ (PDB ID: 6XVF), DiFiD is primarily stabilized by hydrophobic contacts with Ala84, Ala127, and Pro134, along with close contacts involving Gln44 and Tyr131 and a halogen interaction with Arg80. Atovaquone formed three hydrogen bonds with Arg80, Asn255, and Gly130, in addition to hydrophobic interactions with Ala84, Ala87, and Ala127. The more extensive polar interaction pattern of atovaquone was consistent with its more favorable predicted docking score compared to that of DiFiD (Table S4). The interactions of HPip-DC, HPip-4NO, and RDS1759 with HIV-1 RT-associated RNase H were comparatively limited. HPip-DC showed close contact with Asn474 and a π–cation interaction with Arg557, whereas HPip-4NO formed hydrophobic contacts with Ala445, Ala538, and Leu560. RDS1759 hydrophobically interacted with Ala538 and exhibited a close contact with Asn474. No qualifying hydrogen bonds, salt bridges, metal interactions, or water-mediated contacts were detected in any of the RNase H complexes (Table S4). Importantly, no clash-like contacts were observed in any of the complexes, supporting the geometric plausibility of the predicted binding sites. Overall, the interaction patterns were consistent with the docking results and provided a structural basis for selecting these complexes for subsequent MD simulation.

### 3.7. Molecular Dynamics, MM-GBSA, and Distance Analysis

The 300 ns MD simulations indicated that all four protein–ligand complexes remained structurally coherent, although their degrees of fluctuation differed (Figures S17–S20). The HIV-1 RT-associated RNase H complexes with HPip-4NO and HPip-DC showed the lowest mean protein RMSD values of 0.22 ± 0.03 nm, with ligand RMSD values of 0.16 ± 0.02 and 0.22 ± 0.02 nm, respectively. The bovine mitochondrial cytochrome *bc*_1_–DiFiD and squalene monooxygenase–HPip-DC complexes displayed higher mean protein RMSD values of 0.55 ± 0.08 and 0.65 ± 0.10 nm, respectively, whereas their ligand RMSD values remained comparatively low at 0.15 ± 0.03 and 0.20 ± 0.05 nm, respectively. The relatively stable RoG and SASA profiles further indicate the preservation of global protein compactness throughout the simulations. The squalene monooxygenase–HPip-DC complex showed the highest average number of hydrogen bonds (1.07 ± 0.70) and the shortest mean hydrophobic contact distance (0.20 ± 0.04 nm), followed by the cytochrome *bc*_1_–DiFiD complex with a hydrophobic contact distance of 0.22 ± 0.02 nm. These findings support stable ligand retention, particularly for HPip-DC, within the predicted squalene monooxygenase binding pocket.

MM-GBSA analysis predicted the most favorable binding energy for the predicted *L. major* squalene monooxygenase–HPip-DC complex, with a ΔG binding value of −33.41 kcal/mol. The DiFiD complex with the experimentally resolved bovine mitochondrial cytochrome *bc*_1_ complex, used as a structural reference for respiratory Complex III, showed a ΔG binding value of −9.29 kcal/mol. This result supports the stable accommodation of DiFiD within the examined bovine Complex III binding pocket but does not demonstrate engagement of the corresponding *T. gondii* cytochrome *bc*_1_ ortholog. Among the HIV-1 RNase H complexes, HPip-4NO exhibited a more favorable MM-GBSA binding energy estimate than HPip-DC, with ΔG binding values of −8.37 and −4.27 kcal/mol, respectively. Because the configurational entropy was not included, these values were interpreted comparatively rather than as absolute experimental binding affinity values.

### 3.8. Mode of Action Studies of HPip-DC and HPip-4NO Against HIV-1 RT-Associated RNase H

To validate the docking binding prediction (Figure 8) of the compounds in the active site of the enzyme, and the interaction with amino acid residue R557, we evaluated the activity of compounds HPip-DC and HPip-4NO in parallel against the RT WT (wildtype) enzyme and the HIV-1 RT R557A mutant (Table 4). The R557A amino acid substitution, introduced at a highly conserved site, was used as a functional tool to demonstrate that Arg557 is critical for inhibitor binding to RNase H [16].

The results confirmed the importance of residue Arg557 in binding of certain inhibitors and provided another hint at the predicted binding site to the enzyme. The significant loss of activity by HPip-4NO observed in the mutant suggests that this residue plays a direct role in ligand–protein interactions. The HPip-4NO compound was enzymatically active with the WT enzyme, whereas no enzymatic activity was observed with the R557A mutant, this difference was much less pronounced for the HPip-DC compound (nearly 2-fold) and only marginal for the reference compound RDS1759.

## 4. Discussion

Curcuminoid derivatives have attracted considerable interest due to their broad biological potential across infectious disease and cancer models. Several bis-arylidene piperidone derivatives, for example, displayed potent activity against *Leishmania major* promastigotes, rivaling the activity of previously reported aryl sulfonamide-modified piperidin-4-one curcuminoids [33]. Interestingly, the sulfonamide moiety was not essential for strong antileishmanial activity, suggesting that structural variations on the HPip scaffold can diversify the activity profiles. The motile promastigote stage is the infectious *L. major* stage, which is developed in the gut of sandfly vectors and characterized by distinct promastigote forms (procyclic and metacyclic promastigotes) [61]. Drugs targeting promastigotes in insect vectors might be useful tools for vector control (parasite cleansing in insect vectors) [62]. In terms of malaria therapy, the new promising fluorophenyl-substituted antimalarial drug candidate ganaplacide is effective against host infections and prevents parasite transmission which emphasizes the importance of transmission control in the management of parasitic infectious diseases [63,64].

Some of the highly anti-promastigote curcuminoids also displayed substantial activities against intramacrophageal amastigotes (IC_50_ values between 1.9 and 7 µM) which underscores their antileishmanial potential. Testing of some curcuminoids for toxicity to macrophages revealed relatively low toxicity and considerable selectivity indexes. Notably, amphotericin B was generally more toxic to macrophages than the tested curcuminoids. The toxic adverse effects of amphotericin B were published before which require formulation of amphotericin B in drugs for humans [7]. But amphotericin B was also more active against intramacrophageal *L. major* amastigotes than the curcuminoids. Thus, further research efforts are required to show if the curcuminoids have the potential to replace amphotericin B as an antileishmanial drug. It has to be mentioned that curcuminoids such as EF24 and DiFiD were well-tolerated by laboratory animals in in vivo studies before [29,30,31,65].

Although some curcuminoids were also active against *T. gondii* parasites, their selectivities in comparison with Vero kidney cells were only meager, which is also in line with previously studied curcuminoids [33]. It seems that kidney cells are more sensitive to curcuminoids than macrophages. In addition, all curcuminoids were more toxic to the kidney cells than atovaquone and, thus, future efforts might take care for a better tolerability in kidney cells. However, kidney cells are only one aspect of the complex renal organ system, and it has to be mentioned that curcuminoids can also be beneficial for the therapy of renal diseases and associated ailments (e.g., of chronic kidney disease) [66]. A more accurate assessment of the nephrotoxic properties of the curcuminoids used in this study might be achieved by tests in more organ-like models (e.g., kidney organoids with nephron structures) [67].

It is noteworthy that certain curcuminoid derivatives can also inhibit viral enzymes such as HIV-1 RT-associated RNase H. To our knowledge, this is the first report of monoketone-based curcuminoids with HIV-1 RNase H-inhibitory activities. Before, curcuminoid derivatives were shown to inhibit the DNA integrase HIV-1 IN [37]. Two promising RNase H-inhibitory curcuminoids (HPip-DC and HPip-4NO) were selected and tested for HIV replication inhibition. A considerable inhibition (approx. 40%) of HIV replication at a non-toxic concentration was only observed for HPip-4NO while HPip-DC turned out to be too toxic. Since HPip-4NO was only moderately toxic to macrophages, the application of other cell models instead of the cancer-derived HeLa-P4 cells might lead to improved selectivity. Moreover, formulation and chemical fine-tuning of the active curcuminoids might be further suitable strategies to improve compound safety [27,34]. The high activities of HPip-DC and HPip-4NO against HeLa-derived cells might also be of interest in terms of a possible development of curcuminoids as anticancer agents [34].

The relevance of the described HIV-1 RNase H-inhibitory activities is supported by the fact that eukaryotic cells also express vital RNase H enzymes [68]. For instance, RNase H1 is an essential enzyme and a possible drug target in *Leishmania* parasites [69,70,71]. The viability of *T. gondii* cells also depends on ribonucleases such as TSN [72]. RNase H enzymes share similar structures and nuclease mechanisms, and thus protozoal RNase H might also be a target of curcuminoids. However, a clear correlation between antiparasitic activity and HIV-1 RNase H inhibition could not be seen because some strongly antileishmanial curcuminoids such as HPip-2Cl only exhibited weak HIV-1 RNase H inhibition.

The structure–activity relationships (SARs) observed in related studies highlight the beneficial role of *para*-substituents (e.g., halogens, nitro groups) in enhancing RNase H inhibition. Importantly, some scaffolds such as HPip-4NO, HPip-DC, and HPip-4Br demonstrated activity against *L. major* promastigotes and HIV-1 RNase H, pointing to the feasibility of developing both antiparasitic and antiviral agents. Hence, 4-nitrophenyl, 3,4-dichlorophenyl and 4-bromophenyl moieties are promising scaffolds for the design of antileishmanial and antiviral drug candidates. A possible dual-targeting concept is supported by earlier reports on coumarin derivatives tested against *Leishmania amazonensis* promastigotes and herpes simplex virus, although their activities were weaker than those observed for EF24-type curcuminoids [73]. Surely, the toxicity of the curcuminoids HPip-4NO and HPip-DC to viral host cells should be reduced in structurally optimized derivatives. Nevertheless, compounds such as EF24 have shown reasonable tolerability in laboratory animals and, thus, it should be possible to design promising antiviral non-toxic curcuminoids with HIV-1 RNase H-inhibitory activities in the future [65].

**Figure 9:**
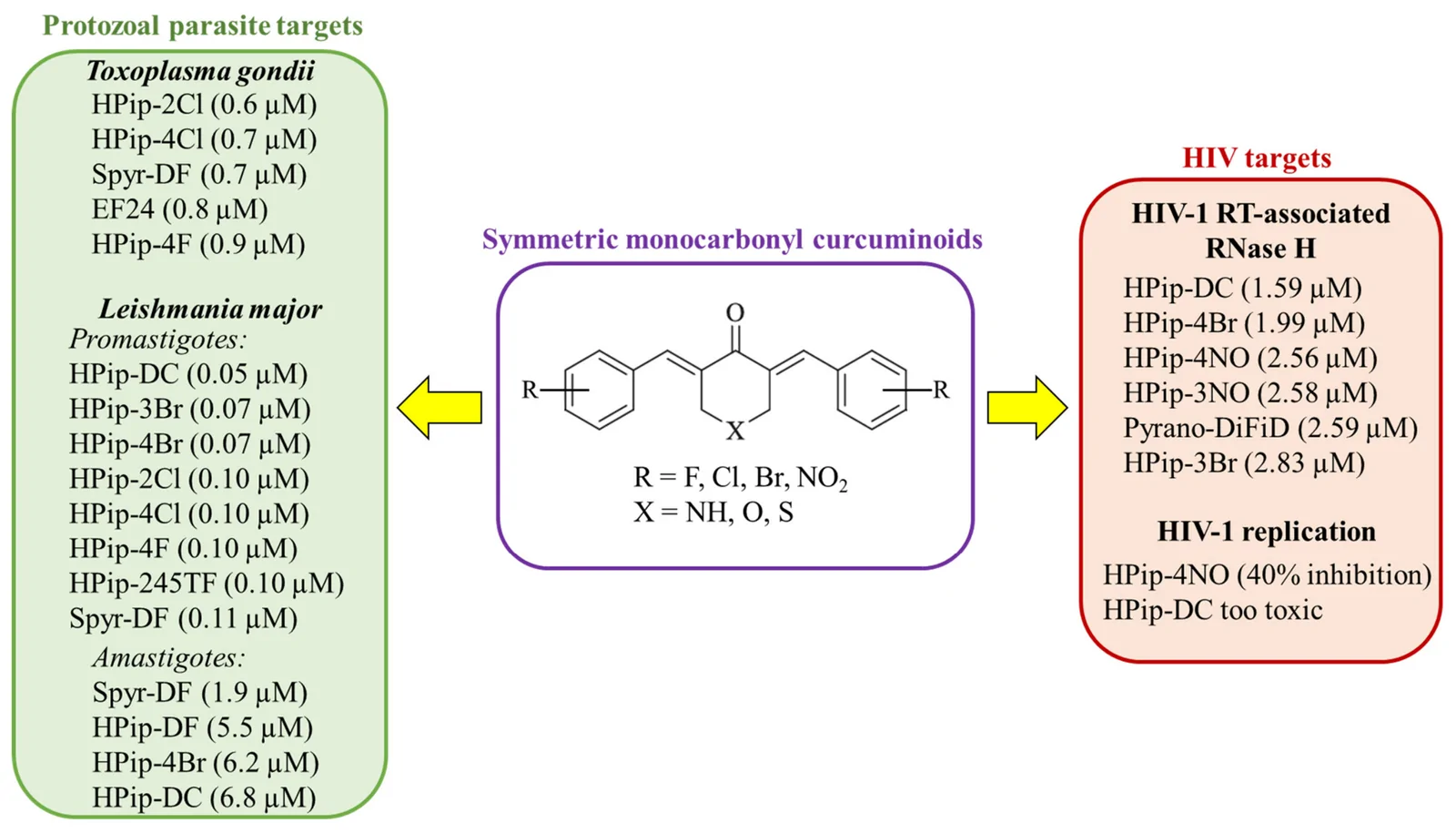
Graphical summary of the most active curcuminoids (IC_50_ values or inhibition percentage in brackets) and their protozoal parasite (*T.gondii* and *L. major*) and HIV targets.

Figure 9 summarizes the most active curcuminoids against protozoal parasite (*L. major* and *T. gondii*) and HIV targets (HIV-1 RT-associated RNase H, virus replication), which were identified in this study.

The docking analysis generated useful target hypotheses but should be interpreted as a prioritization exercise rather than proof of the mechanism. DiFiD showed favorable predicted binding to several *T. gondii*-associated proteins, particularly ROP5C, prolyl-tRNA synthetase, and CDPK1, suggesting that its antitoxoplasmal activity may involve multiple cellular pathways. The bovine mitochondrial cytochrome bc_1_ structure represented by PDB 6XVF was included only as a mammalian structural reference and should not be described as a *T. gondii* target protein. Although DiFiD occupied a pocket similar to that used by quinolone inhibitors, its weaker docking score compared to atovaquone and the use of a bovine rather than a parasite protein prevented the direct assignment of cytochrome *bc*_1_ as its molecular target. For HPip-DC, squalene monooxygenase was the highest-ranked *Leishmania* protein, which is biologically plausible because sterol biosynthesis is essential in kinetoplastids, and squalene monooxygenase/ERG1 has been associated with terbinafine susceptibility in *L. infantum* [74]. Nevertheless, this target was represented by a predicted model and requires further experimental validation. The strongly negative score obtained for amphotericin B should also be interpreted cautiously because amphotericin B is considerably larger than HPip-DC and exerts its established activity predominantly through membrane sterol binding rather than by direct inhibition of squalene monooxygenase. Finally, the small differences among the RNase H docking scores of HPip-4NO, RDS1759, and HPip-DC were within the uncertainty of the conventional docking functions and did not provide a reliable quantitative ranking of their biochemical potency.

Protein–ligand interaction analysis supports plausible binding poses while revealing important differences from the earlier mechanistic interpretation. HPip-DC formed a hydrogen bond with Tyr269 and hydrophobic contacts with Leu267, Leu279, Leu450, Phe240, and Pro353 within the predicted squalene monooxygenase pocket, whereas DiFiD was stabilized mainly through hydrophobic and close contacts within the bovine cytochrome *bc*_1_ reference structure. In the RNase H complexes, HPip-DC displayed close contact with Asn474 and a π–cation interaction with Arg557, whereas HPip-4NO was associated mainly with hydrophobic contacts involving Ala445, Ala538, and Leu560. No qualifying hydrogen bonds, salt bridges, catalytic metal interactions, or water-mediated contacts were detected in the RNase H complexes. Accordingly, these compounds should not be described as metal-chelating active-site inhibitors.

To validate the binding model, the R557A amino acid substitution was introduced to assess the impact of that interaction with the ligand and corroborate the binding hypothesis. The residue is located in the remote extremity of the RNase H domain. Arg557 is primarily involved in correct substrate positioning and the catalytic mechanism, rather than in maintaining the overall structural integrity of the RNase H domain. Interestingly, the R557A substitution caused a greater than 11.72-fold reduction in HPip-4NO activity despite the absence of a direct Arg557 contact in its static docking pose, whereas HPip-DC, for which a π–cation interaction with Arg557 was predicted, showed only a 1.81-fold shift. This discrepancy emphasizes that the results of mutant enzymes should not be interpreted as proof of a specific ligand–residue contact. Arg557 may indirectly influence inhibition by contributing to the local electrostatic environment, catalytic site organization, or conformational stability of the RNase H region. Consequently, the combined docking and R557A data support a testable binding hypothesis but do not establish the molecular mechanism of the inhibition. The minimal 1.17-fold effect on RDS1759 agrees with previous site-directed mutagenesis findings, showing that RDS1759 activity is largely insensitive to R557A mutation [47]. The mechanistic interpretation of HIV-1 RNase H inhibition has several limitations. Although the R557A mutant experiment demonstrated a compound-dependent change in inhibitor sensitivity, inhibition kinetics were not performed to distinguish between competitive, non-competitive, uncompetitive, or mixed inhibition. Likewise, Mg^2+^-dependent experiments were not conducted, and direct protein–ligand binding was not measured using biophysical methods such as surface plasmon resonance, isothermal titration calorimetry, microscale thermophoresis, or differential scanning fluorimetry. In addition, the compounds were not counter-screened against the DNA polymerase activity of full-length HIV-1 reverse transcriptase. Therefore, the present evidence demonstrates the biochemical inhibition of HIV-1 RT-associated RNase H and residue-dependent effects associated with the R557A substitution; however, it does not definitively establish the binding mechanism, inhibition mode, or functional selectivity for RNase H catalytic activity.

The 300 ns simulations indicated persistent ligand retention in all four selected complexes, although the magnitude of protein fluctuations differed among the systems. RNase H complexes with HPip-4NO and HPip-DC displayed low mean protein RMSD values of approximately 0.22 nm, along with correspondingly low ligand RMSD values, indicating stable ligand accommodation within the simulated binding pockets. DiFiD also maintained a low ligand RMSD in the bovine cytochrome *bc*_1_ reference complex. In contrast, the predicted squalene monooxygenase–HPip-DC complex exhibited a higher protein RMSD, whereas HPip-DC remained associated with the binding pocket through persistent hydrophobic contacts and showed the highest average number of protein–ligand hydrogen bonds among the four simulated complexes. The stable RoG and SASA profiles suggest that no major global unfolding occurred during the experiment. MM-GBSA ranked the predicted squalene monooxygenase–HPip-DC complex as the most favorable, followed by the cytochrome bc_1_–DiFiD, RNase H–HPip-4NO, and RNase H–HPip-DC complexes. However, the RNase H MM-GBSA ranking did not reproduce the experimental inhibitory ranking because HPip-DC was more active than HPip-4NO. Time-resolved distance analyses provide additional evidence of ligand rearrangement and persistent residue proximity but do not establish direct hydrogen bonding or target inhibition.

Although the monitored structural descriptors indicated relatively stable behavior during the analyzed trajectories, these observations should not be interpreted as evidence of the formal simulation convergence. The 300 ns timescale may not fully sample slower conformational transitions or alternative protein–ligand binding states. Moreover, because only one independent production trajectory was performed for each selected complex, the reproducibility across independently initialized replicate simulations could not be assessed. Longer simulations and independent replicate trajectories would therefore be valuable for evaluating the robustness of observed interaction patterns and energetic trends.

Several translational and lead optimization limitations should be considered when interpreting the present findings. No in vivo efficacy studies have been performed, and compound-specific pharmacokinetic parameters, metabolic stability, plasma stability, and bioavailability have not been evaluated. In addition, the physicochemical properties relevant to drug development, including aqueous solubility, were not systematically characterized, microsomal stability and potential metabolic liabilities were not investigated, and selectivity was evaluated using only a limited number of mammalian cell models. Although several compounds exhibited high potency against *L. major* promastigotes, their activity against the clinically relevant intracellular amastigote stage was generally weaker. Consequently, promastigote potency alone should not be interpreted as evidence of lead-like or therapeutic potential. Although previous studies have reported in vivo tolerability and pharmacokinetic information for curcuminoids such as EF24 and DiFiD, these findings cannot be directly extrapolated to the individual analogs investigated here. Therefore, the compounds identified in this study should be regarded as chemical starting points for further optimization and pharmacological evaluation, rather than validated or development-ready therapeutic leads.

Taken together, the present in vitro findings indicate that relatively modest structural modifications of the EF24/DiFiD-derived monoketone scaffold are associated with distinct antitoxoplasmal, antileishmanial, RNase H-inhibitory, and preliminary antiviral activity. HPip-DC and HPip-4Br are particularly notable for their combined activity against *L. major* promastigotes and HIV-1 RNase H, whereas HPip-4NO combines RNase H inhibition with measurable antiviral activity and a pronounced dependence on Arg557. Nevertheless, the compounds described herein should be considered as chemical starting points rather than validated therapeutic leads, as host cell toxicity, modest intracellular antiparasitic activity, and incomplete target validation remain important limitations together with the absence of data referring to pharmacokinetics, metabolic stability, plasma stability, and bioavailability of the curcuminoids. The proposed *Leishmania* and *T. gondii* targets should be investigated using purified enzyme assays, target overexpression or knockdown, resistance selection, metabolomic profiling, and sterol-rescue experiments. Further antiviral studies should include human RNase H selectivity, complete concentration–response analysis, testing against full-length RT, and evaluation in physiologically relevant host cell systems. In terms of the mechanism of HIV-1 RNase H inhibition by the described curcuminoids, the presented experimental results support biochemical inhibition of this viral enzyme but do not definitively establish the binding mechanism. More experiments including kinetic characterization distinguishing competitive, non-competitive or mixed inhibition, metal-dependence experiments, direct binding measurements (SPR, ITC, MST or differential scanning fluorimetry), and/or counterscreening against the DNA polymerase activity of HIV-1 reverse transcriptase are required. Medicinal chemistry optimization should focus on reducing nonspecific cytotoxicity while maintaining the favorable *para*-halogenated, 3,4-dichlorinated, and nitro-substituted patterns identified in this study.

## 5. Conclusions

DiFiD provided the most favorable balance between antitoxoplasmic activity and Vero cell selectivity. HPip-4NO showed modest inhibition of HIV-1 replication at a non-cytotoxic concentration, and the marked loss of activity against the R557A mutant identified Arg557 as an important compound-specific determinant of inhibition. Computational analyses supported the persistent binding of selected compounds and generated testable target hypotheses, particularly squalene monooxygenase for HPip-DC. However, these predictions require direct biochemical confirmation. Overall, this study provides a useful structure–activity framework for developing safer and more selective monoketone curcuminoids with antiprotozoal and HIV-1 RNase H-inhibitory properties. Thus, the identified active curcuminoids can serve as promising chemical starting points for the design of antiviral and antiparasitic lead compounds in the future.

## Figures and Tables

**Table 1:** Activities (expressed as IC_50_ values in µM ± SD, *n* = 3) of test compounds against *T. gondi* parasites and Vero monkey kidney cells. Atovaquone (ATO) was used as positive control.

Compounds	*T. gondii*	Vero	SI ^1^
EF24	0.8 ± 0.2 **	1.2 ± 0.3 **	1.2
DiFiD	1.2 ± 0.2 **	3.7 ± 0.6 *	3.1
HPip-3F	1.3 ± 0.3 **	0.6 ± 0.1 **	0.5
HPip-4F	0.9 ± 0.2 **	1.3 ± 0.2 **	1.4
HPip-DF	3.2 ± 0.5 *	1.2 ± 0.2 **	0.4
HPip-23DF	1.7 ± 0.3 **	1.7 ± 0.3 **	1.0
HPip-25DF	2.0 ± 0.4 **	1.5 ± 0.3 **	0.8
HPip-245TF	2.4 ± 0.5 **	0.8 ± 0.1 **	0.3
HPip-246TF	4.8 ± 0.6 *	7.2 ± 1.0	1.5
HPip-ClF	2.4 ± 0.4 **	1.5 ± 0.3 **	0.6
HPip-MeOF	10.5 ± 1.8	1.8 ± 0.4 **	0.2
HPip-2Cl	0.6 ± 0.1 **	0.7 ± 0.1 **	1.2
HPip-4Cl	0.7 ± 0.1 **	1.1 ± 0.2 **	1.6
HPip-DC	3.0 ± 0.5 *	3.0 ± 0.5 *	1.0
HPip-3Br	2.6 ± 0.4 **	2.8 ± 0.5 **	1.1
HPip-4Br	1.0 ± 0.2 **	0.9 ± 0.2 **	0.9
HPip-3NO	3.6 ± 0.7 *	3.3 ± 0.3 *	0.9
HPip-4NO	6.3 ± 0.9 *	1.4 ± 0.2 **	0.2
Spyr-EF24	3.7 ± 0.6 *	4.0 ± 0.7 *	1.1
Spyr-DF	0.7 ± 0.2 **	0.8 ± 0.2 **	1.1
Thio-DiFiD	5.3 ± 1.1 *	6.5 ± 0.9 *	1.2
Pyrano-DiFiD	1.9 ± 0.2 **	2.1 ± 0.3 **	1.1
ATO ^2^	0.07 ± 0.01 **	9.5 ± 1.6	135

^1^ SI = selectivity index (Vero/*T. gondii*). ^2^ Values also present in ref. [33]. *t*-test was used to compare significant differences with the control (* *p* < 0.05, ** *p* < 0.01).

**Table 2:** Activities (expressed as IC_50_ values in µM ± SD, *n* = 3) of test compounds against *L. major* parasites (promastigotes and amastigotes) and murine macrophages. Amphotericin B (AmB) was used as a positive control.

Compounds	Promastigotes	Amastigotes	Macrophages	SI (Macrophages) ^1^	SI (Vero) ^2^
EF24	0.90 ± 0.2 **	9.0 ± 1.7 *	-	-	1.3
DiFiD	0.20 ± 0.03 **	7.8 ± 1.4 *	-	-	3.1
HPip-3F	0.45 ± 0.1 **	48.5 ± 6.9	22.8 ± 3.5	51	1.3
HPip-4F	0.10 ± 0.03 **	7.7 ± 1.5 *	-	-	13
HPip-DF	0.32 ± 0.1 **	5.5 ± 1.3 *	19.6 ± 2.8	61	3.8
HPip-23DF	0.58 ± 0.2 **	41.5 ± 7.4	19.3 ± 3.1	33	2.9
HPip-25DF	0.29 ± 0.06 **	18.1 ± 3.7	16.7 ± 1.9	58	6
HPip-245TF	0.10 ± 0.02 **	14.4 ± 2.2	16.2 ± 2.3	162	8
HPip-246TF	10.8 ± 1.6	8.1 ± 1.8 *	-	-	0.7
HPip-ClF	0.24 ± 0.04 **	13.7 ± 2.5	17.9 ± 3.0	75	6.3
HPip-MeOF	0.24 ± 0.03 **	23.4 ± 4.0	24.8 ± 3.2	103	7.5
HPip-2Cl	0.10 ± 0.02 **	9.0 ± 1.6 *	-	-	7
HPip-4Cl	0.10 ± 0.01 **	7.6 ± 1.1 *	-	-	11
HPip-DC	0.05 ± 0.01 **	6.8 ± 1.2 *	-	-	60
HPip-3Br	0.07 ± 0.02 **	17.8 ± 2.9	-	-	40
HPip-4Br	0.07 ± 0.01 **	6.2 ± 1.3 *	-	-	12.9
HPip-3NO	0.22 ± 0.05 **	8.5 ± 1.6 *	-	-	15
HPip-4NO	0.22 ± 0.03 **	33.1 ± 5.7	18.3 ± 3.3	83	6.4
Spyr-EF24	0.94 ± 0.2 **	8.8 ± 1.2 *	-	-	0.85
Spyr-DF	0.11 ± 0.01 **	1.9 ± 0.4 **	-	-	7.3
Thio-DiFiD	1.1 ± 0.2 **	24.4 ± 3.3	-	-	5.9
Pyrano-DiFiD	1.0 ± 0.1 **	7.8 ± 1.2 *	-	-	2.1
AmB ^3^	0.83 ± 0.1 **	0.47 ± 0.07 **	8.1 ± 1.5	9.8	-

^1^ SI = selectivity index (macrophages/*L. major* promastigotes). ^2^ SI = selectivity index (Vero kidney cells/*L. major* promastigotes). ^3^ Values also present in ref. [33]. *t*-test was used to compare significant differences with the control (* *p* < 0.05, ** *p* < 0.01).

**Table 3:** Inhibition (expressed as IC_50_ values in µM, *n* = 3) of HIV-1 RT-associated RNase H by test compounds. RDS1759 was used as positive control.

Compounds	HIV-1 RNase H IC_50_ (µM)	Compounds	HIV-1 RNase H IC_50_ (µM)
EF24	>100	HPip-2Cl	>30
DiFiD	5.37 ± 0.43	HPip-4Cl	6.88 ± 0.50
HPip-3F	22.98 ± 1.34	HPip-DC	1.59 ± 0.36
HPip-4F	5.96 ± 1.74	HPip-3Br	2.83 ± 0.46
HPip-DF	5.15 ± 1.07	HPip-4Br	1.99 ± 0.18
HPip-23DF	16.20 ± 2.08	Hpip-3NO	2.58 ± 0.36
HPip-25DF	23.32 ± 4.15	HPip-4NO	2.56 ± 0.15
HPip-245TF	6.63 ± 0.59	Spyr-EF24	8.65 ± 0.57
HPip-246TF	18.65 ± 4.16	Spyr-DF	13.85 ± 5.05
HPip-ClF	3.56 ± 0.76	Thio-DiFiD	8.48 ± 0.85
HPip-MeOF	7.55 ± 0.97	Pyrano-DiFiD	2.59 ± 0.37
		RDS1759	5.8 ± 2.8

**Table 4:** IC_50_ values (µM, *n* = 3) for WT and mutant R557A and corresponding fold shift (R557A/WT).

Compounds	HIV-1 RNase H WT IC_50_ (μM)	HIV-1 RNase H R557A IC_50_ (μM)	Fold Change (R557A/WT)
HPip-4NO	2.56 ± 0.15	>30	>11.72
HPip-DC	1.59 ± 0.36	2.87 ± 0.41	1.81
RDS1759	5.8 ± 2.8	6.8 ± 2.8	1.17

## Data Availability

Original data can be obtained from the authors upon reasonable request.

## Supplementary Materials

The following supporting information can be downloaded at: https://www.mdpi.com/article/10.3390/pathogens15080876/s1, dose–response curves, interference check and protein docking information, synthetic procedure and analysis of new compound Thio-DiFiD, analyses of known test compounds (^1^H NMR, elemental analysis or HR-MS data). Tables S1–S3: Protein information for molecular docking; Table S4: Predicted protein–ligand interactions; Figures S1–S16: Molecular docking scores and images; Figures S17–S24: Molecular dynamics analyses; Figures S25–S29: Dose–response curves of experiments with *L. major*, *T. gondii*, murine macrophages and Vero cells; Figure S30: Dose–response curves of HIV-1 RNase H inhibition experiments; Figure S31: Fluorescence interference check; Figures S32–S54: Original NMR spectra of test compounds; Figures S55 and S56: HR-MS (ESI) spectra of HPip-DC and HPip-4NO.

## Abbreviations

The following abbreviations are used in this manuscript:

AIDS	Acquired immunodeficiency syndrome
CL	Cutaneous leishmaniasis
CPRG	Chlorophenol red-β-D-galactopyranoside
HAART	Highly active antiretroviral therapy
HIV	Human immunodeficiency virus
IN	Integrase
MD	Molecular dynamics
MM-GBSA	Molecular Mechanics Generalized Born Surface Area
MOE	Molecular Operating Environment
MTT	3-(4,5-Dimethylthiazol-2-yl)-2,5-diphenyltetrazolium bromide
NTD	Neglected tropical disease
RMSD	Root-mean-square deviation
RMSF	Root-mean-square fluctuation
RNase H	Ribonuclease H
RoG	Radius of gyration
RT	Reverse transcriptase
SAR	structure–activity relationship
SASA	Solvent-accessible surface area
TSN	Tudor staphylococcal nuclease

## References

[1] Baker R.E., Mahmud A.S., Miller I.F., Rajeev M., Rasambainarivo F., Rice B.L., Takahashi S., Tatem A.J., Wagner C.E., Wang L.-F. (2022). Infectious disease in an era of global change. Nat. Rev. Microbiol..

[2] Kampf G., Brüggemann Y., Kaba H.E.J., Steinmann J., Pfaender S., Scheithauer S., Steinmann E. (2020). Potential sources, modes of transmission and effectiveness of prevention measures against SARS-CoV-2. J. Hosp. Infect..

[3] Pozzoli A.V., Alho M.A.M., Rivera N. (2016). Drugs for parasitic diseases—Strategies to solve a global problem. Antiprotozoal Drug Discovery: A Challenge That Remains.

[4] Mitra A.K., Mawson A.R. (2017). Neglected tropical diseases: Epidemiology and global burden. Trop. Med. Infect. Dis..

[5] Leishmaniasis. https://www.who.int/news-room/fact-sheets/detail/leishmaniasis.

[6] Burza S., Croft S.L., Boelaert M. (2018). Leishmaniasis. Lancet.

[7] Garza-Tovar T.F., Sacriste-Hernández M.I., Juárez-Durán E.R., Arenas R. (2020). An overview of the treatment of cutaneous leishmaniasis. Fac. Rev..

[8] Bacha T.d.J., Comandolli-Wyrepkowski C.D., Jensen B.B., Franco A.M.R. (2021). Current situation of cutaneous leishmaniasis and challenges of drug resistance: A narrative review. Eur. Acad. Res..

[9] Seeber F., Steinfelder S. (2016). Recent advances in understanding apicomplexan parasites. F1000Research.

[10] Dunay I.R., Gajurel K., Dhakal R., Liesenfeld O., Montoya J.G. (2018). Treatment of toxoplasmosis: Historical perspective, animal models, and current clinical practice. Clin. Microbiol. Rev..

[11] UNAIDS Global HIV & AIDS Statistics—Fact Sheet. https://www.unaids.org/en/resources/fact-sheet.

[12] Modrow S., Falke D., Truyen U., Schätzl H. (2010). Molekulare Virologie.

[13] Singh E.A., Ningthoujam D.S., Devi L.J., Khunjamayum R., Ningombam D., Singh L.S., Tamreihao K., Mukhrejee S., Ningthoujam D.S. (2019). Therapeutic drugs used for effective lifelong control of HIV and AIDS. Frontiers in Anti-Infective Agents.

[14] Alvar J., Aparicio P., Aseffa A., den Boer M., Canavate C., Dedet J.-P., Gradoni L., ter Horst R., López-Vélez R., Moreno J. (2008). The relationship between leishmaniasis and AIDS: The second 10 years. Clin. Microbiol. Rev..

[15] Nuwagaba J., Li J.A., Ngo B., Sutton R.E. (2025). 30 years of HIV therapy: Current and future antiviral drug targets. Virology.

[16] Corona A., Ballana E., Distino S., Rogolino D., del Vecchio C., Carcelli M., Badia R., Riveira-Munoz E., Esposito F., Parolin C. (2020). Targeting HIV-1 RNase H: *N’*-(2-hydroxy-benzylidene)-3,4,5-trihydroxybenzoylhydrazone as selective inhibitor active against NNRTIs-resistant variants. Viruses.

[17] Tramontano E., Corona A., Menéndez-Arias L. (2019). Ribonuclease H, an unexploited target for antiviral intervention against HIV and hepatitis B virus. Antivir. Res..

[18] Xu L., Grandi N., del Vecchio C., Mandas D., Corona A., Piano D., Esposito F., Parolin C., Tramontano E. (2015). From the traditional Chinese medicine plant *Schisandra chinensis* new scaffolds effective on HIV-1 reverse transcriptase resistant to non-nucleoside inhibitors. J. Microbiol..

[19] Sanna C., Scognamiglio M., Fiorentino A., Corona A., Graziani V., Caredda A., Cortis P., Montisci M., Ceresola E.R., Canducci F. (2018). Prenylated phloroglucinols from *Hypericum scruglii*, an endemic species of Sardinia (Italy), as new dual HIV-1 inhibitors effective on HIV-1 replication. PLoS ONE.

[20] Corona A., Seibt S., Schaller D., Schobert R., Volkamer A., Biersack B., Tramontano E. (2021). Garcinol from *Garcinia indica* inhibits HIV-1 reverse transcriptase-associated ribonuclease H. Arch. Pharm..

[21] Goel A., Kunnumakkara A.B., Aggarwal B.B. (2008). Curcumin as “curecumin’’: From kitchen to clinic. Biochem. Pharmacol..

[22] Shaikh S., Shaikh J., Naba Y.S., Doke K., Ahmed K., Yusufi M. (2021). Curcumin: Reclaiming the lost ground against cancer resistance. Cancer Drug Resist..

[23] Kesharwani R.K., Misra K., Singh D.B. (2019). Perspectives and challenges of tropical medicinal herbs and modern drug discovery in the current scenario. Asian Pac. J. Trop. Med..

[24] Cheraghipour K., Marzban A., Ezatpour B., Khanizadeh S., Koshki J. (2018). Antiparasitic properties of curcumin: A review. AIMS Agric. Food.

[25] Praditya D., Kirchhoff L., Brüning J., Rachmawati H., Steinmann J., Steinmann E. (2019). Anti-infective properties of the golden spice curcumin. Front. Microbiol..

[26] Jennings M.R., Parks R.J. (2020). Curcumin as an antiviral agent. Viruses.

[27] Hegde M., Girisa S., Chetty B.B., Vishwa R., Kunnumakkara A.B. (2023). Curcumin formulations for better bioavailability: What we learned from clinical trials so far?. ACS Omega.

[28] Kasinski A.L., Du Y., Thomas S.L., Zhao J., Sun S.-Y., Khuri F.R., Wang C.-Y., Shoji M., Sun A., Snyder J.P. (2008). Inhibition of IκB kinase-nuclear factor-κB signaling pathway by 3,5-bis(2-flurobenzylidene)piperidin-4-one (EF24), a novel monoketone analog of curcumin. Mol. Pharmacol..

[29] Reid J.M., Buhrow S.A., Gilbert J.A., Jia L., Shoji M., Snyder J.P., Ames M.M. (2014). Mouse pharmacokinetics and metabolism of the curcumin analog, 4-piperidinone,3,5-bis[(2-fluorophenyl)methylene]-acetate(3E,5E) (EF24; NSC716993). Cancer Chemother. Pharmacol..

[30] Subramaniam D., Nicholes N.D., Dhar A., Umar S., Awasthi V., Welch D.R., Jensen R.A., Anant S. (2011). 3,5-bis(2,4-difluorobenzylidene)-4-piperidone, a novel compound that affects pancreatic cancer growth and angiogenesis. Mol. Cancer Ther..

[31] Standing D., Arnold L., Dandawate P., Ottemann B., Snyder V., Ponnurangam S., Sayed A., Subramaniam D., Srinivasan P., Choudhury S. (2023). Doublecortin-like kinase 1 is a therapeutic target in squameous cell carcinoma. Mol. Carcinog..

[32] Lima R.H., Robles Y.R., Oliva I.M., Santos A.L.O., Teixeira J.G., Chellegatti M.A.S.C., Furtado N.A.J.C., Martins C.H.G., Nardini V., Crotti A.E.M. (2025). Antimicrobial activity of *N*-methyl 4-piperidone-derived monoketone curcuminoids against cariogenic bacteria. Future Pharmacol..

[33] Al Nasr I.S., Hanachi R., Said R.B., Rahali S., Tangour B., Abdelwahab S.I., Farasani A., Taha M.M.E., Bidwai A., Koko W.S. (2021). *p*-Trifluoromethyl and *p*-pentafluorothio-substituted curcuminoids of the 2,6-di[(*E*)-benzylidene)]cycloalkanone type: Syntheses and activities against *Leishmania major* and *Toxoplasma gondii* parasites. Bioorg. Chem..

[34] Vieira T.M., Tanajura L.S., Heleno V.C.G., Magalhães L.G., Crotti A.E.M. (2024). Monoketone curcuminoids: An updated review of their synthesis and biological activities. Future Pharmacol..

[35] Souza J.M., Vieira T.M., Candido A.C.B.B., Tezuka D.Y., Rao G.S., de Albuquerque S., Crotti A.E.M., Siqueira-Neto J.L., Magalhães L.G. (2021). In vitro anti-*Trypanosoma cruzi* activity enhancement of curcumin by its monoketone tetramethoxy analog diveratralacetone. Curr. Res. Parasitol. Vector Borne Dis..

[36] Courvoisier-Dezord E., Ragusa H., Grandé A., Denudt L., Charmasson Y., Dumur F., Siri D., Maresca M., Nechab M. (2025). 1,5-Diarylidene-4-piperidones as promising antifungal candidates against *Cryptococcus neoformans*. Antibiotics.

[37] Costi R., di Santo R., Artico M., Massa S., Ragno R., Loddo R., la Colla M., Tramontano E., la Colla P., Pani A. (2004). 2,6-Bis(3,4,5-trihydroxybenzylydene) derivatives of cyclohexanone: Novel potent HIV-1 integrase inhibitors that prevent HIV-1 multiplication in cell-based assays. Bioorg. Med. Chem..

[38] Karki S.S., Das U., Balzarini J., De Clercq E., Sakagami H., Uesawa Y., Roayapalley P.K., Dimmock J.R. (2024). Does ortho substitution enhance cytotoxic potencies in a series of 3,5-bis(benzylidene)-4-piperidones?. Medicines.

[39] Ghosh H., Bhattacharyya S., Schobert R., Dandawate P., Biersack B. (2023). Fluorinated and N-acryloyl-modified 3,5-di[(*E*)benzylidene]piperidin-4-one curcuminoids for the treatment of pancreatic carcinoma. Pharmaceutics.

[40] Tan K.-L., Ali A., Du Y., Fu H., Jin H.-X., Chin T.-M., Khan M., Go M.-L. (2014). Synthesis and evaluation of bisbenzylidenedioxotetrahydrothiopranones as activators of endoplasmic reticulum (ER) stress signaling pathways and apoptotic cell death in acute promyelocytic leukemic cells. J. Med. Chem..

[41] Schmitt F., Gold M., Begemann G., Andronache I., Biersack B., Schobert R. (2017). Fluoro and pentafluorothio analogs of the antitumoral curcuminoid EF24 with superior antiangiogenic and vascular-disruptive effects. Bioorg. Med. Chem..

[42] Snyder J.P., Davis M.C., Adams B., Shoji M., Liotta D., Ferstl E.M., Sunay U.B. (2001). Curcumin analogs with anti-tumor and an-ti-angiogenic properties.

[43] Das S., Das U., Sakagami H., Hashimoto K., Kawase M., Gorecki D.K.J., Dimmock J.R. (2010). Sequential cytotoxicity: A theory examined using a series of 3,5-bis(benzylidene)-1-diethylphosphono-4-oxopiperidines and related phosphonic acids. Bioorg. Med. Chem. Lett..

[44] Gregory M., Dandavati A., Lee M., Tzou S., Savagian M., Brien K.A., Satam V., Patil P., Lee M. (2013). Synthesis, cytotoxicity, and structure-activity insight of NH- and N-methyl-3,5-bis-(arylidenyl)-4-piperidones. Med. Chem. Res..

[45] Almansour A.I., Kumar R.S., Beevi F., Shirazi A.N., Osman H., Ismail R., Choon T.S., Sullivan B., McCaffrey K., Nahhas A. (2014). Facile, regio- and diastereoselective synthesis of spiro-pyrrolidine and pyrrolizine derivatives and evaluation of their antiproliferative activities. Molecules.

[46] Tu N.Q., Richetta C., Putzu F., Delelis O., Ahmed K., Masand V.H., Schobert R., Tramontano E., Corona A., Biersack B. (2025). Identification of HIV-1 reverse transcriptase-associated ribonuclease H inhibitors based on 2-hydroxy-1,4-naphthoquinone Mannich bases. Molecules.

[47] Corona A., di Leva F.S., Thierry S., Pescatori L., Crucitti G.C., Subra F., Delelis O., Esposito F., Rigogliuso G., Costi R. (2014). Identification of highly conserved residues involved in inhibition of HIV-1 RNase H function by diketo acid derivatives. Antimicrob. Agents Chemother..

[48] Charneau P., Alizon M., Clavel F. (1992). A second origin of DNA plus-strand synthesis is required for optimal human immunodeficiency virus replication. J. Virol..

[49] (2022). Molecular Operating Environment (MOE), 2022.02.

[50] Qureshi K.A., Parvez A. (2026). Antimicrobial and antibiofilm evaluation of thymol, sodium azide, and sodium lauryl sulfate against multidrug-resistant pathogens: An integrated experimental and computational study. PLoS ONE.

[51] Qureshi K.A., Parvez A., Ismatullah H., Almahasheer H., Rugaie O.A. (2025). Exploring the antimicrobial and antibiofilm potency of four essential oils against selected human pathogens using in vitro and in silico approaches. PLoS ONE.

[52] Qureshi K.A., Parvez A., Jaremko M. (2025). Repurposing eugenol and cinnamaldehyde as potent antimicrobial agents: A comprehensive in vitro and in silico study. Bioorg. Chem..

[53] Ali A., Qureshi K.A., Banerjee B., Verma R.K., Maurya N., Nair K.N., Verma V.K., Singh R.C., Pandey P., Alharbi A.M. (2026). Asymmetric organotellurium hard-soft donor Schiff base ligand and its Cu(II)/Ni(II) complexes: DNA intercalation, anti-inflammatory and antimicrobial activity. Polyhedron.

[54] wwPDB Consortium (2019). Protein Data Bank: The single global archive for 3D macromolecular structure data. Nucleic Acids Res..

[55] Waterhouse A., Bertoni M., Bienert S., Studer G., Tauriello G., Gumienny R., Heer F.T., de Beer T.A.P., Rempfer C., Bordoli L. (2018). SWISS-MODEL: Homology modelling of protein structures and complexes. Nucleic Acids Res..

[56] Abraham M.J., Murtola T., Schulz R., Páll S., Smith J.C., Hess B., Lindahl E. (2015). GROMACS: High performance molecular simulations through multi-level parallelism from laptops to supercomputers. Softw. X.

[57] Labute P. (2009). Protonate3D: Assignment of ionization states and hydrogen coordinates to macromolecular structures. Proteins.

[58] O’Boyle N.M., Banck M., James C.A., Morley C., Vandermeersch T., Hutchison G.R. (2011). Open Babel: An open chemical toolbox. J. Cheminform..

[59] Huang J., Rauscher S., Nawrocki G., Ran T., Feig M., de Groot B.L., Grubmüller H., MacKerell A.D. (2017). CHARMM36m: An improved force field for folded and intrinsically disordered proteins. Nat. Methods.

[60] Vanommeslaeghe K., Hatcher E., Acharya C., Kundu S., Zhong S., Shim J., Darian E., Guvench O., Lopes P., Vorobyov I. (2010). CHARMM General Force Field: A force field for drug-like molecules compatible with the CHARMM all-atom additive biological force fields. J. Comput. Chem..

[61] Alcolea P.J., Alonso A., Molina R., Jiménez M., Myler P.J., Larraga V. (2019). Functional genomics in sand fly-derived *Leishmania* promastigotes. PLoS Negl. Trop. Dis..

[62] González U., Pinart M., Sinclair D., Firooz A., Enk C., Vélez I.D., Esterhuizen T.M., Tristan M., Alvar J. (2015). Vector and reservoir control for preventing leishmaniasis. Cochrane Database Syst. Rev..

[63] Okwu D.G., Manego R.Z., Duparc S., Kremsner P.G., Ramharter M., Mombo-Ngoma G. (2025). The non-artemisinin antimalarial drugs under development: A review. Clin. Microbiol. Infect..

[64] Lenharo M. (2025). First new type of malaria treatment in decades shows promise against drug resistance. Nature.

[65] Sazdova I., Keremidarska Markova M., Dimitrova D., Mitrokhin V., Kamkin A., Hadzi Petrushev N., Bogdanov J., Schubert R., Gagov H., Avtanski D. (2023). Anticarcinogenic potency of EF24: An overview of its pharmacokinetics, efficacy, mechanism of action, and nanoformulation for drug delivery. Cancers.

[66] Ceja-Galicia Z.A., Aranda-Rivera A.K., Amador-Martínez I., Aparicio-Trejo O.E., Tapia E., Trujillo J., Ramírez V., Pedraza-Chaverri J. (2023). The development of dyslipidemia in chronic kidney disease and associated cardiovascular damage, and the protective effects of curcuminoids. Foods.

[67] Nguyen X.-H., Yoo J. (2025). Current status and future prospects of toxicity assessment using organoids. Toxicol. Res..

[68] Cerritelli S.M., Crouch R.J. (2008). Ribonuclease H: The enzymes in eukaryotes. FEBS J..

[69] Damasceno J.D., Briggs E.M., Krasilnikova M., Marques C.A., Lapsley C., McCulloch R. (2025). R-loops acted on by RNase H1 influence DNA replication timing and genome stability in *Leishmania*. Nat. Commun..

[70] Misra S., Bennett J., Friew Y.N., Abdulghani J., Irvin-Wilson C.V., Tripathi M.K., Williams S., Chaudhuri M., Chaudhuri G. (2005). A type II ribonuclease H from *Leishmania* mitochondria: An enzyme essential for the growth of the parasite. Mol. Biochem. Parasitol..

[71] Mishra M., Bennett J.R., Chaudhuri G. (2001). Increased efficacy of antileishmanial antisense phosphorothioate oligonucleotides in *Leishmania amazonensis* overexpressing ribonuclease H. Biochem. Pharmacol..

[72] Musiyenko A., Majumdar T., Andrews J., Adams B., Barik S. (2012). PRMT1 methylates the single Argonaute of *Toxoplasma gondii* and is important for the recruitment of Tudor nuclease for target RNA cleavage by antisense guide RNA. Cell. Microbiol..

[73] Rabelo V.W.-H., Carneiro L.S.d.A., Martins L.L.B., Almeida-Souza F., Silva L.S., Amorim L.d.S.C., Sanchez-Nuñez M.L., Calabrese K.d.S., Abreu P.A., Buarque C.D. (2024). Biological evaluation of 3-aryl and/or 4-(*N*-aryl)aminocoumarins against human pathogens: Antileishmanial and antiviral activities. Future Pharmacol..

[74] Potvin J.-É., Fani F., Queffeulou M., Gazanion É., Leprohon P., Ouellette M. (2023). Increased copy number of the target gene squalene monooxygenase as the main resistance mechanism to terbinafine in *Leishmania infantum*. Int. J. Parasitol. Drugs Drug Resist..

