# Supplementary material for: In Vitro and In Silico Evaluation of the Anti-Infective Potential of EF24-Analogous Curcuminoids: Antiprotozoal Activity and HIV-1 Ribonuclease H Inhibition
Source: Pathogens. 2026 Aug 20;15(8):876. doi: 10.3390/pathogens15080876 (PMC13516635; doi:10.3390/pathogens15080876)
Supplement: Supplementary file 1 [file pathogens-15-00876-s001.zip › pathogens-4470538-supplementary.pdf]

Supplementary Materials

Table S1. *Toxoplasma gondii* protein targets and a structurally relevant mammalian comparator selected for molecular docking. The table summarizes the protein identity, Protein Data Bank (PDB) accession, source organism, deposited PDB construct sequence, construct length, and principal biological function.

#	Target protein	PDB ID	Organism	PDB construct sequence (one-letter code)	Construct length (aa)	Biological function
**1.**	Hypoxanthine-guanine phosphoribosyl transferase (HGPRT)	1FSG	*T. gondii* RH	GSHMASKPIEDYGKGKGRIEPMYI PDNTFYNADDFLVPPHCKPYIDKI LLPGGLVKDRVEKLAYDIHRTYFG EELHIICILKGSRGFFNLLIDYLATI QKYSGRESSVPPFFEHYVRLKSYQ NDNSTGQLTVLSDDLSIFRDKHVL IVEDIVDTGFTLTEFGERLKAVGPK SMRIATLVEKRTDRSNSLKGDFVG FSIEDVWIVGCCYDFNEMFRDFD HVAVLSDAARKKFEK	233	Purine salvage (conversion of hypoxanthine and guanine to IMP and GMP, respectively) [1]
**2.**	Bradyzoite-specific L-lactate dehydrogenase 2 (LDH2)	1SOV	*T. gondii*	MTGTVSRRKKIAMIGSGMIGGTM GYLCVLRELADVVLFDVVTGMPE GKALDDSQATSIADTNVSVTSAN QYEKIAGSDVVIITAGLTKVPGKS DKEWSRNDLLPFNAKIIREVAQG VKKYCPLAFVIVVTNPLDCMVKC FHEASGLPKNMVCGMANVLDSA RFRRFIADQLEISPRDIQATVIGTH GDHMLPLARYVTVNGFPLREFIK KGKMTEAKLAEIVERTKKAGGEI VRLLGQGSAYYAPALSAITMAQA FLKDEKRVLPCSVYCQGEYGLHD MFIGLPAVIGGGGIEQVIELELTHE EQECFRKSVDDVVELNKSLAALG PG	328	Bradyzoite energy metabolism (pyruvate to lactate) [2]

#	Target protein	PDB ID	Organism	PDB construct sequence (one-letter code)	Construct length (aa)	Biological function
**3.**	Protein phosphatase 2C (PP2C)	3D8K	*T. gondii*	MSLRHLYIEEGRTVCASATSRNRRPTS ESPHSDDVVVVEGMLRGRPETRVHA MFDGFQGRHSAMWLAQNVMNYLN DLRDVNEEEITRQFERMDGDLRAAN LPGGSSALIIFVRYEKKPTEARVVGRQI VPEGAKEFTSVAEALGGPLMPVVAM NFRRDPRAAKGIYTIHVASLGNSRCV LKSGRTAIHLSTPHTASSHKERHRVQ AAGGVFTTVNGELLLGGVVPMTRAF GSFDFKKGGQGKLQQDLVSAVPDVT TFFAYPGDDIVAGTAGAFAHFRSHA AIAAAIALYPVSPETVLDAAKAMVV NAKRRKVTKNISTFVRHLPESRTRSQ KMLEGTSGENGEEDFSIDRTNELTQA LQAGFFSFLLYYEGHHHHHH	377	Protein dephosphory-lation and parasite growth regulation [3]
**4.**	Pyruvate kinase 1 (PK1)	3EOE	*T. gondii*	MHHHHHHSSGRENLYFQGIRMS QILEPRSEEDWTAHRTRIVCTMGP ACWNVDTLVKMIDAGMNVCRL NFSHGDHETHARTVQNIQEAMK QRPEARLAILLDTKGPEIRTGFLKD HKPITLQQGATLKIVTDYNLIGDE TTIACSYGALPQSVKPGNTILIADG SLSVKVVEVGSDYVITQAQNTATI GERKNMNLPNVKVQLPVIGEKD KHDILNFGIPMGCNFIAASFVQSA DDVRYIRGLLGPRGRHIRIIPKIEN VEGLVNFDEILAEADGIMIARGDL GMEIPPEKVFLAQKMMIAKCNVV GKPVITATQMLESMIKNPRPTRAE AADVANAVLDGTDCVMLSGETA NGEFPVITVETMARICYEAETCVD YPALYRAMCLAVPPPISTQEAVAR AAVETAECVNAAIILALTETGQTA RLIAKYRPMQPILALSASESTIKHL QVIRGVTTMQVPSFQGTDHVIRN AIVVAKERELVTEGESIVAVHGMK EEVAGSSNLLKVLTVE	511	Glycolysis and ATP production (phosphoenol-pyruvate to pyruvate) [4]

#	Target protein	PDB ID	Organism	PDB construct sequence (one-letter code)	Construct length (aa)	Biological function
**5.**	Bradyzoite-specific enolase 1 (ENO1)	3OTR	*T. gondii*	MVVIKDIVAREILDSRGNPTIEVDVSTEGGVFRAAVPSGASTGIYEALELRDKDPKRYLGKGVLNAVEIVRQEIKPALLGKDPCDQKGIDMLMVEQLDGTKNEWGYSKSKLGANAILGVSIACCRAGAASKGLPLYKYIATLAGKTIDKMVMPVPFFNVINGGEHAGNGLALQEFLIAPVGAPNIREAIRYGSETYHHLKNVIKNKYGLDATNVGDEGGFAPNVATAEEALNLLVEAIKAAGYEGKIKIAFDAAASEFYKQDEKKYDLDYKCKTKNASKHLTGEKLKEVYEGWLKKYPIISVEDPFDQDDFASFSAFTKDVGEKTQVIGDDILVTNILRIEKALKDKACNCLLLKVNQIGSVTEAIEACLLAQKSGWGVQVSHRSGETEDSFIADLVVGLRCGQIKSGSPCRSERLCKYNQLMRIEESLGADCVYAGESFRHPKRSHHHHHH	452	Bradyzoite glycolysis and stage-specific gene regulation [5]
**6.**	ROP5B pseudokinase domain	3Q60	*T. gondii*	GSHMEAGDSFMRDLLKREEELIGYCREEALKEPAAMVEAVTATVWPQNAETTVDSLLSQGERKLKLVEPLRVGDRSVVFLVRDVERLEDFALKVFTMGAENSRSELERLHEATFAAARLLGESPEEARDRRRLLLPSDAVAVQSQPPFAQLSPGQDDYAVANYLLLMPAASVDLELLFSTLDFVYVFRGDEGILALHILTAQLIRLAANLQSKGLVHGHFTPDNLFIMPDGRLMLGDVSALWKVGTRGPASSVPVTYAPREFLNASTATFTHALNAWQLGLSIYRVWCLFLPFGLVTPGIKGSWKRPSLRVPGTDSLAFGSCTPLPDFVKTLIGRFLNFDRRRRLLPLEAMETPEFLQLQNEISSSLSTGQ	371	Virulence and evasion of host immunity-related GTPases (IRGs) [6]

#	Target protein	PDB ID	Organism	PDB construct sequence (one-letter code)	Construct length (aa)	Biological function
**7.**	Fructose-bisphosphate aldolase 1 (TgALD1)	4D2J	*T. gondii*	GSHMAMGPISQEVAKELAENARKIAAPGKGILAADESTGTIKKRFDSIGVENTEANRAFYRDLLFSTKGLGQYISGAILFEETLYQKSPSGVPMVDLLKAEGIIPGIKVDKGLETLPLTDDEKATMGLDGLSERCKKYYEAGARFAKWRAVLSIDPAKGKPTNLSITEVAHGLARYAAICQANRLVPIVEPEILTDGSHDITVCAEVTERVLAAVFKALNDHHVLLEGALLKPNMVTHGSDCPKPASHEEIAFYTVRSLKRTVPPALPGVMFLSGGQSEEDASLNLNEMNKMGPHPFQLSFSYGRALQASCLKAWKGVPENKAKAQQVLMERARANGEAQLGKYGGGAGGAAA	353	Glycolysis and parasite energy metabolism [7]
**8.**	Macrophage migration inhibitory factor (TgMIF)	4DH4	*T. gondii*	PKCMIFCPVAATPAQQDALLKDAEKAVADALGKPLSYVMVGYSQTGQMRFGGSSDPCAFIRVASIGGITSSTNCKIAAALSAACERHLGVPKNRIYTTFTNKSPSEWAMGDRTF	114	Host immune modulation and parasite survival (tautomerase activity) [8]
**9.**	ROP18 kinase domain	4JRN	*T. gondii*	GAHMSELVFEKADSGCVIGKRILAHMQEQIGQPQALENSERLDRILTVAAWPPDVPKRFVSVTTGETRTLVRGAPLGSGGFATVYEATDVETNEELAVKVFMSEKEPTDETMLDLQRESSCYRNFSLAKTAKDAQESCRFMVPSDVVMLEGQPASTEVVIGLTTRWVPNYFLLMMRAEADMSKVISWVFGDASVNKSEFGLVVRMYLSSQAIKLVANVQAQGIVHTDIKPANFLLLKDGRLFLGDFGTYRINNSVGRAIGTPGYEPPERPFQATGITYTFPTDAWQLGITLYCIWCKERPTPADGIWDYLHFADCPSTPELVQDLIRSLLNRDPQKRMLPLQALETAAFKEMDSVVKGAAQNFEQQEHLHTE	372	Virulence and host IRG inactivation by phosphorylation [9]

#	Target protein	PDB ID	Organism	PDB construct sequence (one-letter code)	Construct length (aa)	Biological function
**10.**	ROP5C pseudokinase domain	4LV8	*T. gondii* type I	GSHMEAGDSFMRDLLKREEELIGYCREEALKEPAAMVEAVTATVWPQNAETTVDSLLSQGERKLKLVEPLRVGDRSVVFLVRDVERLEDFALKVFTMGAENSRSELERLHEATFAAARLLGESPEEARDRRRLLLPSDAVAVQSQPPFAQLSPGQSDYAVANYLLLMPAASVDLELLFRTLDFLYVLRSTEDFLALHILTAQLIRLAANLQSKGLVHGHFTPDNLFIMPDGRLMLGDVSVLRKVGTRGPASSVPVTYAPREFLNASTATFTHALDAWQLGLSIYRVWCLFLPFGLVTPGIKGSWKRPSLRVPGTDSLAFGSCTPLPDFVQTLIGRFLNFDRRRRLLPLEAMETPEFLQLQNEISSSLSTGQ	371	Virulence and host IRG inactivation through IRGa6 binding (pseudokinase GDI) [10]
**11.**	Calcium-dependent protein kinase 1 (TgCDPK1)	4M84	*T. gondii*	GPGSMMDHLHATPGMFVQHSTAIFSDRYKGQRVLGKGSFGEVILCKDKITGQECAVKVISKRQVKQKTDKESLLREVQLLKQLDHPNIMKLYEFFEDKGYFYLVGEVYTGGELFDEIISRKRFSEVDAARIIRQVLSGITYMHKNKIVHRDLKPENLLLESKSKDANIRIIDFGLSTHFEASKKMKDKIGTAYYIAPEVLHGTYDEKCDVWSTGVILYILLSGCPPFNGANEYDILKKVEKGKYTFELPQWKKVSESAKDLIRKMLTYVPSMRISARDALDHEWIQTYTKEQISVDVPSLDNAILNIRQFQGTQKLAQAALLYMGSKLTSQDETKELTAIFHKMDKNGDGQLDRAELIEGYKELMRMKGQDASMLDASAVEHEVDQVLDAVDFDKNGYIEYSEFVTVAMDRKTLLSRERLERAFRMFDSDNSGKISSTELATIFGVSDVDSETWKSVLSEVDKNNDGEVDFDEFQQMLLKLCGN	484	Calcium-dependent microneme secretion, motility, host-cell invasion, and egress [11]

#	Target protein	PDB ID	Organism	PDB construct sequence (one-letter code)	Construct length (aa)	Biological function
**12.**	Enoyl-acyl carrier protein reductase (ENR)	4O1M	*T. gondii*	SAFPIDLRGQTAFVAGVADSHGYGWAIAKHLASAGARVALGTWPPVLGLFQKSLQSGRLDEDRKLPDGSLIEFAGVYPLDAAFDKPEDVPQDIKDNKRYAGVDGYTIKEVAVKVKQDLGNIDILVHSLANGPEVTKPLLETSRKGYLAASSNSAYSFVSLLQHFGPIMNEGGSAVTLSYLAAERVVPGYGGGMSSAKAALESDTRTLAWEAGQKYGVRVNAISAGPLKSRAASAIGKSGEKSFIDYAIDYSYNNAPLRRDLHSDDVGGAALFLLSPLARAVSGVTLYVDNGLHAMGQAVDSRSMPPLQRATQEIN	315	Apicoplast type II fatty-acid biosynthesis (enoyl-ACP reduction) [12]
**13.**	Micronemal protein 2 N-terminal fragment (MIC2; VWA–TSR1 engineered construct)	4OKR	*T. gondii*	AAEGCTNQLDICFLIDSSGSIGIQNFRLVKQFLHTFLMVLPIGPEEVNNAVVTYSTDVHLQWDLQSPNAVDKQLAAHAVLEMPYKKGSTNTADGLKACKQILFTGSRPGREHVPKLVIGMTDGESDSDFRTVRAAKEIRELGGIVTVLAVGHYVKHSECRSMCGCSGTSDDDSPCPLYLRADWGQLATAIKPMLKEVCKTLPQDAICSDWSAWSPCSVSCGDGSQIRTRTEVSAPQPGTPTCPDCPAPMGRTCVEQGGLEEIRECSAGVCAHHHHHHA	278	Host-cell adhesion, gliding motility, and invasion [13]
**14.**	Protein kinase A (PKA) regulatory cAMP-binding domain	5J3U	*T. gondii*	VYEKDEGQKEQLERILRQSFLFNSLDEKDLNTVILAMQEKKIEASTCLIREGDDGECLYIVQSGELNCSKLIDGEERVVKVVGPGDAFGELALLYNAPRAATVTSVSACDLWELGRDTFNAIVKDAATKRRSMYDSFLKSVHILDGMDAYERGKVADALRTEMFTDGAYIVRQGELGDVFYIVEEGSAVATKSFGPGQPPIEVKKYQAGDYFGELALINEEPRAANVIAHGICKVACLERKSFKRLMGSVQDLLSKKASEY	261	cAMP-dependent regulation of calcium signaling, parasite growth, and egress [14]

#	Target protein	PDB ID	Organism	PDB construct sequence (one-letter code)	Construct length (aa)	Biological function
**15.**	Mitochondrial cytochrome bc_1 complex (mammalian comparator)	6XVF	*Bos taurus*	TNIRKSHPLMKIVNNAFIDLPAPS<br>NISSWWNFGSLLGICLILQILTGLF<br>LAMHYTSDTTTAFSSVTHICRDVN<br>YGWIIRYMHANGASMFFICLYMH<br>VGRGLYYGSYTFLETWNIGVILLL<br>TVMATAFMGYVLPWGQMSFWG<br>ATVITNLLSAIPYIGTNLVEWIWG<br>GFSVDKATLTRFFAFHFILPFIIMAI<br>AMVHLLFLHETGSNNPTGISSDV<br>DKIPFHPYYTIKDILGALLLILALM<br>LLVLFAPDLLGDPDNYTPANPLN<br>TPPHIKPEWYFLFAYAILRSIPNKL<br>GGVLALAFSILILALIPLLHTSKQRS<br>MMFRPLSQCLFWALVADLLTLTW<br>IGGQPVEHPYITIGQLASVLYFLLIL<br>VLMPTAGTIENKLLKW	378	Mitochondrial electron transport and ATP production (cytochrome bc_1 complex) [15]
**16.**	Prolyl-tRNA synthetase (ProRS)	7F9T	*T. gondii*	GAMVTAKKDENFSEWYTQAIVRS<br>EMIEYYDISGCYIMRPWAFHIWEK<br>VQRFFDDEIKKMGVENSYFPMFVS<br>RHKLEKEKDHVEGFSPEVAWVTH<br>YGDSPLPEKIAIRPTSETIMYPAYA<br>KWIRSHRDLPLKLNQWCSVVRW<br>EFKQPTPFLRTREFLWQEGHTAH<br>ATEEEAWELVLDILELYRRWYEEC<br>LAVPVIKGEKSEGEKFAGGKKTTT<br>VEAFIPENGRGIQAATSHLLGTNF<br>AKMFEIEFEDEEGHKRLVHQTSW<br>GCTTRSLGVMIMTHGDDKGLVIP<br>PRVASVQVVIIPILFKDENTGEILG<br>KCRELKTMLEKADIRVRIDDRSNY<br>TPGWKYNHWEVKGVPLRLELGP<br>KDLAKGTARVVRRDTGEAYQISW<br>ADLAPKLLELMEGIQRSLFEKAKA<br>RLHEGIEKISTFDEVMPALNRKHL<br>VLAPWCEDPESEEQIKKETQKLSEI<br>QAIEAGDSEQVMTGAMKTLCIPF<br>DQPPMPEGTKCFYTGKPAKRWTL<br>WGRSY	500	Protein translation through prolyl-tRNA charging [16]

#	Target protein	PDB ID	Organism	PDB construct sequence (one-letter code)	Construct length (aa)	Biological function
**17.**	Bifunctional dihydrofolate reductase–thymidylate synthase (TgDHFR-TS)	7FGX	*T. gondii*	MQKPVCLVVAMTPKRGIGINNGLPWPHLTTDFKHFSRVTKTTPEEASRLNGWLPRKFAKTGDSGLPSPSVGKRFNAVVMGRKTWESMPRKFRPLVDRLNIVVSSSLKEEDIAAEKPQAEGQQRVRVCASLPAALSLLEEEYKDSVDQIFVVGGAGLYEAALSLGVASHLYITRVAREFPCDVFFPAFPGDDILSNKSTAAQAAAPAESVFVPFCPELGREKDNEATYRPIFISKTFSDNGVPYDFVVLEKRRKTDDAATAEPSNAMSSLTSTRETTPVHGLQAPSSAAAIAPVLAWMDEEDRKKREQKELIRAVPHVHFRGHEEFQYLDLIADIINNGRTMDDRTGVGVISKFGCTMRYSLDQAFPLLTTKRVFWKGVLEELLWFIRGDTNANHLSEKGVKIWDKNVTREFLDSRNLPHREVGDIGPGYGFQWRHFGAAYKDMHTDYTGQGVDQLKNVIQMLRTNPTDRRMLMTAWNPAALDEMALPPCHLLCQFYVNDQKELSCIMYQRSCDVGLGVPFNIASYSLLTLMVAHVCNLKPKEFIHFMGNTHVYTNHVEALKEQLRREPRPFPIVNILNKERIKEIDDFTAEDFEVVGYVPHGRIQMEMAV	610	Folate metabolism and DNA synthesis through tetrahydrofolate regeneration and *de novo* dTMP production [17]

#	Target protein	PDB ID	Organism	PDB construct sequence (one-letter code)	Construct length (aa)	Biological function
**18.**	Cystathionine γ-lyase N360S mutant (TgCGL-N360S)	8BIX	*T. gondii*	MASKQNDKDGAVRRDASFECGV<br>KAGDWLPGFTPREETVYVHGGVE<br>PDPLTGAILPPIYQNTTFVQESVEN<br>YLSKGFSYSRTSNPTVLSLEKKIAEI<br>EGGFGACCFATGMAATVTIFSAFL<br>APGDHCLVTNCSYGGTNRCARL<br>HFSKYNIDFEFIDFRDPTNVEKAIR<br>PQTKVVFSESPCNPTLYLADIEAIS<br>QICKEKKVLHVCDSTFATPYMMR<br>PLDLGADIVVQSTTKYYDGHNCT<br>LGGAVISSTKEIHDKVFFLRNVMG<br>NIMSAQTAFYTLLTLKTLPIRVEK<br>QSANAQKIAEFLSKHHKVEHVIYP<br>GIPSFPQKELALKQHKNVHGGML<br>AFEVKGGTEAGIRMMNHVPRPW<br>SLCESLGACESIITCPAVFTHANML<br>REDRLKVGITDGFIRVSVGIEDVN<br>DLIDGLDYALSKA	417	Cysteine biosynthesis via reverse trans-sulfuration, with secondary hydrogen sulfide production [18]
**19.**	Actin 1 (TgACT1)	8TRM	*T. gondii*	MADEEVQALVVDNGSGNVKAG<br>VAGDDAPRAVFPSIVGKPKNPGI<br>MVGMEEKDCYVGDEAQSKRGILT<br>LKYPIEHGIVTNWDDMEKIWHHT<br>FYNELRVAPEEHPVLLTEAPLNPK<br>ANRERMTQIMFETFNVPAMYVAI<br>QAVLSLYSSGRTTGIVLDSGDGVS<br>HTVPIYEGYALPHAIMRLDLAGR<br>DLTEYMMKILHERGYGFTTSAEKE<br>IVRDIKEKLCYIALDFDEEMKAAE<br>DSSDIEKSYELPDGNIITVGNERFR<br>CPEALFQPSFLGKEAAGVHRTTFD<br>SIMKCDVDIRKDLYGNVVLSGGTT<br>MYEGIGERLTKELTSLAPSTMKIK<br>VVAPPERKYSVWIGGSILSSLSTFQ<br>QMWITKEEYDESGPSIVHRKCF	376	Actin-dependent gliding motility, host-cell invasion, egress, and intracellular cargo transport [19]

#	Target protein	PDB ID	Organism	PDB construct sequence (one-letter code)	Construct length (aa)	Biological function
**20.**	Peptidyl-prolyl cis–trans isomerase (TgCyp23)	8R7U	*T. gondii*	GHMAPAPPANSGESSLLSESELPA GISYAEAMEGGSRPLLHPDNPVVF FDISIGSHEAGRIKIELFKNLAPKSA ENFRQFCTGEFRQNQVPIGYKGA TFHRIIKNFMIQGGDFVKGDGTGR LSIYGSSFPDEAFVLPHFRSGLLSLA NSGPDTNGCQFFITCAKCDWLNR KHVVFGQVLGKESMQVVRKIEHV TVDGGNRPRIPVTVTQCGEL	213	Cyclophilin-mediated protein folding through peptidyl-prolyl cis–trans isomerization [20]

References (Table S1)

1. Héroux, A.; White, E.L.; Ross, L.J.; Kuzin, A.P.; Borhani, D.W. Substrate deformation in a hypoxanthine–guanine phosphoribosyltransferase ternary complex: The structural basis for catalysis. *Structure* **2000**, *8*, 1309–1318. doi:10.1016/S0969-2126(00)00546-3.
2. Kavanagh, K.L.; Wilson, D.K. *Toxoplasma gondii* bradyzoite-specific lactate dehydrogenase 2 (LDH2), apo form. RCSB Protein Data Bank 2005, PDB ID: 1SOV. doi:10.2210/pdb1SOV/pdb.
3. Damodharan, L.; Sauder, J.M.; Burley, S.K.; Swaminathan, S. Crystal structure of a phosphatase from *Toxoplasma gondii*. RCSB Protein Data Bank 2008, PDB ID: 3D8K. doi:10.2210/pdb3D8K/pdb.
4. Bakszt, R.; Wernimont, A.; Allali-Hassani, A.; Mok, M.W.; Hills, T.; Hui, R.; Pizarro, J.C. The crystal structure of *Toxoplasma gondii* pyruvate kinase 1. *PLoS ONE* **2010**, *5*, e12736. doi:10.1371/journal.pone.0012736.
5. Ruan, J.; Mouveaux, T.; Light, S.H.; Minasov, G.; Anderson, W.F.; Tomavo, S.; Ngô, H.M. The structure of bradyzoite-specific enolase from *Toxoplasma gondii* reveals insights into its dual cytoplasmic and nuclear functions. *Acta Crystallogr. D Biol. Crystallogr.* **2015**, *71*, 417–426. doi:10.1107/S1399004714026479.
6. Reese, M.L.; Boothroyd, J.C. A conserved non-canonical motif in the pseudoactive site of the ROP5 pseudokinase domain mediates its effect on *Toxoplasma* virulence. *J. Biol. Chem.* **2011**, *286*, 29366–29375. doi:10.1074/jbc.M111.253435.
7. Tonkin, M.L.; Halavaty, A.S.; Ramaswamy, R.; Ruan, J.; Igarashi, M.; Ngô, H.M.; Boulanger, M.J. Structural and functional divergence of the aldolase fold in *Toxoplasma gondii*. *J. Mol. Biol.* **2015**, *427*, 840–852. doi:10.1016/j.jmb.2014.09.019.
8. Sommerville, C.; Richardson, J.M.; Williams, R.A.M.; Mottram, J.C.; Roberts, C.W.; Alexander, J.; Henriquez, F.L. Biochemical and immunological characterization of *Toxoplasma gondii* macrophage migration inhibitory factor. *J. Biol. Chem.* **2013**, *288*, 12733–12741. doi:10.1074/jbc.M112.419911.
9. Lim, D.; Gold, D.A.; Julien, L.; Rosowski, E.E.; Niedelman, W.; Yaffe, M.B.; Saeij, J.P.J. Structure of the *Toxoplasma gondii* ROP18 kinase domain reveals a second ligand-binding pocket required for acute virulence. *J. Biol. Chem.* **2013**, *288*, 34968–34980. doi:10.1074/jbc.M113.523266.
10. Reese, M.L.; Shah, N.; Boothroyd, J.C. The *Toxoplasma* pseudokinase ROP5 is an allosteric inhibitor of the immunity-related GTPases. *J. Biol. Chem.* **2014**, *289*, 27849–27858. doi:10.1074/jbc.M114.567057.
11. Zhang, Z.; Ojo, K.K.; Vidadala, R.; Huang, W.; Geiger, J.A.; Scheele, S.; Choi, R.; Reid, M.C.; Keyloun, K.R.; Rivas, K.; Siddaramaiah, L.K.; Comess, K.M.; Robinson, K.P.; Merta, P.J.; Kifle, L.; Hol, W.G.J.; Parsons, M.; Merritt, E.A.; Maly, D.J.; Verlinde, C.L.M.J.; Van Voorhis, W.C.; Fan, E. Potent and selective inhibitors of CDPK1 from *T. gondii* and *C. parvum* based on a 5-aminopyrazole-4-carboxamide scaffold. *ACS Med. Chem. Lett.* **2014**, *5*, 40–44. doi:10.1021/ml400315s.
12. Wilkinson, C.; McPhillie, M.J.; Zhou, Y.; Woods, S.; Afanador, G.A.; Rawson, S.; Khaliq, F.; Prigge, S.T.; Roberts, C.W.; Rice, D.W.; McLeod, R.; Fishwick, C.W.; Muench, S.P. The benzimidazole-based drugs show good activity against *T. gondii* but poor activity against its proposed enoyl reductase enzyme target. *Bioorg. Med. Chem. Lett.* **2014**, *24*, 911–916. doi:10.1016/j.bmcl.2013.12.066.

13. Song, G.; Springer, T.A. Structures of the *Toxoplasma gliding* motility adhesin. *Proc. Natl. Acad. Sci. USA* **2014**, *111*, 4862–4867. doi:10.1073/pnas.1403059111.
14. El Bakkouri, M.; Walker, J.R.; Tempel, W.; Loppnau, P.; Gräslund, S.; Arrowsmith, C.H.; Edwards, A.M.; Bountra, C.; Hui, R.; Lin, L. Co-crystal structure of the regulatory domain of *Toxoplasma gondii* protein kinase A with cAMP. RCSB Protein Data Bank 2016, PDB ID: 5J3U. doi:10.2210/pdb5J3U/pdb.
15. McPhillie, M.J.; Zhou, Y.; Hickman, M.R.; Gordon, J.A.; Weber, C.R.; Li, Q.; Lee, P.J.; Amporndanai, K.; Johnson, R.M.; Darby, H.; Woods, S.; Li, Z.; Priestley, R.S.; Ristroph, K.D.; Biering, S.B.; El Bissati, K.; Hwang, S.; Hakim, F.E.; Dovgin, S.M.; Lykins, J.D.; Roberts, L.; Hargrave, K.; Cong, H.; Sinai, A.P.; Muench, S.P.; Dubey, J.P.; Prud'homme, R.K.; Lorenzi, H.A.; Biagini, G.A.; Moreno, S.N.; Roberts, C.W.; Antonyuk, S.V.; Fishwick, C.W.G.; McLeod, R. Potent tetrahydroquinolone eliminates apicomplexan parasites. *Front. Cell. Infect. Microbiol.* **2020**, *10*, 203. doi:10.3389/fcimb.2020.00203.
16. Malhotra, N.; Manickam, Y.; Sharma, A. *Toxoplasma gondii* prolyl-tRNA synthetase in complex with inhibitor L97 and halofuginone. RCSB Protein Data Bank 2022, PDB ID: 7F9T. doi:10.2210/pdb7F9T/pdb.
17. Vanichtanankul, J.; Yoomuang, A.; Taweechai, S.; Saeyang, T.; Pengon, J.; Yuvaniyama, J.; Tarnchompoo, B.; Yuthavong, Y.; Kamchonwongpaisan, S. Structural insight into effective inhibitors' binding to *Toxoplasma gondii* dihydrofolate reductase thymidylate synthase. *ACS Chem. Biol.* **2022**, *17*, 1691–1702. doi:10.1021/acschembio.1c00627.
18. Fernández-Rodríguez, C.; Conter, C.; Oyenarte, I.; Favretto, F.; Quintana, I.; Martinez-Chantar, M.L.; Astegno, A.; Martínez-Cruz, L.A. Structural basis of the inhibition of cystathionine γ-lyase from *Toxoplasma gondii* by propargylglycine and cysteine. *Protein Sci.* **2023**, *32*, e4619. doi:10.1002/pro.4619.
19. Hvorecny, K.L.; Sladewski, T.E.; De La Cruz, E.M.; Kollman, J.M.; Heaslip, A.T. *Toxoplasma gondii* actin filaments are tuned for rapid disassembly and turnover. *Nat. Commun.* **2024**, *15*, 1840. doi:10.1038/s41467-024-46111-3.
20. Favretto, F.; Jiménez-Faraco, E.; Catucci, G.; Di Matteo, A.; Travaglini-Allocatelli, C.; Sadeghi, S.J.; Dominici, P.; Hermoso, J.A.; Astegno, A. Evaluating the potential of non-immunosuppressive cyclosporin analogs for targeting *Toxoplasma gondii* cyclophilin: Insights from structural studies. *Protein Sci.* **2024**, *33*, e5157. doi:10.1002/pro.5157.

Table S2. *Leishmania* protein targets selected for molecular docking. The table summarizes the protein identity, experimental Protein Data Bank (PDB) entry or homology-model source, source organism, amino-acid sequence used for docking, structure/model length, and principal biological function.

#	Target protein	Structure source (PDB ID or homology model)	Organism	Amino-acid sequence used for docking (one-letter code)	Structure/ model length (aa)	Biological function
**1.**	Trypanothione synthetase-amidase	2VOB	*L. major*	MSSLQRASVSFNKPGHIPFGAVQGYAPGGVPAYSNKHDHYFSGERNIEDNIFFGFKYQCVEFARRWLLVRKGLLLPDVNWACHIFQLKEVRDAATTESFAVLQVRNGTTTKPEADALLVYPSTDANPVGHVGTITEVGDDYVCVADQNYRFHKWESSCAYKLKLDHRDGIWTIIDDIDADEIEIPLGWLTFPGRANRPEGAPPVALHPSLHFKEPPKPYLLRRNFLPTESKANWLDMNNPAERLFVEEFGMDVSRTRLEEKVVSYYESNHEFHLRCVAYGTQLHAIFMEATAQVIESDEKLRLFAIPEEFWPRIRHSWKYQQTYISGRFDFAFNNETGEVKCFEYNADSASTLLECGLIQQKWAESVGLDKQDTRGSGFAVERNLKMAWANSGATGRVHFCVDEEREEQYTALYCMQAAEAVGLEGKLCILFDEFRFDDNGHVVDSDGVRVRNVWKTWMWESAITDYYAAREERGENWKPSPKDKVRLCDLLLGDDWEILYFEPMWKVIPSNKAILPMIYHNHPEHPAILKAEYELTDELRKHGYAKKPIVGRVGSNVIITSGDGVVHAESGGKYGKRNMIYQQLFELKKQDDYYAIIGGWMIGDAFSGTGIREDKSVITGVDSPFAAVRIKTDKLPHPVTLKDIDKMAEDE	652	Trypanothione biosynthesis and hydrolysis, thiol-redox homeostasis, and oxidative-stress defense [1]

#	Target protein	Structure source (PDB ID or homology model)	Organism	Amino-acid sequence used for docking (one-letter code)	Structure/ model length (aa)	Biological function
**2.**	Sterol 14α-demethylase (CYP51)	3L4D	*L. infantum*	KGKLPPVVHGTTPFVGHIIQFG<br>KDPLGFMLKAKKKYGGIFTMN<br>ICGNRITVVGDVHQHSKFFTPR<br>NEILSPREVYSFMVPVFGEGVA<br>YAAPYPRMREQLNFLAEELTV<br>AKFQNFAPSIQHEVRKFMKAN<br>WNKDEGEINILDDCSAMIINTA<br>CQCLFGEDLRKRLDARQFAQL<br>LAKMESCLIPAAVFLPWILKLPL<br>PQSYRCRDARAELQDILSEIIIAR<br>EKEEAQKDTNTSDLLAGLLGA<br>VYRDGTRMSQHEVCGMIVAA<br>MFAGQHTSTITTTWSLLHLMD<br>PRNKRHLAKLHQEIDEFPAQL<br>NYDNVMEEMPFAEQCARESIR<br>RDPPLVMLMRKVLKPVQVGKY<br>VVPEGDIIACSPLLSHQDEEAFP<br>NPREWNPERNMKLVDGAFCG<br>FGAGVHKCIGEKFGLLQVKTVL<br>ATVLRDYDFELLGPLPEPNYHT<br>MVVGPTASQCRVKYIKKKKAA<br>A	453	Sterol biosynthesis through 14α-demethylation and membrane biogenesis [2]
**3.**	N-myristoyl-transferase (NMT)	4CGO	*L. major*	AHAFWSTQPVPQTEDETEKIVF<br>AGPMDEPKTVADIPEEPYPIAST<br>FEWWTPNMEAADDIHAIYELL<br>RDNYVEDDDSMFRFNYSEEFL<br>QWALCPPNYIPDWHVAVRRK<br>ADKKLLAFIAGVPVTLRMGTPK<br>YMKVKAQEKGEGEEAAKYDEP<br>RHICEINFLCVHKQLREKRLAPI<br>LIKEATRRVNRTNVWQAVYTA<br>GVLLPTPYASGQYFHRSLNPEK<br>LVEIRFSGIPAQYQKFQNPMAM<br>LKRNYQLPSAPKNSGLREMKPS<br>DVPQVRRILMNYLDSFDVGPVF<br>SDAEISHYLLPRDGVVFTYVVE<br>NDKKVTDFFSFYRIPSTVIGNSN<br>YNLLNAAYVHYYAATSIPLHQ<br>LILDLLIVAHSRGFDVCNMVEIL<br>DNRSFVEQLKFGAGDGHLRYY<br>FYNWAYPKIKPSQVALVML	411	Protein N-myristoylation, membrane association, and parasite survival [3]

#	Target protein	Structure source (PDB ID or homology model)	Organism	Amino-acid sequence used for docking (one-letter code)	Structure/ model length (aa)	Biological function
**4.**	Farnesyl diphosphate synthase (FPPS)	4JZX	*L. major*	MAHMERFQKVYEEVQEFLLGD AEKRFEMDVHRKGYLKSMMD TTCLGGKYNRGLCVVDVAEA MAKDTQMDAAAMERVLHDA CVCGWMIEMLQAHFLVEDDI MDHSKTRRGKPCWYLHPGVT AQVAINDGLILLAWATQMAL HYFADRPFLAEVLRVFHDVDLT TTIGQLYDVTSMVDSAKLDAK VAHANTTDYVEYTPFNHRRIV VYKTAYYTYWLPLVMGLLVSG TLEKVDKKATHKVAMVMGEY FQVQDDVMDCFTPPEKLGKIG TDIEDAKCSWLAVTFLTTAPAE KVAEFKANYGSTDPAAVAVIK QLYTEQNLLARFEEYEKAVVAE VEQLIAALEAQNAAFAASVKV LWSKTYKRQK	362	Isoprenoid biosynthesis and farnesyl diphosphate production for sterol biosynthesis and protein prenylation [4]
**5.**	Tryparedoxin peroxidase	4K1F	*L. major*	MSCGNAKINSPAPSFEEVALMP NGSFKKISLSSYKGKWVVLFFYP LDFTFVCPTEVIAFSDSVSRFNE LNCEVLACSIDSEYAHLQWTLQ DRKKGGLGTMAIPILADKTKNI ARSYGVLEESQGVAYRGLFIIDP HGMLRQITVNDMPVGRSVEEV LRLLEAFQFVEKHGEVCPANW KKGDPGMKPEPNASVEGYFSK Q	199	Peroxide detoxification and oxidative-stress defense through the trypanothione–tryparedoxin redox system [5]
**6.**	Arginase	5HJ9	*L. mexicana*	MRGSHHHHHHGMAKKMSIVL APFSGGQPHSGVELGPDYLLKQ GLQQDMEKLGWDTRLERVFD GKVVEARKASDNGDRIGRVKR PRLTAECTEKIYKCVRRVAEQG RFPLTIGGDHSIALGTVAGVLSV HPDAGVIWVDAHADINTMSG TVSGNLHGCPLSILLGLDRENIP ECFSWVPQVLKPNKIAYIGLRA VDDEEKKILHDLNIAAFSMHH VDRYGIDKVVSMAIEAVSPKGT EPVMVSYDVDTIDPLYVPATGT PVRGGLSFREALFLCERIAECGR LVALDVVECNPLLAATESHVN DTISVGCAIARCMMGETLLYTP HTSSKL	330	Arginine hydrolysis and *de novo* polyamine biosynthesis (L-arginine to L-ornithine and urea) [6]

#	Target protein	Structure source (PDB ID or homology model)	Organism	Amino-acid sequence used for docking (one-letter code)	Structure/ model length (aa)	Biological function
**7.**	Pteridine reductase 1 (PTR1)	6RXC	*L. major*	GSHMTAPTVPVALVTGAAKRL<br>GRSIAEGLHAEGYAVCLHYHR<br>SAAEANALSATLNARRPNSAIT<br>VQADLSNVATAPVSGADGSAP<br>VTLFTRCAELVAACYTHWGRC<br>DVLVNNASSFYPTPLLRNDEDG<br>HEPCVGDREAMETATADLFGS<br>NAIAPYFLIKAFAHRVAGTPAK<br>HRGTNYSIINMVDAMTNQPLL<br>GYTIYTMAKGALEGLTRSAALE<br>LAPLQIRVNGVGPGLSVLVDD<br>MPPAVWEGHRSKVPLYQRDSS<br>AAEVSDVVIFLCSSKAKYITGTC<br>VKVDGGYSLTRA	291	Pterin and folate salvage, with bypass-mediated antifolate resistance [7]
**8.**	Trypanothione disulfide reductase (TryR)	6T95	*L. infantum*	PMSRAYDLVVLGAGSGGLEAG<br>WNAAVTHKKKVAVVDVQAT<br>HGPPLFAALGGTCVNVGCVPK<br>KLMVTGAQYMDLIRESGGFGW<br>EMDRESLCPNWKTLIAAKNKV<br>VNSINESYKSMFADTEGLSFHM<br>GFGALQDAHTVVVRKSEDPHS<br>DVLETLDTEYILIATGSWPTRLG<br>VPGDEFCITSNEAFYLEDAPKR<br>MLCVGGGYIAVEFAGIFNGYKP<br>CGGYVDLCYRGDLILRGFDTEV<br>RKSLTKQLGANGIRVRTNLNPT<br>KITKNEDGSNHVHFNDGTEED<br>YDQVMLAIGRVPRSQALQLDK<br>AGVRTGKNGAVQVDAYSKTSV<br>DNIYAIGDVTNRVMLTPVAINE<br>GAAFVETVFGGKPRATDHTKV<br>ACAVFSIPPIGTCGMTEEEAAK<br>NYETVAVYASSFTPLMHNISGS<br>KHKEFMIRIITNESNGEVLGVH<br>MLGDSAPEIIQSVGICMKMGAK<br>ISDFHSTIGVHPTSAEELCSMRT<br>PAYFYESGKRVEKLS	489	NADPH-dependent trypanothione reduction, thiol-redox homeostasis, and oxidative-stress defense [8]
**9.**	Adenine phosphoribosyl-transferase (APRT)	SWISS-MODEL	*L. major* strain Friedlin	FKEISPNSFLLEDSHPLSQLLKK<br>NYCWYSPVFSPRNVPRFADVSS<br>ITESPETLKAIRDFLVQRYRTMS<br>PAPTHILGFDARGFLFGPMIAV<br>ELGIPFVLMRKADKNAGLLIRS<br>EPYEKEYKEAAPEVMTIRYGSIS<br>KGSRVVLIDDVLATGGTALSGL<br>QLVEASDAMVVEMVSILCIPFL<br>KAAEKIHSTGHSRYKDIKFISLL<br>SEEALTEDNCGDSKNYTGPRV<br>VSCGDVLSKH	233	Purine salvage through conversion of adenine and PRPP to AMP and pyrophosphate [9,10]

#	Target protein	Structure source (PDB ID or homology model)	Organism	Amino-acid sequence used for docking (one-letter code)	Structure/ model length (aa)	Biological function
**10.**	Deoxyhypusine hydroxylase (DOHH)	SWISS-MODEL	*L. major* strain Friedlin	MSASNSCTVEEVRKEYAKLLDP<br>QEPLDSRMRELYRLKEDCLKTV<br>AGVTVILEAIDTTDSVLLQHEL<br>AYNAGQSGREEAVPELERILRT<br>TSYDVVTRHEAAEALGAIGSPL<br>ALQVLETHSAPTTEPEASIRETC<br>ELALARIAMKETKGDAAVAPP<br>SGCEFVSVDPSPAFSALYSSTDE<br>PVPLTVEELEAVLLDTSGRTRLF<br>RRYMAMFTLRNLATEAAVAAL<br>CRGLREDTISALFRHEVAFVLG<br>QLERPSSQPALIAALKDEEEAP<br>MVRHEAAEALGAIADPATLPV<br>LESYATHHEPIVRDSCVVALEM<br>HKYWAHFNSLAHQQQEA	325	eIF5A hypusination and maturation through deoxyhypusine hydroxylation [9,10]
**11.**	Fructose-bisphosphate aldolase	SWISS-MODEL	*L. major* strain Friedlin	MSRVTIFQSQLPACNRIKTPYES<br>ELIATVKKLTTPGKGLLAADESI<br>GSCTKRFEPIGLSNTEEHRRQYR<br>ALMLEAEGLEQYISGVILHDET<br>VSQKASNGKTFPEYLTARGVVP<br>GIKTDMGLCPLLEGAEGEQMT<br>EGLDGYVKRASVYYKKGCRFC<br>KWRNVYKIQNGTVSEPAVRFN<br>AETLARYAILSQMSGLVPIVEPE<br>VMIDGKHDIDTCQRVSEHVWR<br>EVVAALQRHGVIWEGCLLKPN<br>MVVPGAESGQTAAPEQVAHYT<br>VMTLARTMPAMLPGVMFLSG<br>GLSEVQASEYLNAINNSPLPRP<br>YFLSFSYARALQSSALKAWGGK<br>DSGVAAGRRAFLHRARMNSM<br>AQLGKYKRADDD	358	Glycolysis through cleavage of fructose-1,6-bisphosphate into glyceraldehyde-3-phosphate and dihydroxyacetone phosphate [9,10]
**12.**	Glyceraldehyde-3-phosphate dehydrogenase (GAPDH)	SWISS-MODEL	*L. major* strain Friedlin	APIKVGINGFGRIGRMVLQAIC<br>DQGLIGNEIDVVAVVDMSTNA<br>EYFAYQMKYDTVHGRPKYTVE<br>AVKSTPSVKTPDVLVVNGHRIK<br>CVKAQRNPADLPWGKLGVDY<br>VIESTGLFTDKLQAEGHIKGGA<br>KKVVISAPASGGAKTIVMGVN<br>QHEYSPTSHHVVSNASCTTNCL<br>APIVHVLTKENFGIETGLMTTIH<br>SYTATQKTVDGVSLKDWRGGR<br>AAAINIIPSTTGAAKAVGMVIPS<br>TKGKLTGMSFRVPTPDVSVVDL<br>TFRSTRETSIQEIDKAIKKAAQT<br>YMKDILGFTNDELVSSDFINDN<br>RSSVYDSKATLQNNLPGEKRFF<br>KIVSWYDNEWGYSHRVVDLVR<br>YMAATDAASA	358	Glycolysis through NAD^+-dependent oxidation and phosphorylation of glyceraldehyde-3-phosphate [9,10]

#	Target protein	Structure source (PDB ID or homology model)	Organism	Amino-acid sequence used for docking (one-letter code)	Structure/ model length (aa)	Biological function
**13.**	Putative GPI mannosyl-transferase 1 (GPI14)	SWISS-MODEL	*L. major* strain Friedlin	MRNKTWLDRQSIGTLLLYGGL<br>ARVVLLIYAAFHDYYFRVKYTD<br>IDYMIVVDGARELLHGGTPFDR<br>TTYRYTPLLAVLVIPAVLIANPL<br>GKVVFALSDLGAAYYCFHMLL<br>RFSTERSAKWMVVILILFNPVVL<br>NVSTRGNSDMLISCMSMGVLA<br>KFAEGRYFTAASILGFAVHFKIY<br>PIIYALPLVLGVWERAKQERFF<br>GRLAHTASVVVGCGLCFTVAF<br>AVPTYVCYLVYGQQYLDEGFIY<br>HIHREDHRHNFSPYWLLMYLN<br>MGRRDLGVGVDYSAGLFAFLP<br>QFAVLCYASWKLRKNIAHACC<br>VETILFVAFNKVCTVQYFVWFL<br>PFLAFVFCEPAQPKWLTGVTN<br>APKSERPSMLSAVAAILMWSL<br>MIPLWVWTAYGLEFEGRNHYG<br>RLWIVSCVFYLATVSLAAWLGR<br>LCYRSRITVSIKDHRPASKRA	432	Glycosyl-phosphatidylinositol (GPI)-anchor biosynthesis through transfer of the first mannose to the GPI precursor [9,10]
**14.**	2,3-Bisphospho-glycerate-independent phosphor-glycerate mutase (iPGAM)	SWISS-MODEL	*L. major* strain Friedlin	LLLRPHKDLPRRKLLIVVMDGL<br>GIGPEDEYDAVHMASTPFMDA<br>QRQNSRHFRSVRAHGTAVGLP<br>TDADMGNSEVGHNALGAGRV<br>ALQGASLVDDALKSGEIYTGEG<br>YRYLLGAFTKEGSTLHLIGLLSD<br>GGVHSRDNQIHSIIEHAAKNG<br>AKGIRVHVLYDGRDVPDGSSFR<br>FTEELEAVLARVRQDGCDAAIA<br>SGGGRMFVTMDRYDADWSIVE<br>RGWKAQVLGDARHFHSAKEAI<br>TTFREEDPKVTDQYYPPFVVVD<br>EQGKPLGTIEDGDAVLCVNFR<br>GDRVIEMTRAFEDEDFDKFDRV<br>RVPKVRYAGMMRYDGDLGIPN<br>NFLVPPPKLTRVSEEYLCGTGL<br>QIFACSETQKFGHVTYFWNGN<br>RSGKVDEEHETFKEVPSDRVHF<br>NEQPKMKSAEITEAAIEALKSG<br>MYDVVRINFPNGDMVGHTGD<br>LRATIAGVEAVDESLAKLKDAV<br>DSINGVFIVTADHGNSDDMAQ<br>RDKKGKPMKDEKGNVLPLTSH<br>TLSPVPVFIGGAGLDPRVAMRT<br>DLPTAGLANVTATFINLLGFEA<br>PEDYEPSLIFVE	549	Glycolysis through cofactor-independent interconversion of 3-phosphoglycerate and 2-phosphoglycerate [9,10]

#	Target protein	Structure source (PDB ID or homology model)	Organism	Amino-acid sequence used for docking (one-letter code)	Structure/ model length (aa)	Biological function
**15.**	Pyruvate kinase	SWISS-MODEL	*L. major* strain Friedlin	SQLAHNLTLSIFEPVANHRATR IVCTIGPSTQSVEALKGLIQSGM SVARMNFSHGSHEYHQTTINN VRQAAAELGVNIAIALDTKGPE IRTGQFVGGEAVMERGATCYV TTDPAFADKGTKDKFYIDYQN LSKVVRPGSYIYIDDGILILHVQS HEDEQTLKCTVTNAHTISDRRG VNLPGCDVDLPAVSAKDCADL QFGVEQGVDMIFASFIRSAEQV GDVRKALGAKGHDIMIICKIEN HQGVQNIDSIIEESDGIMVARG DLGVEIPAEKVVVAQKILISKC NVAGKPVICATQMLESMTYNP RPTRAEVSDVANAVFNGADCV MLSGETAKGKYPNEVVQYMA RICLEAQSAVNEYVFFNSIKKLQ PIPMSAEEAVCSSAVNSVYETK AKVMVVLSNTGRSARLVAKYR PNCPIVCVTTRLQTCRQLNITQ GVESVFFDAEKLGHDEGKEQR VAMGVGFAKSKGYVQTGDYSV VIHADHKVKGYANQTRILLVE	498	Glycolysis and ATP production through conversion of phosphoenol-pyruvate to pyruvate [9,10]
**16.**	Squalene monooxygenase (squalene epoxidase)	SWISS-MODEL (UniProt: Q4QFZ2; NCBI RefSeq: XP 001681906.1	*L. major* strain Friedlin	MLYFFAAVLCAVSTLLLLNRTL SRLRLSPARTSYDYDVIIVGGSIA GPVLAKALSDQGRKVLMVERT LFTKPDRIVGELLQPGGLNALK EVGMKECAETIGMPCHGYVVV DHKGKQVDLPYRKGASGVSFH FGDFVQSLRSHVFHNCKANVT MVEGTVNNILTEGLSFSERAYG VEYTIAEKYEVPTKPFREDPPKT NPVAATVRKVATAPLVVMCD GGMSKWKSRYQHYTPAYEYHS HFIGLVLKTVRLPKEQRGTVFF GKTGPILSYRLDDNELRLLVDY NKPTLPSLEKQSEWLIQDVAPC LPENMREQFVRVSKDTKSLRSM PMARYPPAFPSIKGYVGIGDHA NQRHPLTGGGMTCCFRDAIRL ANSLNGIQSLRSVNQEEMAAIE DKIQAAILNYARYRYTHSCCIN LLSWALYSVFSSPALRDACFDY FLCGGNCVTGPMDLLAGLDPN VGSLFFHYCCVMLHGVANVM MRTGAYSESGKQLSNLEKLTN VASFFVDWERMKHAAYLLGKS TQIALPLAKSEFYSMWRFVDPT SPLANISKRIKTMVYTKQFNGK QRKPVGL	569	Sterol biosynthesis through epoxidation of squalene to 2,3-oxidosqualene [9,10]

#	Target protein	Structure source (PDB ID or homology model)	Organism	Amino-acid sequence used for docking (one-letter code)	Structure/ model length (aa)	Biological function
**17.**	Squalene synthase	SWISS-MODEL	*L. major* strain Friedlin	ETDLRFCYDIMNDVSRSFAVVVAQLADQQLRDAICIFYLVLRALDTLEDDMSVPVDVKLKELPKFHTHTSDMSWCMSGVGEGRERELLAKYPCVSREFKKLKKEYQDVIANICERMANGMCEFLKRPVVTKDDYNQYCHYVAGLVGHGLTQLFARCGFEDPSLDDDLTSSNHMGLFLQKTNIIRDYYEDIREEPPRMFWPKEIWGTYVTELKELKSESNNAAAVQCLNAMVADALVHVPYIVDYLSALRDPSVFRFCAIPQVMAIATLKEVYNNPDTFQVKVKVSRPESCRIMLKATTLYSSLSMFRDYCVELQEKLDMQDASSVSIANSLAAAIERID	339	Sterol biosynthesis through formation of squalene from two farnesyl diphosphate molecules [9,10]
**18.**	Triosephosphate isomerase	SWISS-MODEL	*L. major* strain Friedlin	AKPQPIAAANWKCNGTTASIEKLVQVLNEHNISHDVQCVVAPTFVHIPLVQAKLRNPKYVVSAENAIAKSGAFTGEVSMPILKDLGINWVILGHSERRTYYGETDETVAQKVADACKQGFMVIACIGETLQQREANQTAKVVLSQTSAIATKLPKEAWDQIVLAYEPVWAIGTGKVATPEQAQEVHALLRKWVSEKIGTDVAAKLRILYGGSVSAGNAKTLYMKPDINGFLVGGASLKPEFRDIIDATR	249	Glycolysis through reversible interconversion of dihydroxyacetone phosphate and glyceraldehyde-3-phosphate [9,10]
**19.**	Xanthine phosphoribosyl-transferase (XPRT)	SWISS-MODEL	*L. major* strain Friedlin	DAQGRVFVDGREYPMASGIVATEDVIQANIKAMANTIAKDYKSLSHRDVRLSPGTAATAEAAEAPISYDNPLIIISVLKGSYIFTSDFIRYLGDCGLPHVVDFVRLASYNSDTTSTGQISMLAGLRFENLRGKHVLIVEDVCDSGRTLRFLRDYIMEKFQPKSVKTLVMVNKEQAARKLDFDPEYFCLAGPNKYIVGYGFEVNDRYRDLRHIFILRDGEATRYPAKL	227	Purine salvage through conversion of xanthine and PRPP to XMP and pyrophosphate [9,10]

Note: Rows 9–19 are homology models generated using SWISS-MODEL [11]. The displayed amino-acid sequence and length correspond to the structure used for docking and may differ from the complete database protein.

References (Table S2)

1. Fyfe, P.K.; Oza, S.L.; Fairlamb, A.H.; Hunter, W.N. *Leishmania* trypanothione synthetase-amidase structure reveals a basis for regulation of conflicting synthetic and hydrolytic activities. *J. Biol. Chem.* **2008**, *283*, 17672–17680. https://doi.org/10.1074/jbc.M801850200.
2. Hargrove, T.Y.; Wawrzak, Z.; Liu, J.; Nes, W.D.; Waterman, M.R.; Lepesheva, G.I. Substrate preferences and catalytic parameters determined by structural characteristics of sterol 14α-demethylase (CYP51) from *Leishmania infantum*. *J. Biol. Chem.* **2011**, *286*, 26838–26848. https://doi.org/10.1074/jbc.M111.237099.

3. Brannigan, J.A.; Roberts, S.M.; Bell, A.S.; Hutton, J.A.; Hodgkinson, M.R.; Tate, E.W.; Leatherbarrow, R.J.; Smith, D.F.; Wilkinson, A.J. Diverse modes of binding in structures of *Leishmania major* N-myristoyltransferase with selective inhibitors. *IUCrJ* **2014**, *1*, 250–260. https://doi.org/10.1107/S2052252514013001.
4. Aripirala, S.; Gonzalez-Pacanowska, D.; Oldfield, E.; Kaiser, M.; Amzel, L.M.; Gabelli, S.B. Structural and thermodynamic basis of the inhibition of *Leishmania major* farnesyl diphosphate synthase by nitrogen-containing bisphosphonates. *Acta Crystallogr. D Biol. Crystallogr.* **2014**, *70*, 802–810. https://doi.org/10.1107/S1399004713033221.
5. Brindisi, M.; Brogi, S.; Relitti, N.; Vallone, A.; Butini, S.; Gemma, S.; Novellino, E.; Colotti, G.; Angiulli, G.; Di Chiaro, F.; et al. Structure-based discovery of the first non-covalent inhibitors of *Leishmania major* tryparedoxin peroxidase by high throughput docking. *Sci. Rep.* **2015**, *5*, 9705. https://doi.org/10.1038/srep09705.
6. Hai, Y.; Christianson, D.W. Crystal structures of *Leishmania mexicana* arginase complexed with α,α-disubstituted boronic amino-acid inhibitors. *Acta Crystallogr. F Struct. Biol. Commun.* **2016**, *72*, 300–306. https://doi.org/10.1107/S2053230X16003630.
7. Pöhner, I.; Quotadamo, A.; Panecka-Hofman, J.; Luciani, R.; Santucci, M.; Linciano, P.; Landi, G.; Di Pisa, F.; Dello Iacono, L.; Pozzi, C.; et al. Multitarget, selective compound design yields potent inhibitors of a kinetoplastid pteridine reductase 1. *J. Med. Chem.* **2022**, *65*, 9011–9033. https://doi.org/10.1021/acs.jmedchem.2c00232.
8. de Lucio, H.; Revuelto, A.; Carriles, A.A.; de Castro, S.; García-González, S.; García-Soriano, J.C.; Alcón-Calderón, M.; Sánchez-Murcia, P.A.; Hermoso, J.A.; Gago, F.; et al. Identification of 1,2,3-triazolium salt-based inhibitors of *Leishmania infantum* trypanothione disulfide reductase with enhanced antileishmanial potency in cellulo and increased selectivity. *Eur. J. Med. Chem.* **2022**, *244*, 114878. https://doi.org/10.1016/j.ejmech.2022.114878.
9. UniProt Consortium. *Leishmania major* Reference Proteome, Proteome ID UP000000542. Available online: https://www.uniprot.org/proteomes/UP000000542 (accessed on 24 June 2026).
10. Ivens, A.C.; Peacock, C.S.; Worthey, E.A.; Murphy, L.; Aggarwal, G.; Berriman, M.; Sisk, E.; Rajandream, M.A.; Adlem, E.; Aert, R.; et al. The genome of the kinetoplastid parasite, *Leishmania major*. *Science* **2005**, *309*, 436–442. https://doi.org/10.1126/science.1112680.
11. Waterhouse, A.; Bertoni, M.; Bienert, S.; Studer, G.; Tauriello, G.; Gumienny, R.; Heer, F.T.; de Beer, T.A.P.; Rempfer, C.; Bordoli, L.; et al. SWISS-MODEL: Homology modelling of protein structures and complexes. *Nucleic Acids Res.* **2018**, 46, W296–W303. https://doi.org/10.1093/nar/gky427.

Table S3. Experimentally resolved HIV-1 reverse transcriptase-associated RNase H structure used for molecular docking and molecular dynamics with HPip-4NO, HPip-DC, and RDS1759. The table summarizes the protein identity, Protein Data Bank (PDB) accession, source organism, deposited PDB construct sequence, construct length, and principal biological function.

#	Target protein	PDB ID	Organism	PDB construct sequence (one-letter code)	Construct length (aa)	Biological function
**1.**	HIV-1 reverse transcriptase-associated RNase H domain	3K2P	Human immunodeficiency virus type 1, isolate BH10	ALYQLEKEPIVGAETFYVDGAANRETKLGKAGYVTNKGRQKVVPLTNTTNQKTELQAIYLALQDSGLEVNIVTDSQYALGIIQAQPDKSESELVNQIIEQLIKKEKVYLAWVPAHKGIGGNEQVDKLVSAGIRKIL	136	Reverse transcription and viral replication: cleavage of RNA in RNA–DNA hybrids during synthesis of proviral DNA [1]

Note: PDB 3K2P contains the isolated HIV-1 BH10 RNase H domain co-crystallized with β-thujaplicinol at the RNase H active site.

Reference (Table S3)

1. Himmel, D.M.; Maegley, K.A.; Pauly, T.A.; Bauman, J.D.; Das, K.; Dharia, C.; Clark, A.D., Jr.; Ryan, K.; Hickey, M.J.; Love, R.A.; et al. Structure of HIV-1 reverse transcriptase with the inhibitor β-thujaplicinol bound at the RNase H active site. *Structure* **2009**, *17*, 1625–1635. https://doi.org/10.1016/j.str.2009.09.016.

Table S4. Predicted protein–ligand interactions of the selected docked complexes. The table summarizes hydrogen bonds, hydrophobic interactions, other close contacts, π–cation interactions, and halogen bonds identified within the predicted binding sites. Interaction distances are given in ångströms (Å), and n indicates the number of contacts involving the corresponding residue. A dash (–) indicates that no interaction of the specified type was detected.

#	Complex	H-Bonds (Å)	True Hydrophobic (Å)	Other Close Contacts (Å)	π–Cation (Å)	Halogen (Å)
**1.**	Squalene monooxygenase (model)-HPip-DC	TYR269(A) (3.07 Å; n=1)	LEU279(A) (3.46 Å; n=1), LEU267(A) (3.50 Å; n=1), PHE240(A) (3.65 Å; n=1), LEU450(A) (3.75 Å; n=1), PRO353(A) (3.96 Å; n=1)	TYR107(A) (3.50 Å; n=3), TYR269(A) (3.74 Å; n=2)	–	MET447(A) (3.69 Å; n=1)
**2.**	Squalene monooxygenase (model)-amphotericin B	GLY106(A) (2.67 Å; n=1), LEU354(A) (2.82 Å; n=1), GLY345(A) (3.08 Å; n=1), ASP346(A) (3.11 Å; n=1), GLN350(A) (3.33 Å; n=1), GLY77(A) (3.40 Å; n=1), PRO327(A) (3.42 Å; n=1), GLN81(A) (3.43 Å; n=1), THR355(A) (3.44 Å; n=1)	LEU79(A) (3.28 Å; n=2), PHE240(A) (3.39 Å; n=1), PRO82(A) (3.63 Å; n=1), LEU279(A) (3.76 Å; n=1), LEU267(A) (3.87 Å; n=1)	TYR269(A) (3.35 Å; n=3), GLN81(A) (3.37 Å; n=1), TYR107(A) (3.45 Å; n=2), TYR121(A) (3.76 Å; n=1)	–	–
**3.**	Bovine mitochondrial cytochrome *bc* $_1$ (6XVF)-DiFiD	–	ALA127(A) (3.53 Å; n=1), ALA84(A) (3.73 Å; n=1), PRO134(A) (3.86 Å; n=1)	TYR131(A) (3.53 Å; n=2), GLN44(A) (3.74 Å; n=1)	–	ARG80(A) (3.83 Å; n=1)
**4.**	Bovine mitochondrial cytochrome *b* (6XVF)-atovaquone	ARG80(A) (3.17 Å; n=1), ASN255(A) (3.30 Å; n=1), GLY130(A) (3.50 Å; n=1)	ALA87(A) (3.31 Å; n=1), ALA84(A) (3.50 Å; n=1), ALA127(A) (3.61 Å; n=1)	TYR131(A) (3.84 Å; n=1), GLN44(A) (3.91 Å; n=1), THR126(A) (3.93 Å; n=1)	–	–
**5.**	HIV-1 RT-associated RNase H (3K2P)-HPip-DC	–	–	ASN474(A) (3.96 Å; n=1)	ARG557(A) (4.69 Å; n=1)	–
**6.**	HIV-1 RT-associated RNase H (3K2P)-HPip-4NO	–	LEU560(A) (3.22 Å; n=1), ALA538(A) (3.65 Å; n=1), ALA445(A) (3.89 Å; n=1)	–	–	–
**7.**	HIV-1 RT-associated RNase H (3K2P)-RDS1759	–	ALA538(A) (3.94 Å; n=1)	ASN474(A) (3.69 Å; n=1)	–	–

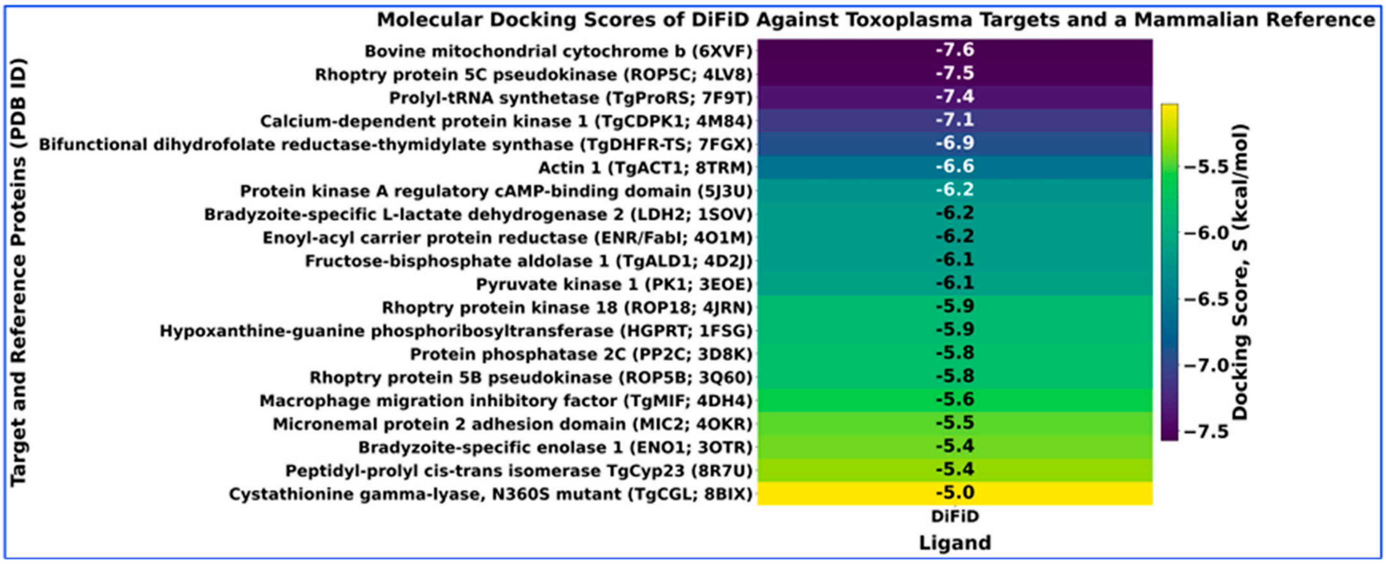

Figure S1. Molecular docking scores of DiFiD against selected *T. gondii*-associated proteins and the bovine mitochondrial cytochrome bc_1 complex (PDB ID: 6XVF), included as an experimentally resolved mammalian structural reference. The heat map presents predicted docking scores obtained using the applied docking protocol. More negative values indicate more favorable predicted docking scores and should not be interpreted as experimental binding affinities.

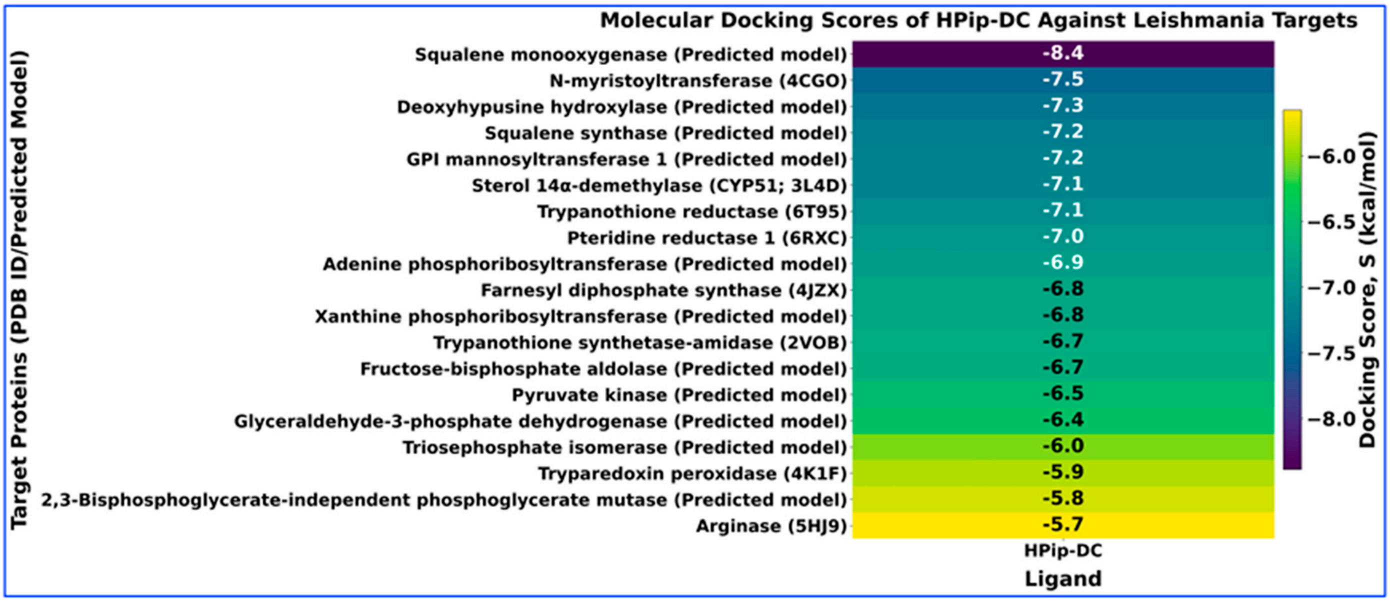

Figure S2. Molecular docking scores of HPip-DC against selected *Leishmania*-associated protein targets. The heat map includes experimentally resolved structures and computationally predicted protein models. More negative values indicate more favorable predicted docking scores within the applied docking protocol and should not be interpreted as experimental binding affinities.

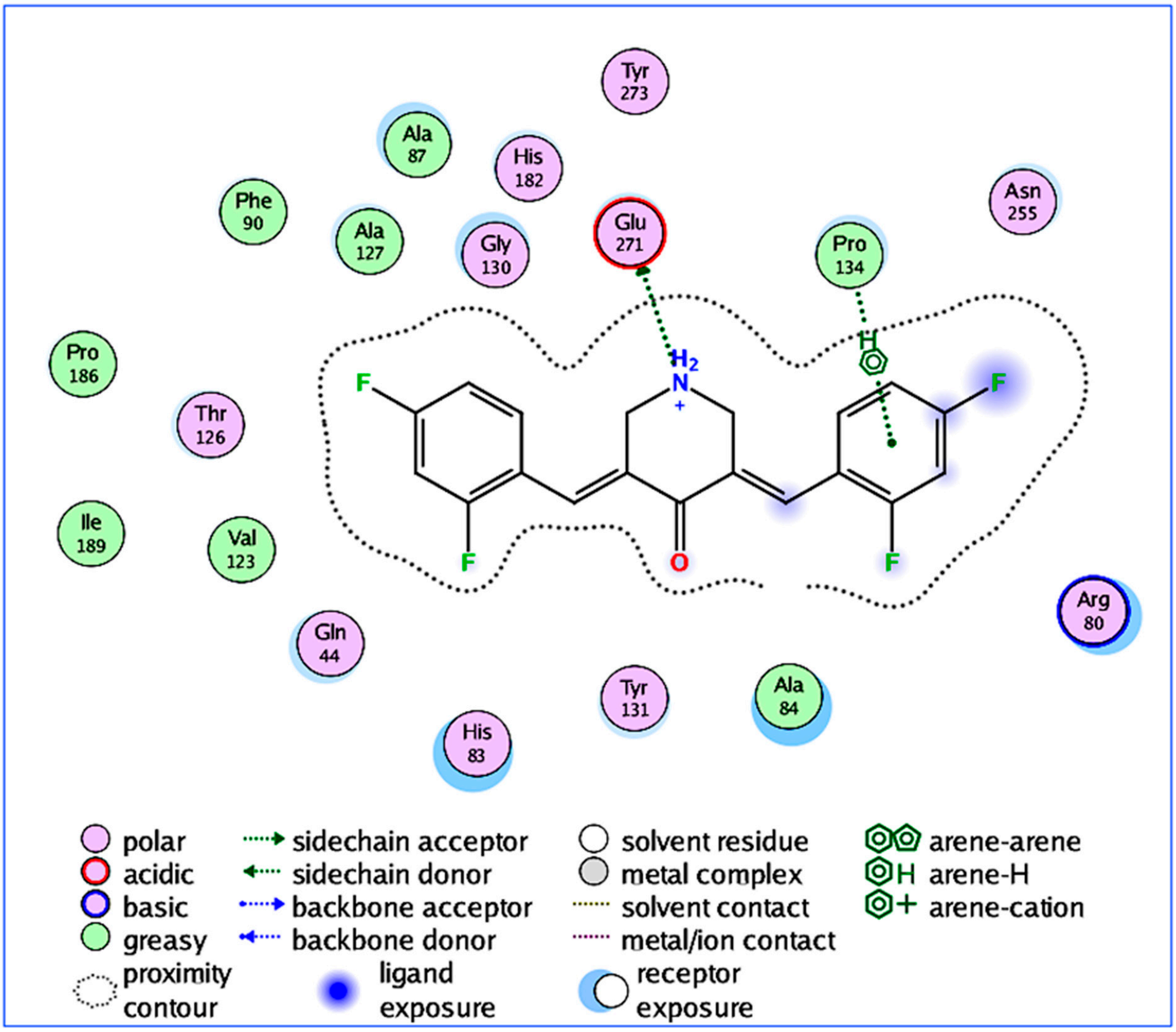

Figure S3. 2D interaction map of DiFiD bound to bovine mitochondrial cytochrome *bc₁* (PDB ID: 6XVF). The diagram illustrates the predicted binding orientation of DiFiD within the cytochrome *bc₁* binding pocket, together with the surrounding amino acid residues, pocket contour, ligand exposure, and predicted intermolecular contacts.

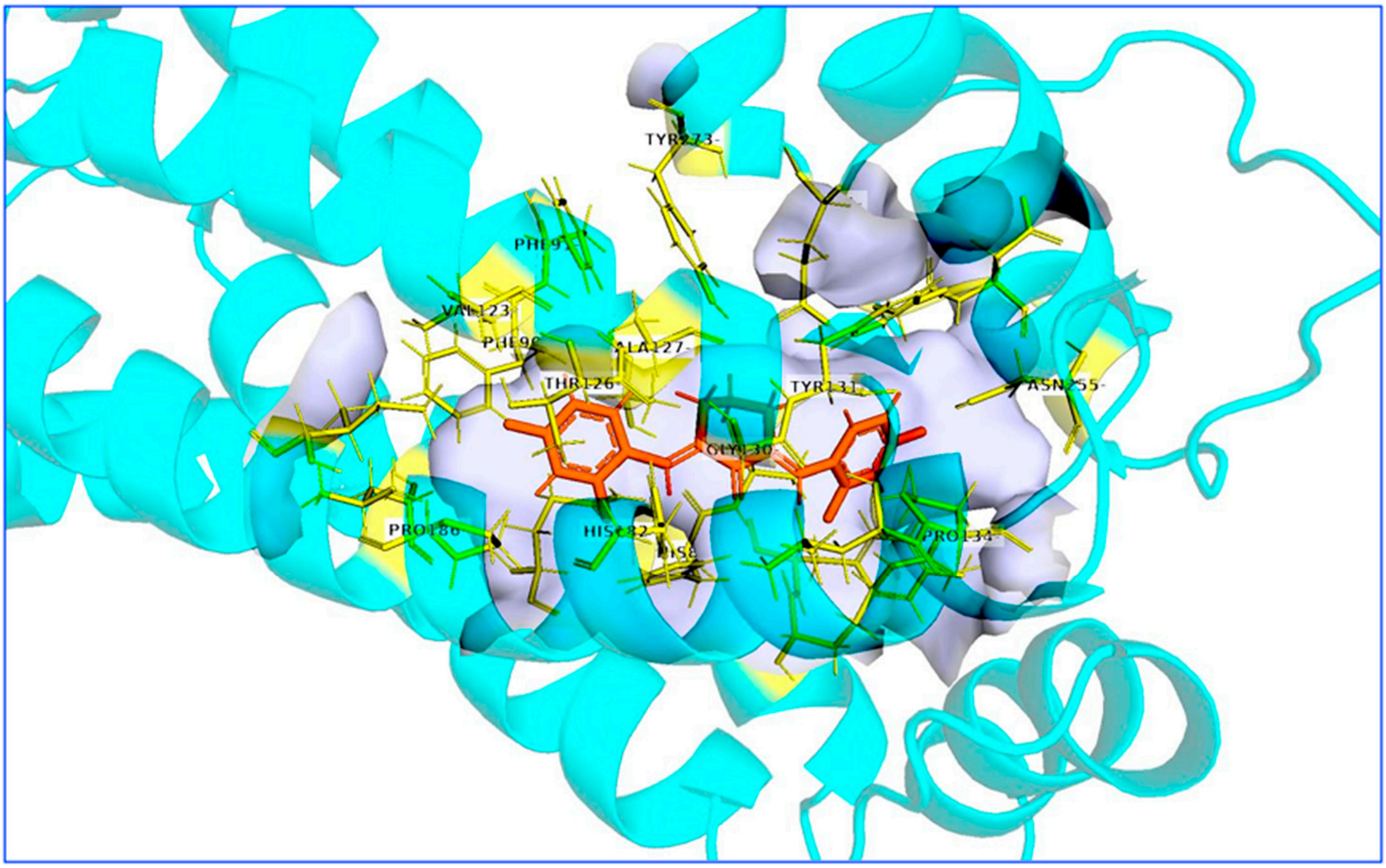

Figure S4. 3D binding pose of DiFiD within bovine mitochondrial cytochrome bc_1 (PDB ID: 6XVF). DiFiD is shown within the surface-rendered binding pocket together with the surrounding amino acid residues and protein secondary structure.

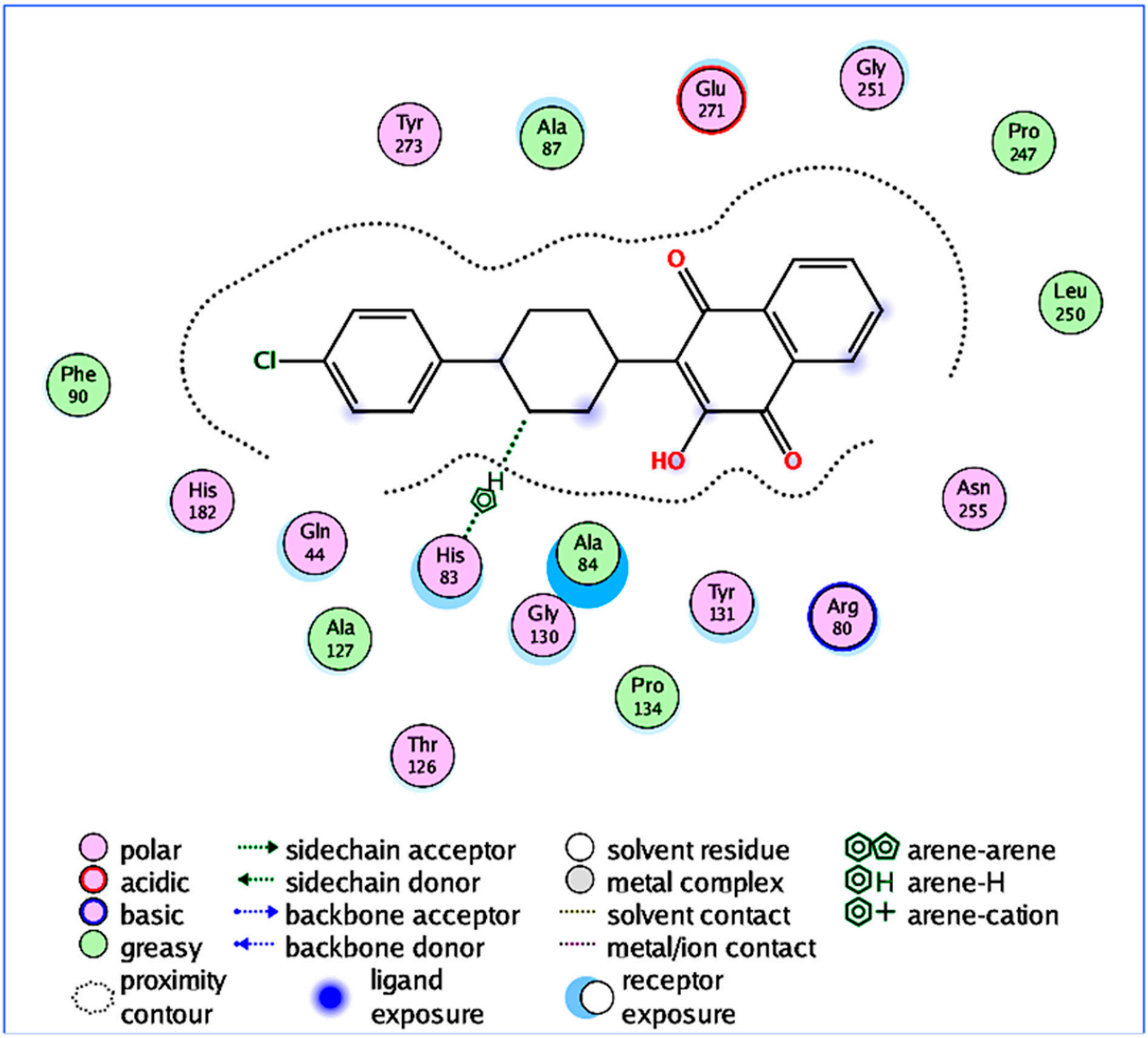

Figure S5. 2D interaction map of atovaquone bound to bovine mitochondrial cytochrome *bc*$_1$ (PDB ID: 6XVF). The diagram illustrates the predicted binding orientation of atovaquone within the cytochrome *bc*$_1$ binding pocket, together with the surrounding amino acid residues, pocket contour, ligand exposure, and predicted intermolecular contacts.

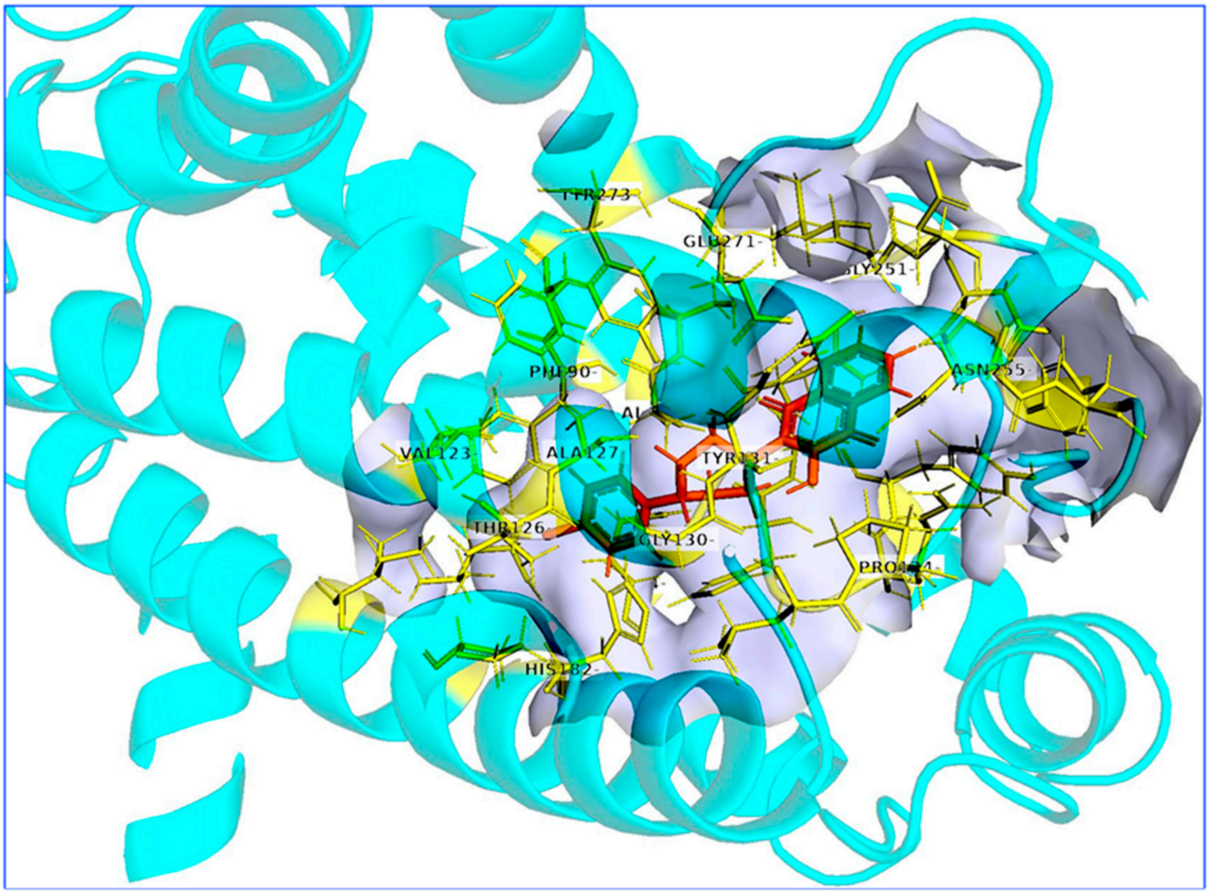

Figure S6. 3D binding pose of atovaquone within bovine mitochondrial cytochrome *bc₁* (PDB ID: 6XVF). Atovaquone is shown within the surface-rendered binding pocket together with the surrounding amino acid residues and protein secondary structure.

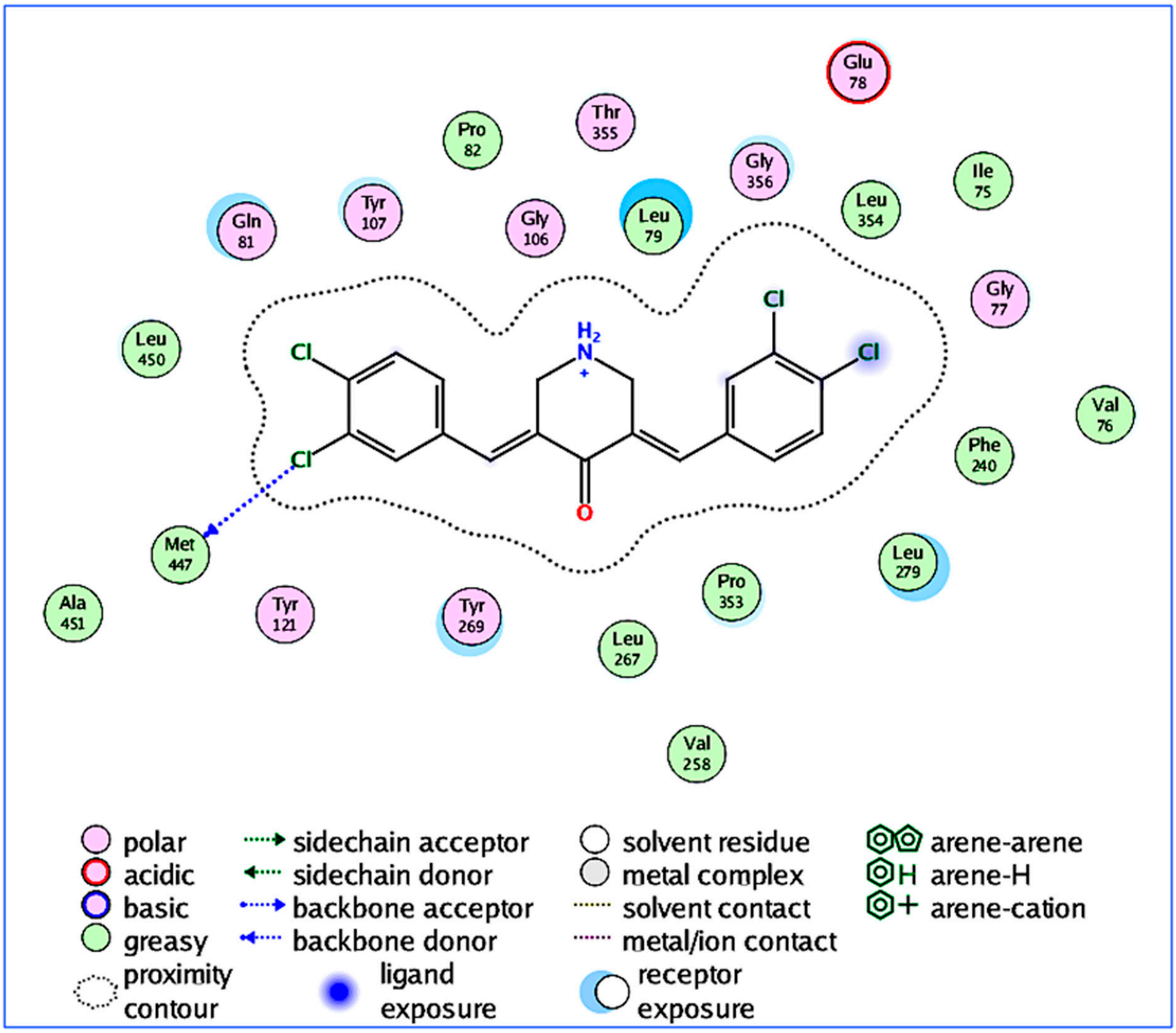

Figure S7. Two-dimensional (2D) interaction map of HPip-DC bound to the predicted *Leishmania major* squalene monooxygenase model. The diagram shows the predicted binding orientation of HPip-DC, the binding-pocket contour, ligand and receptor exposure, surrounding amino acid residues, and the principal predicted intermolecular contacts.

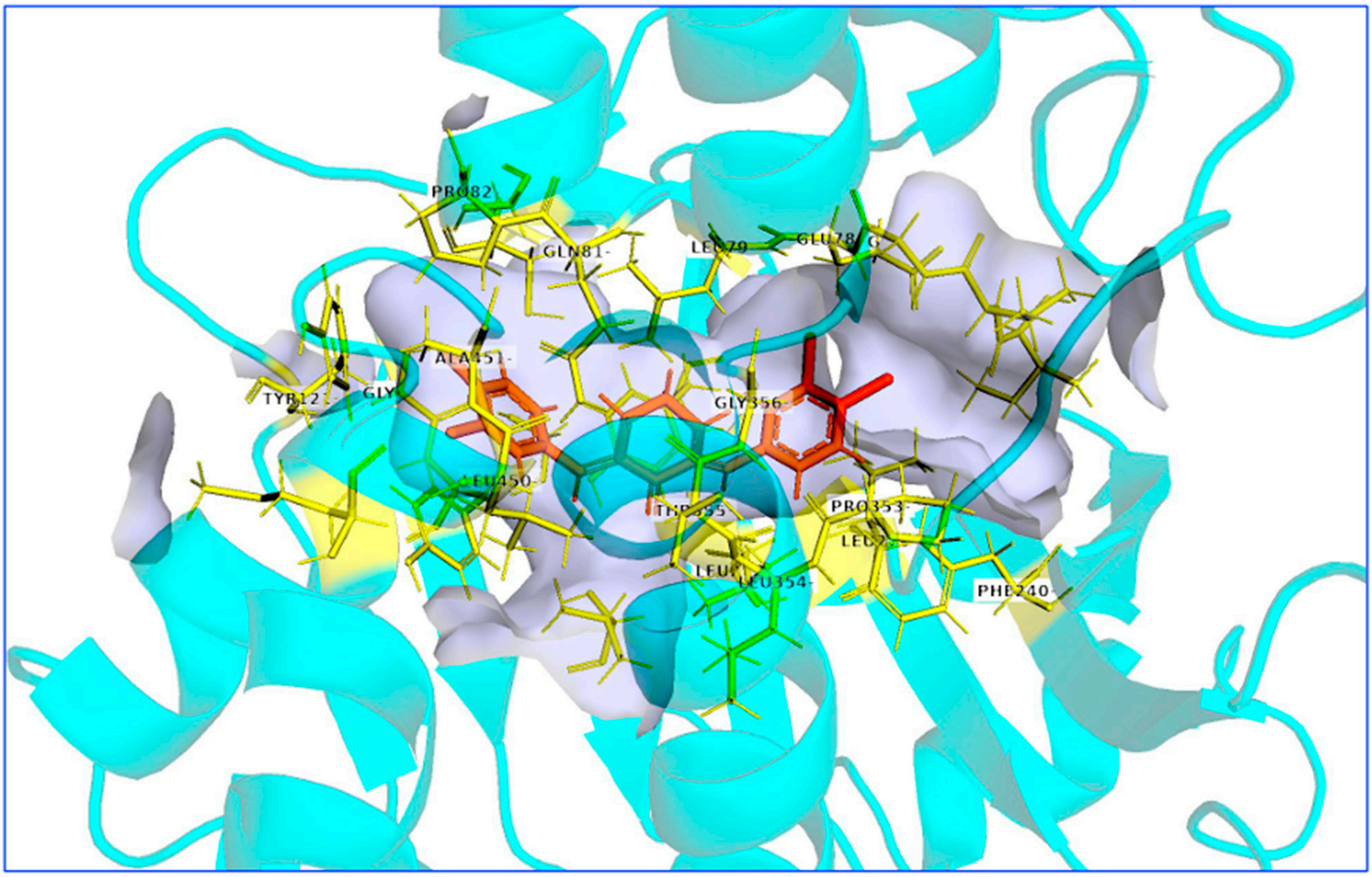

Figure S8. Three-dimensional (3D) binding pose of HPip-DC within the predicted *Leishmania major* squalene monooxygenase model. HPip-DC is shown within the surface-rendered binding pocket together with the surrounding amino acid residues and protein secondary structure.

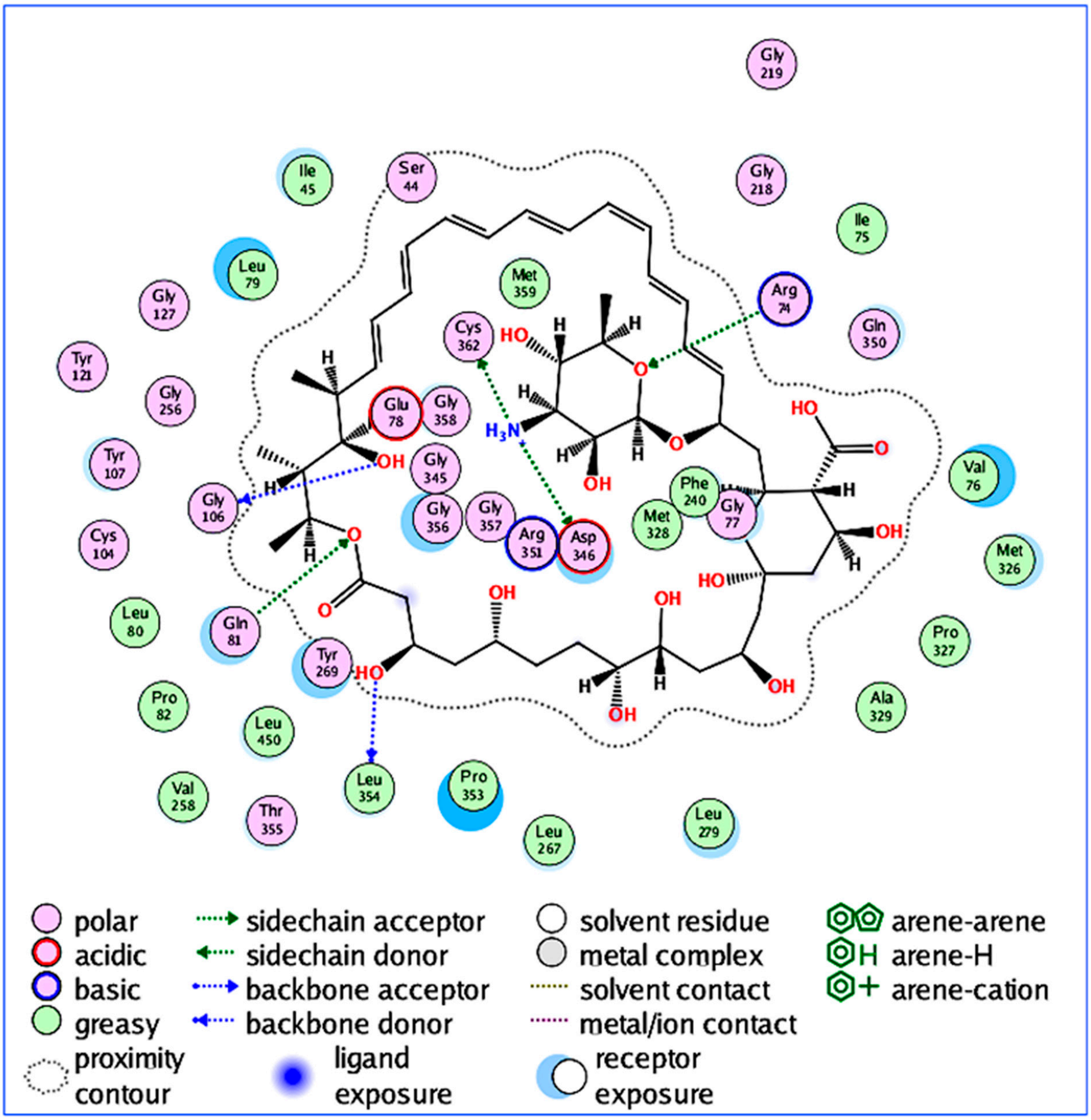

Figure S9. 2D interaction map of amphotericin B bound to the predicted *Leishmania major* squalene monooxygenase model. The diagram shows the predicted binding orientation of amphotericin B, the binding-pocket contour, ligand and receptor exposure, surrounding amino acid residues, and the principal predicted intermolecular contacts.

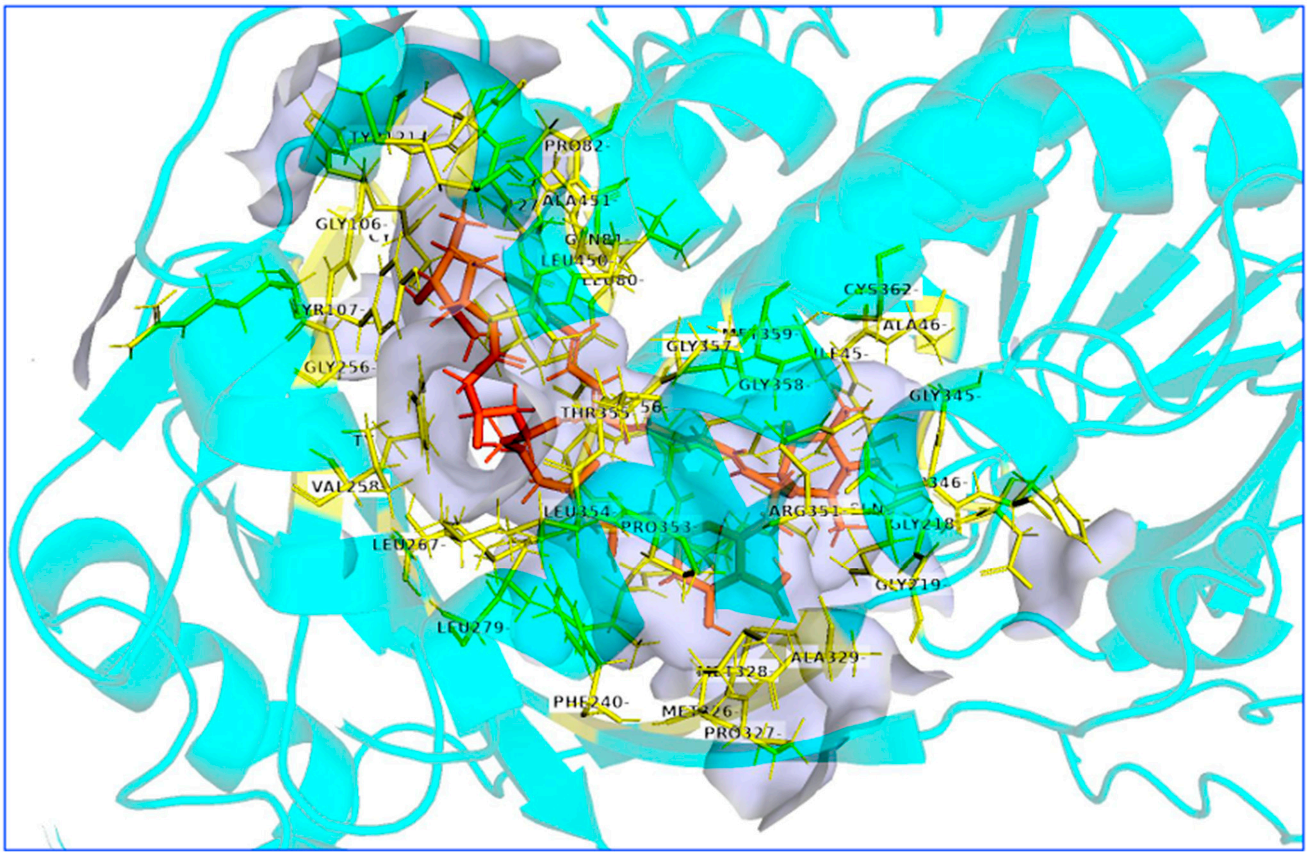

Figure S10. 3D binding pose of amphotericin B within the predicted *Leishmania major* squalene monooxygenase model. Amphotericin B is shown within the surface-rendered binding pocket together with the surrounding amino acid residues and protein secondary structure.

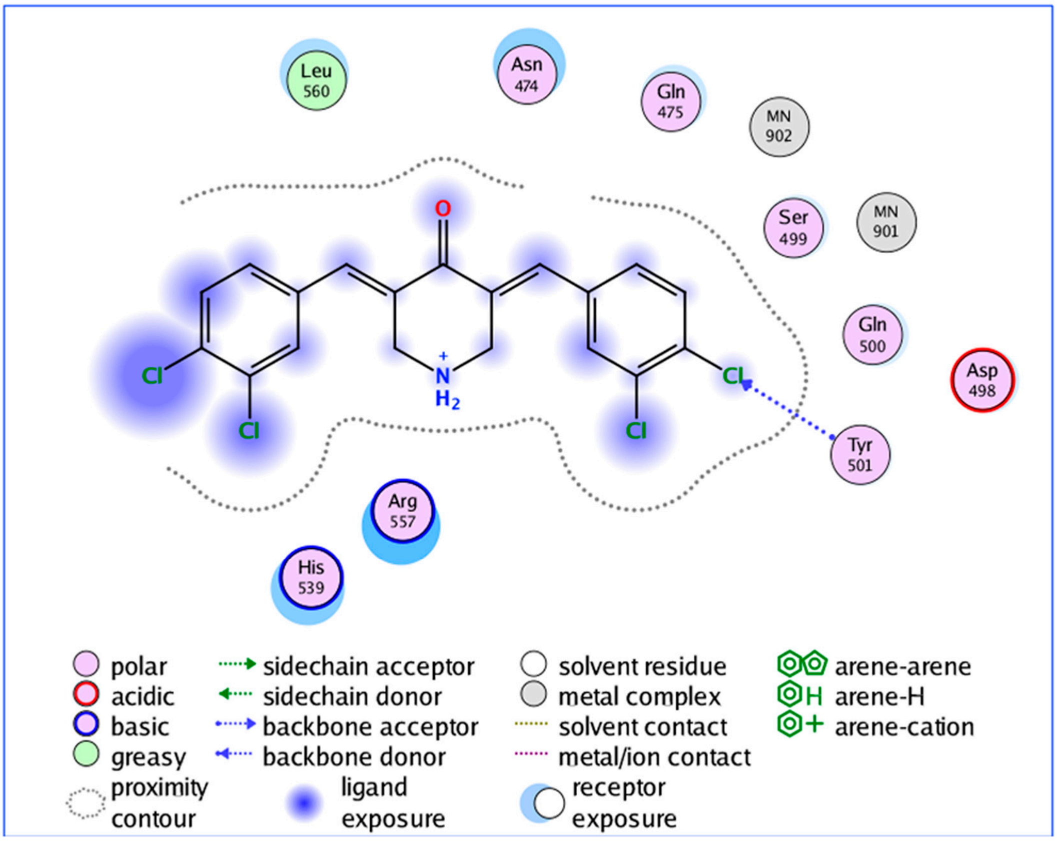

Figure S11. 2D interaction map of HPip-DC bound to experimentally resolved HIV-1 RT-associated RNase H structure (PDB ID: 3K2P). The diagram shows the predicted binding orientation of HPip-DC, the surrounding amino acid residues, binding-pocket contour, ligand and receptor exposure, and the catalytic metal ions present in the docking site.

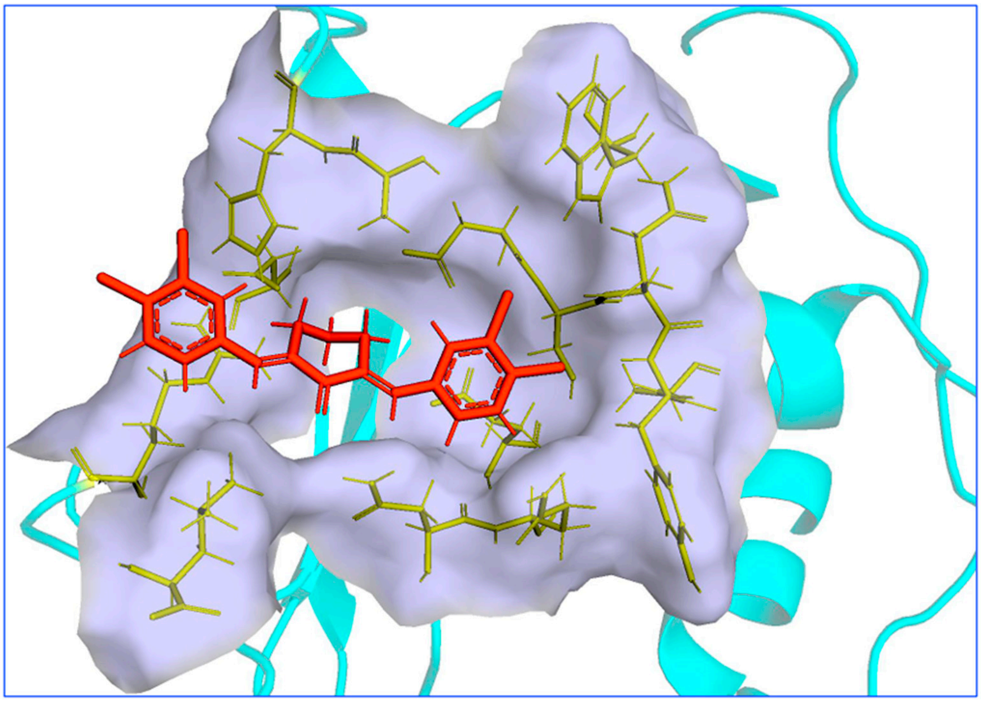

Figure S12. 3D binding pose of HPip-DC within experimentally resolved HIV-1 RT-associated RNase H structure (PDB ID: 3K2P). HPip-DC is shown within the surface-rendered RNase H binding pocket together with the surrounding amino acid residues and protein secondary structure.

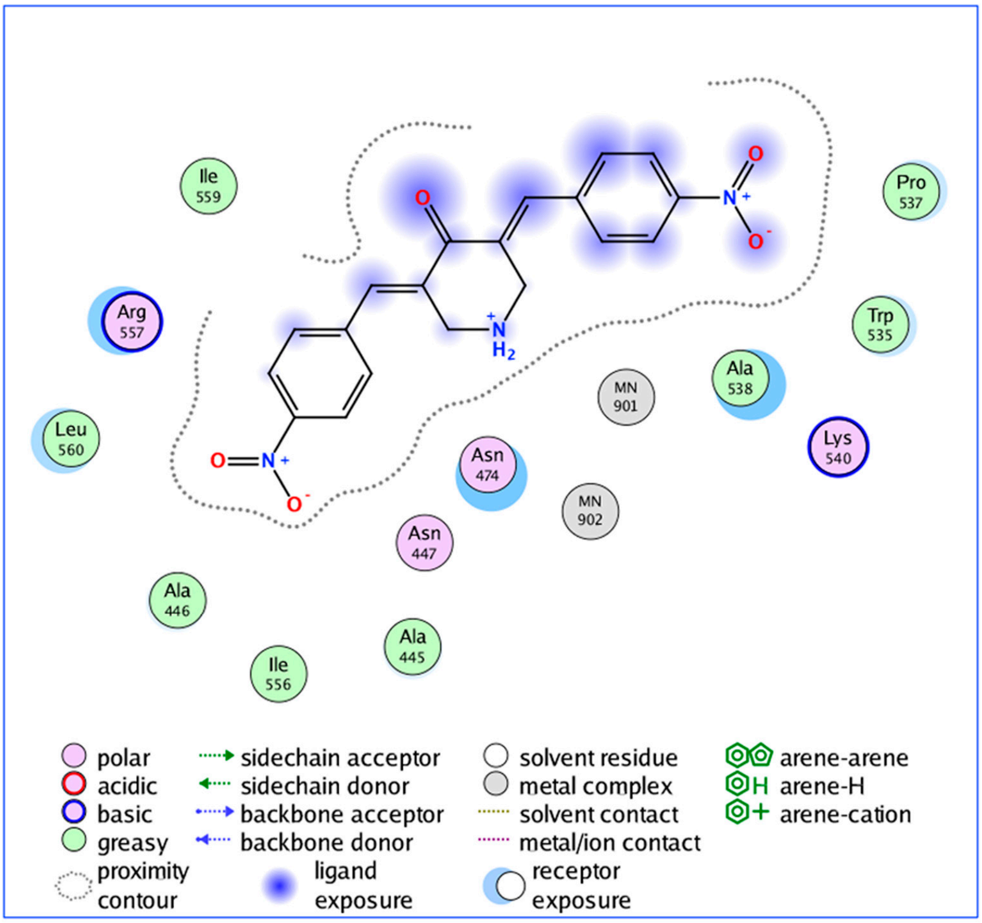

Figure S13. 2D interaction map of HPip-4NO bound to experimentally resolved HIV-1 RT-associated RNase H structure (PDB ID: 3K2P). The diagram shows the predicted binding orientation of HPip-4NO, the surrounding amino acid residues, binding-pocket contour, ligand and receptor exposure, and the catalytic metal ions present in the docking site.

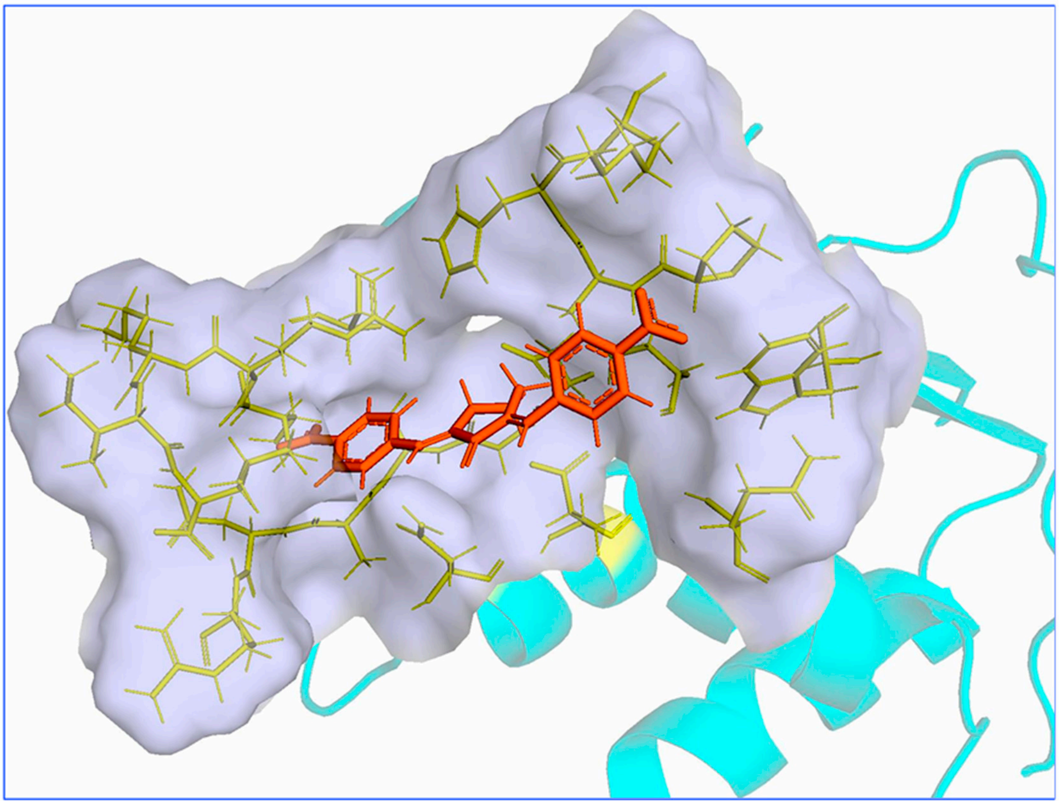

Figure S14. 3D binding pose of HPip-4NO within experimentally resolved HIV-1 RT-associated RNase H structure (PDB ID: 3K2P). HPip-4NO is shown within the surface-rendered RNase H binding pocket together with the surrounding amino acid residues and protein secondary structure.

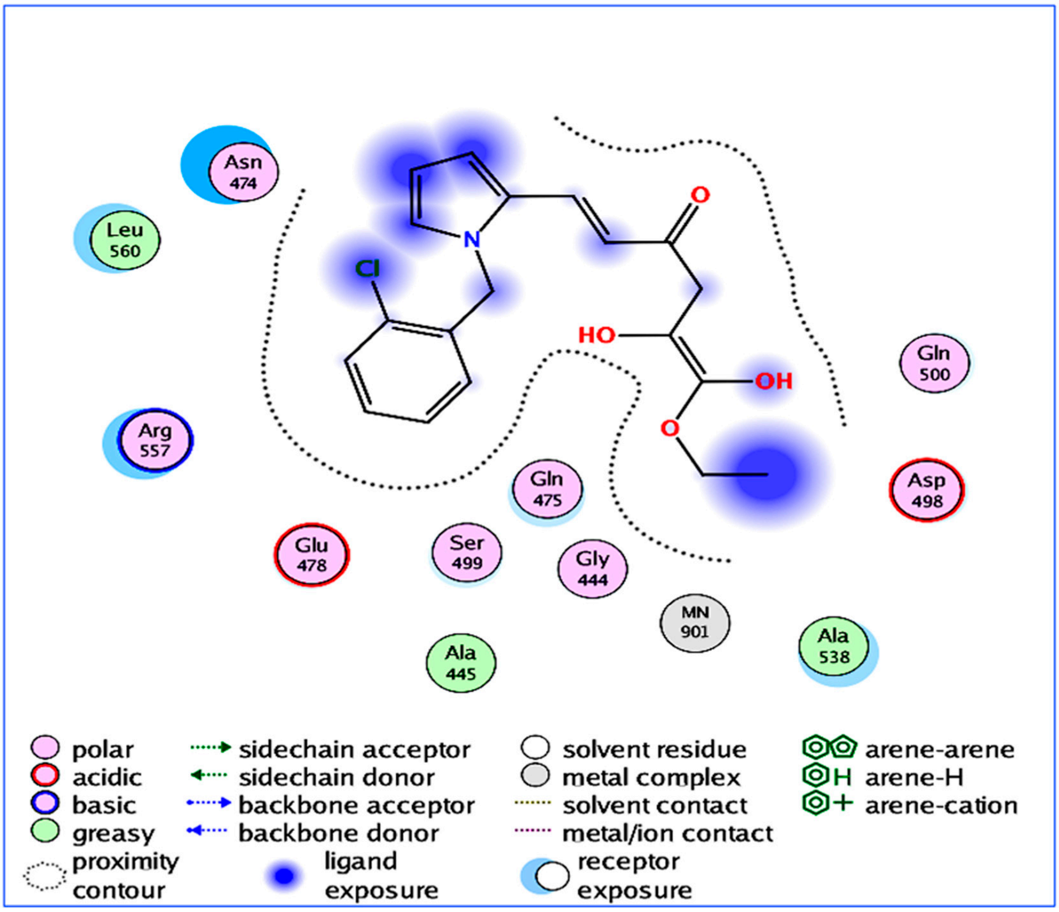

Figure S15. 2D interaction map of RDS1759 bound to experimentally resolved HIV-1 RT-associated RNase H structure (PDB ID: 3K2P). The diagram shows the predicted binding orientation of RDS1759, the surrounding amino acid residues, binding-pocket contour, ligand and receptor exposure, and the catalytic metal ions present in the docking site.

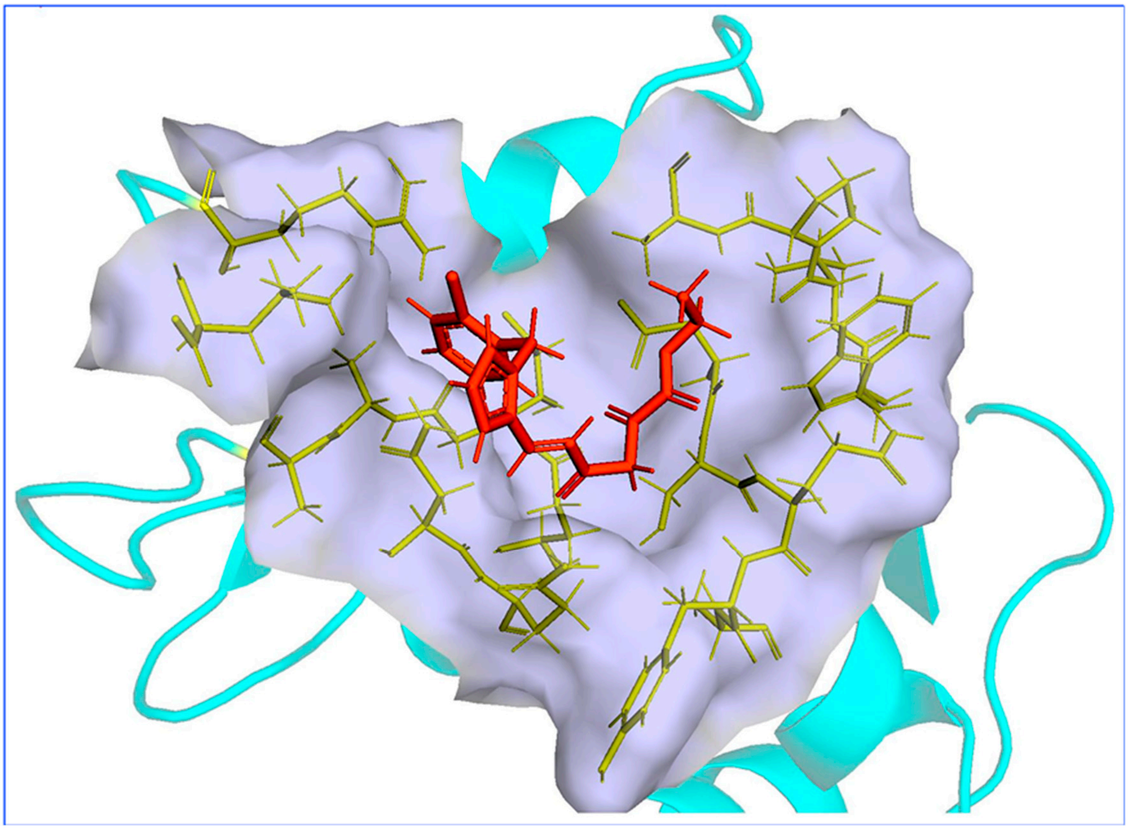

Figure S16. 3D binding pose of RDS1759 within experimentally resolved HIV-1 RT-associated RNase H structure (PDB ID: 3K2P). RDS1759 is shown within the surface-rendered RNase H binding pocket together with the surrounding amino acid residues and protein secondary structure.

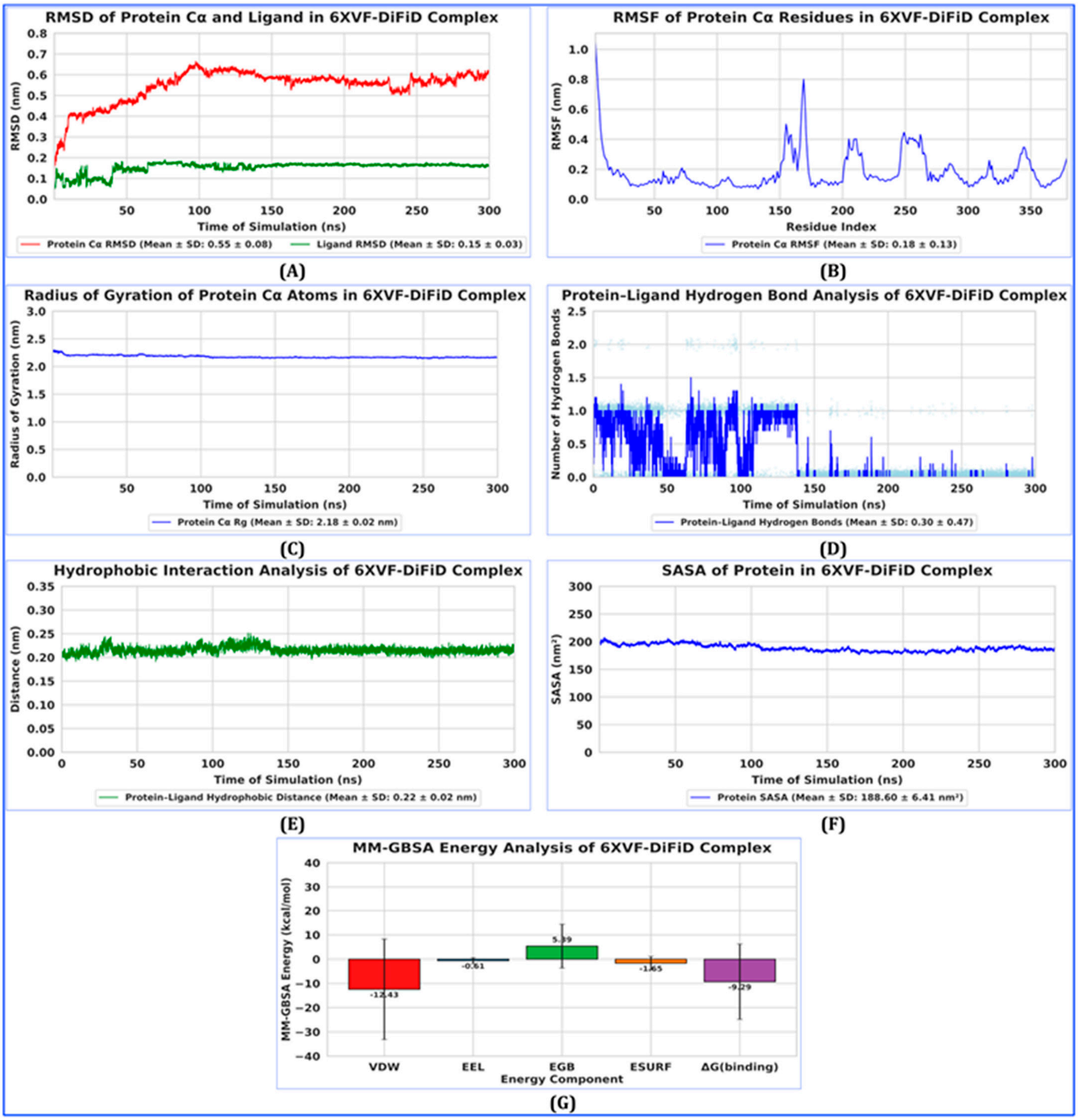

Figure S17. Molecular dynamics and MM-GBSA analyses of DiFiD bound to the experimentally resolved bovine mitochondrial cytochrome bc_1 complex (PDB ID: 6XVF) used as a mammalian structural reference. The panels show (A) protein Cα and ligand RMSD, (B) protein Cα RMSF, (C) radius of gyration of the protein Cα atoms, (D) protein–ligand hydrogen-bond analysis, (E) the representative protein–ligand hydrophobic-contact distance, (F) protein SASA, and (G) the MM-GBSA energy components and total binding-energy estimate. The structural descriptors in panels A–F were evaluated over the 300 ns simulation, whereas the MM-GBSA analysis was performed using 200 evenly distributed frames extracted from the 200–300-ns trajectory segment. The entropy-uncorrected MM-GBSA binding-energy estimate was −9.29 kcal/mol.

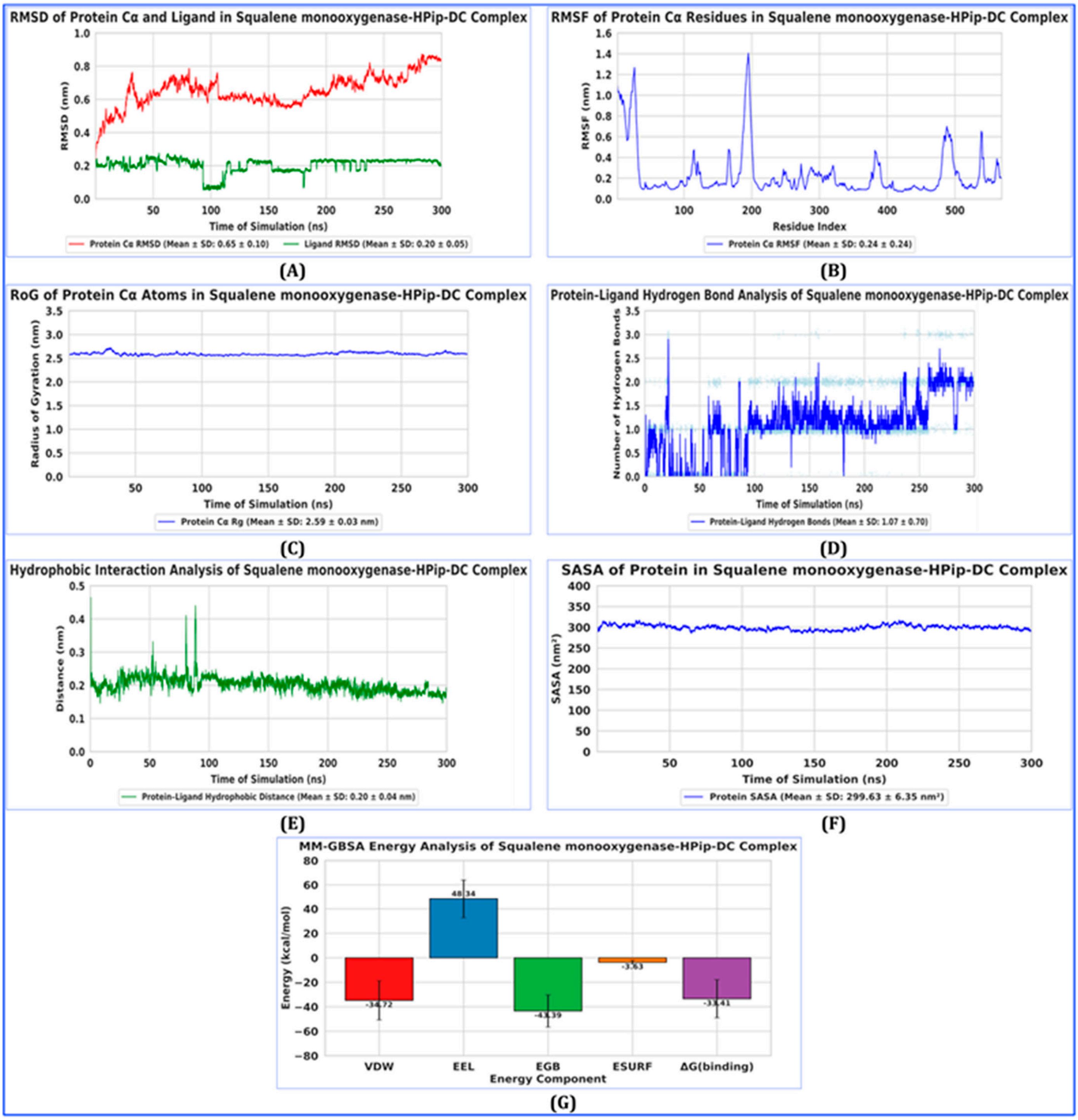

Figure S18. Molecular dynamics and MM-GBSA analyses of HPip-DC bound to the predicted *Leishmania major* squalene monooxygenase model. The panels show (A) protein Cα and ligand RMSD, (B) protein Cα RMSF, (C) radius of gyration of the protein Cα atoms, (D) protein–ligand hydrogen-bond analysis, (E) the representative protein–ligand hydrophobic-contact distance, (F) protein SASA, and (G) the MM-GBSA energy components and total binding-energy estimate. The structural descriptors in panels A–F were evaluated over the 300-ns simulation, whereas the MM-GBSA analysis was performed using 200 evenly distributed frames extracted from the 200–300-ns trajectory segment. The entropy-uncorrected MM-GBSA binding-energy estimate was −33.41 kcal/mol.

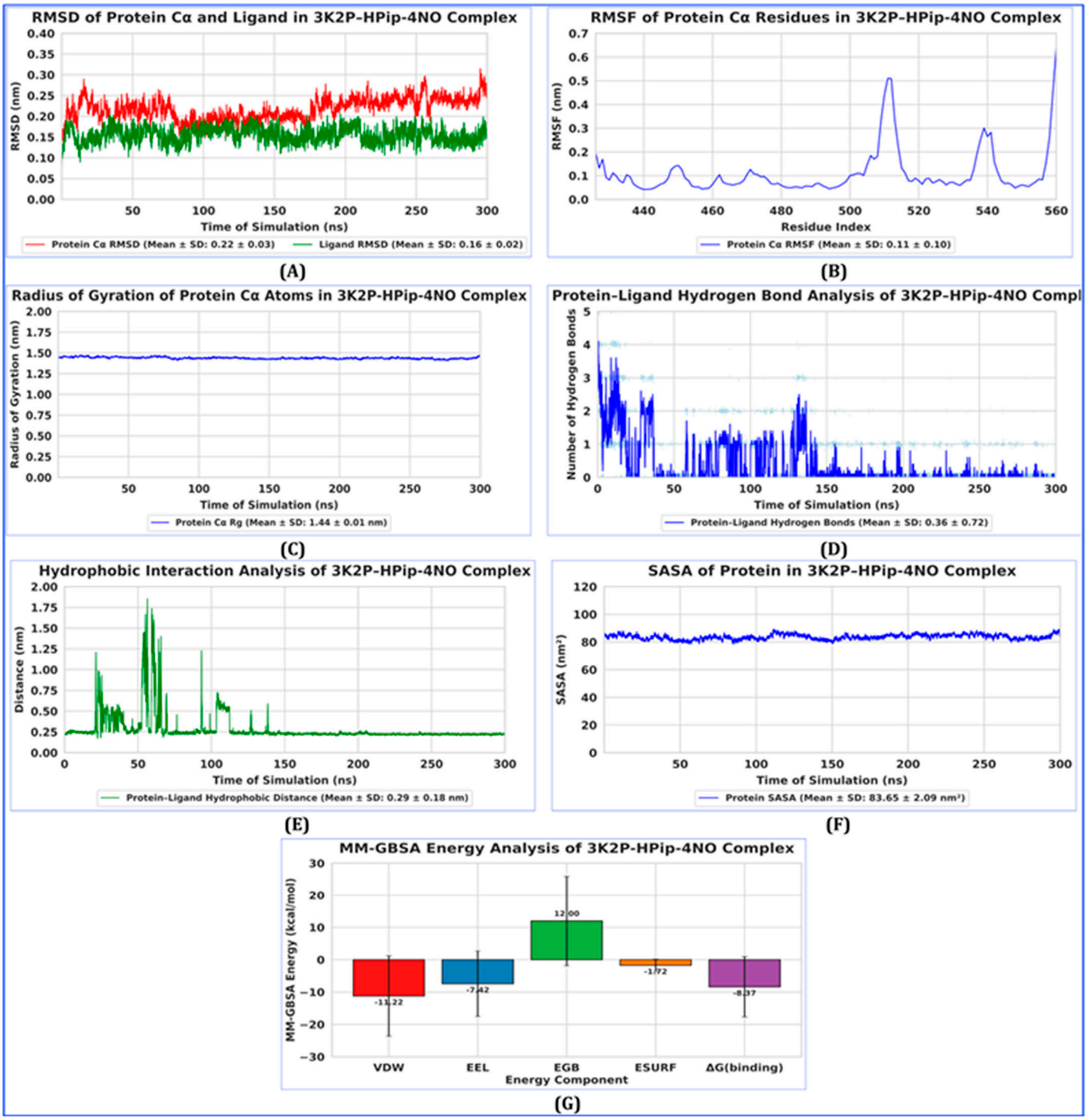

Figure S19. Molecular dynamics and MM-GBSA analyses of HPip-4NO bound to the experimentally resolved HIV-1 reverse transcriptase-associated RNase H domain (PDB ID: 3K2P). The panels show (A) protein Cα and ligand RMSD, (B) protein Cα RMSF, (C) radius of gyration of the protein Cα atoms, (D) protein–ligand hydrogen-bond analysis, (E) the representative protein–ligand hydrophobic-contact distance, (F) protein SASA, and (G) the MM-GBSA energy components and total binding-energy estimate. The structural descriptors in panels A–F were evaluated over the 300-ns simulation, whereas the MM-GBSA analysis was performed using 200 evenly distributed frames extracted from the 200–300-ns trajectory segment. The entropy-uncorrected MM-GBSA binding-energy estimate was −8.37 kcal/mol.

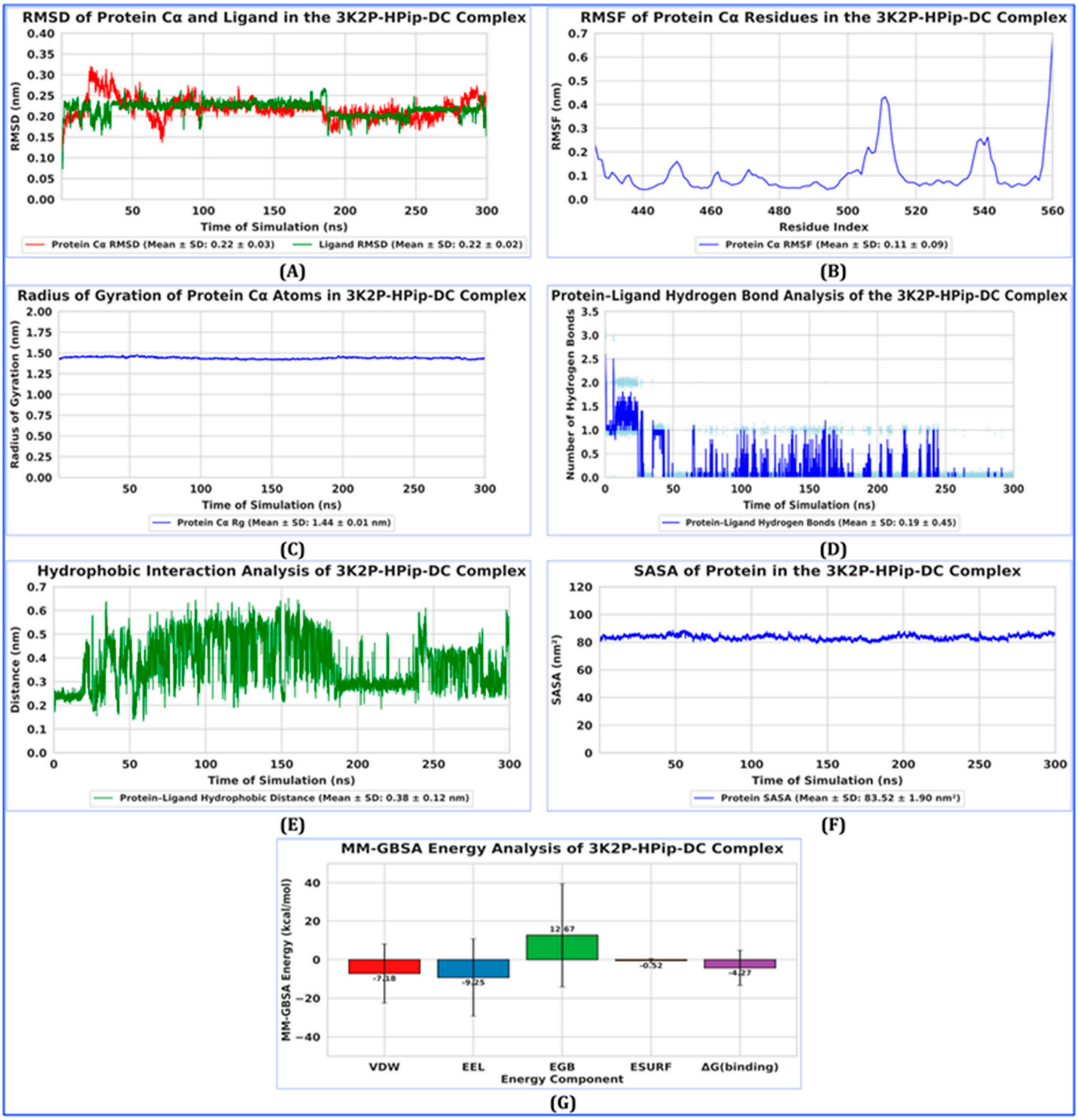

Figure S20. Molecular dynamics and MM-GBSA analyses of HPip-DC bound to experimentally resolved HIV-1 reverse transcriptase-associated RNase H domain (PDB ID: 3K2P). The panels show (A) protein Cα and ligand RMSD, (B) protein Cα RMSF, (C) radius of gyration of the protein Cα atoms, (D) protein–ligand hydrogen-bond analysis, (E) the representative protein–ligand hydrophobic-contact distance, (F) protein SASA, and (G) the MM-GBSA energy components and total binding-energy estimate. The structural descriptors in panels A–F were evaluated over the 300-ns simulation, whereas the MM-GBSA analysis was performed using 200 evenly distributed frames extracted from the 200–300-ns trajectory segment. The entropy-uncorrected MM-GBSA binding-energy estimate was −4.27 kcal/mol.

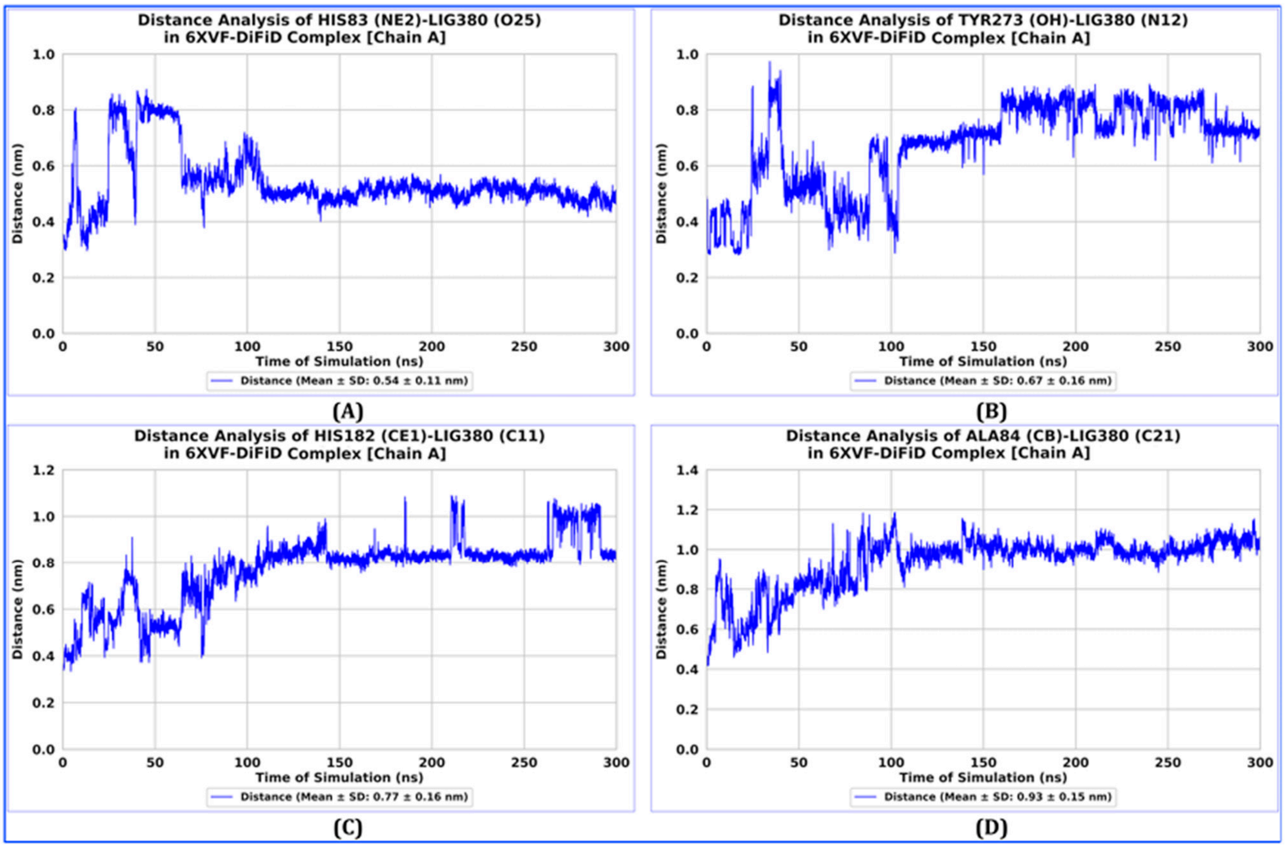

Figure S21. Time-resolved protein–ligand distance analysis of the bovine mitochondrial cytochrome bc_1–DiFiD complex (PDB ID: 6XVF). Distances between DiFiD and selected residues His83, Tyr273, His182, and Ala84 were monitored throughout the 300-ns simulation to assess the persistence and fluctuation of selected contacts.

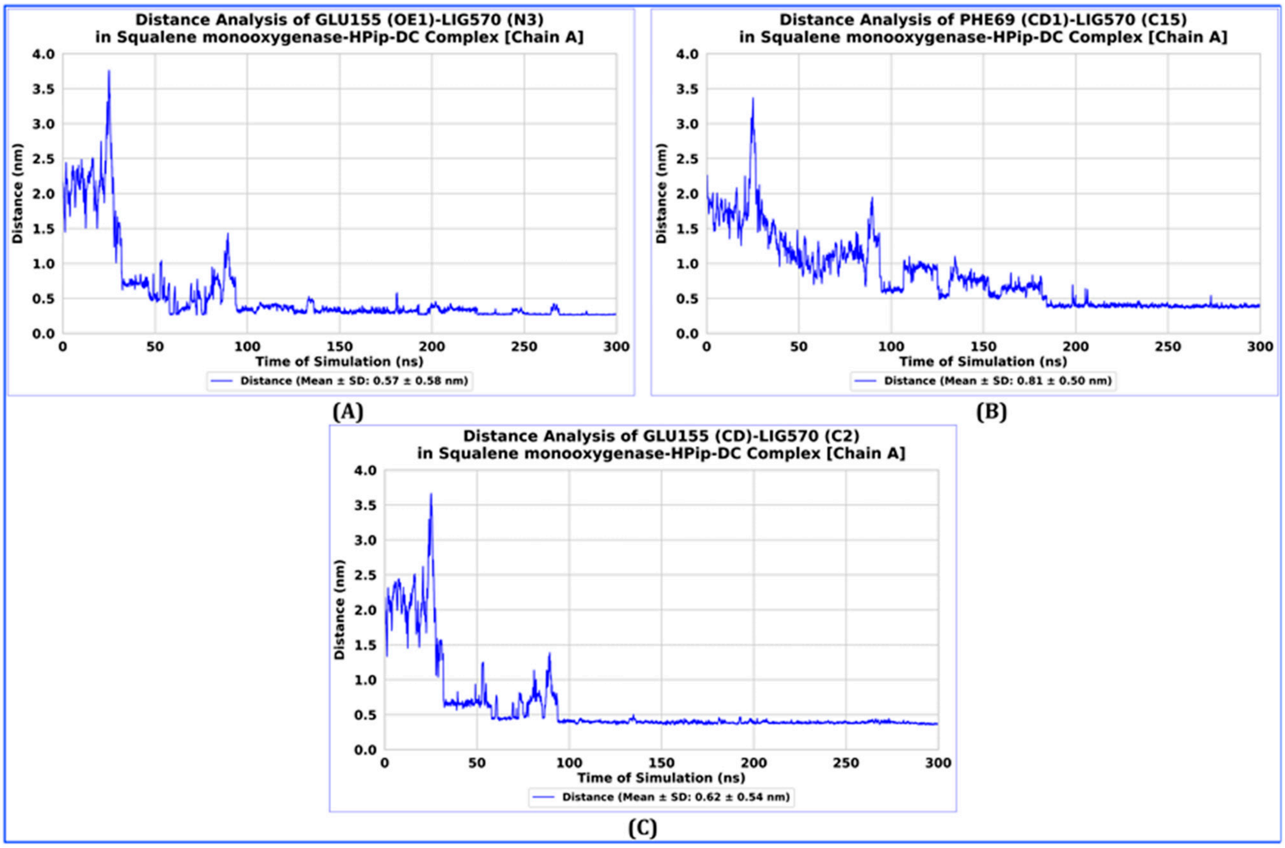

Figure S22. Time-resolved protein–ligand distance analysis of the predicted *L. major* squalene monooxygenase model–HPip-DC complex. Distances between HPip-DC and selected atom pairs involving Glu155 and Phe69 were monitored throughout the 300-ns simulation to evaluate ligand accommodation and contact stability within the predicted binding pocket.

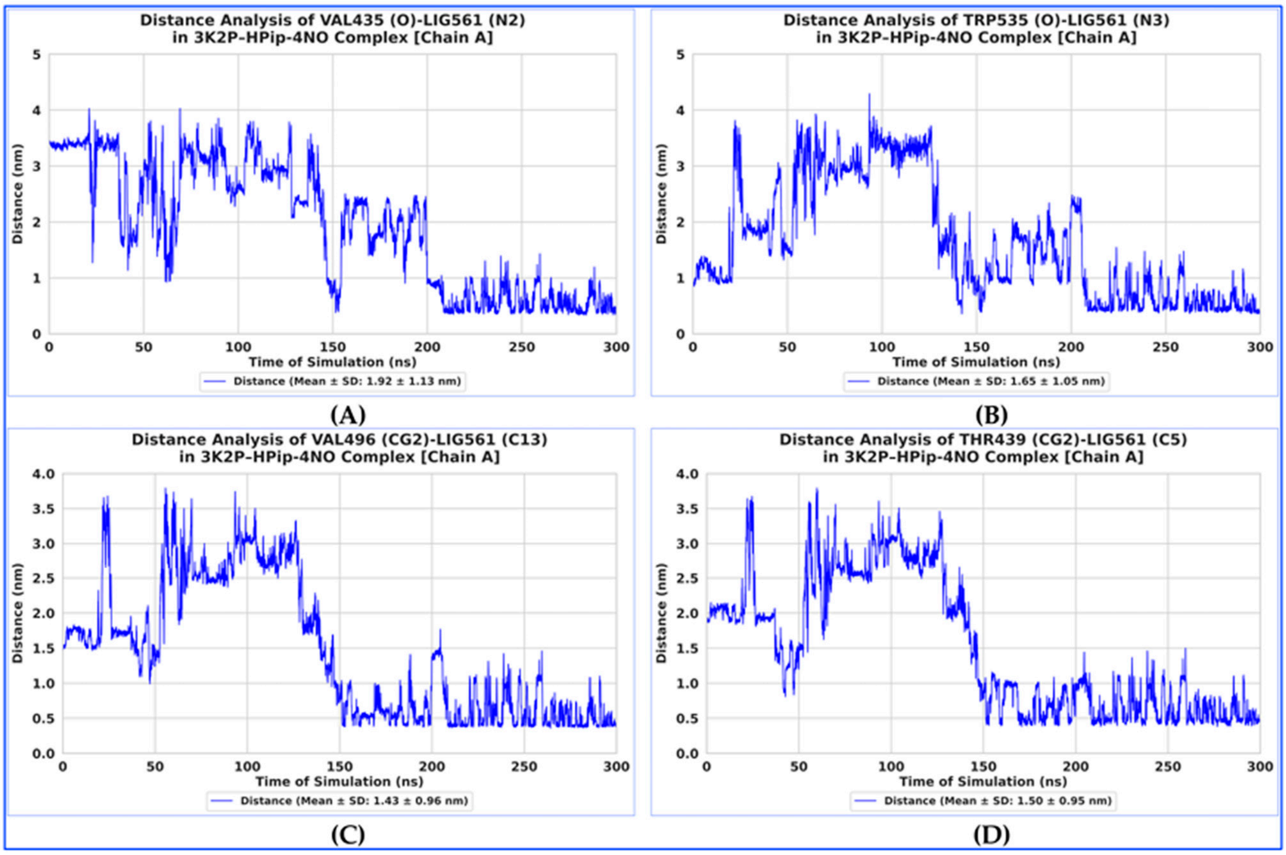

Figure S23. Time-resolved protein–ligand distance analysis of the experimentally resolved HIV-1 RT-associated RNase H–HPip-DC complex (PDB ID: 3K2P). Distances between HPip-4NO and selected residues Val435, Trp535, Val496, and Thr439 were monitored throughout the 300-ns simulation to assess ligand reorientation and stabilization.

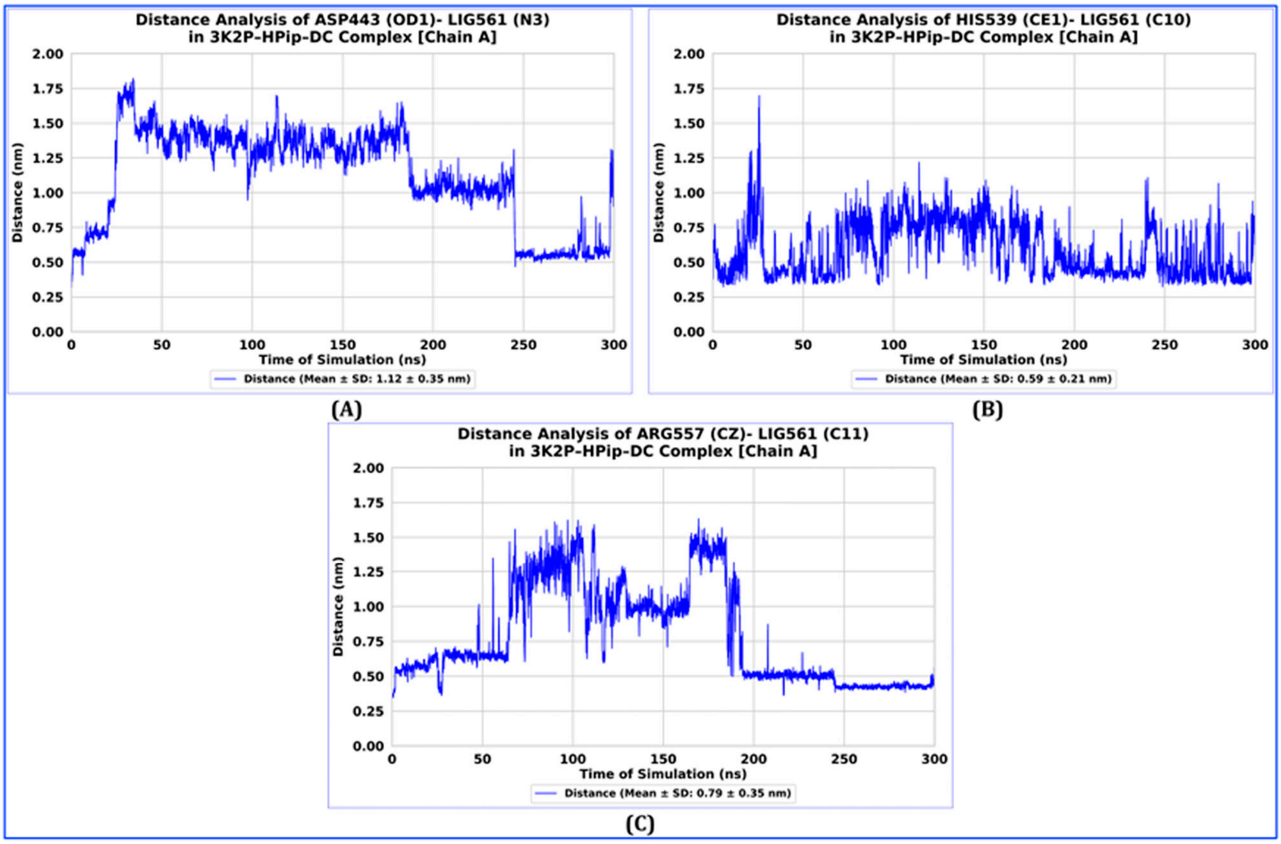

Figure S24. Time-resolved protein–ligand distance analysis of the experimentally resolved HIV-1 RT-associated RNase H–HPip-DC complex (PDB ID: 3K2P). Distances between HPip-DC and selected residues Asp443, His539, and Arg557 were monitored throughout the 300-ns simulation to evaluate the stability and fluctuation of key binding-site contacts.

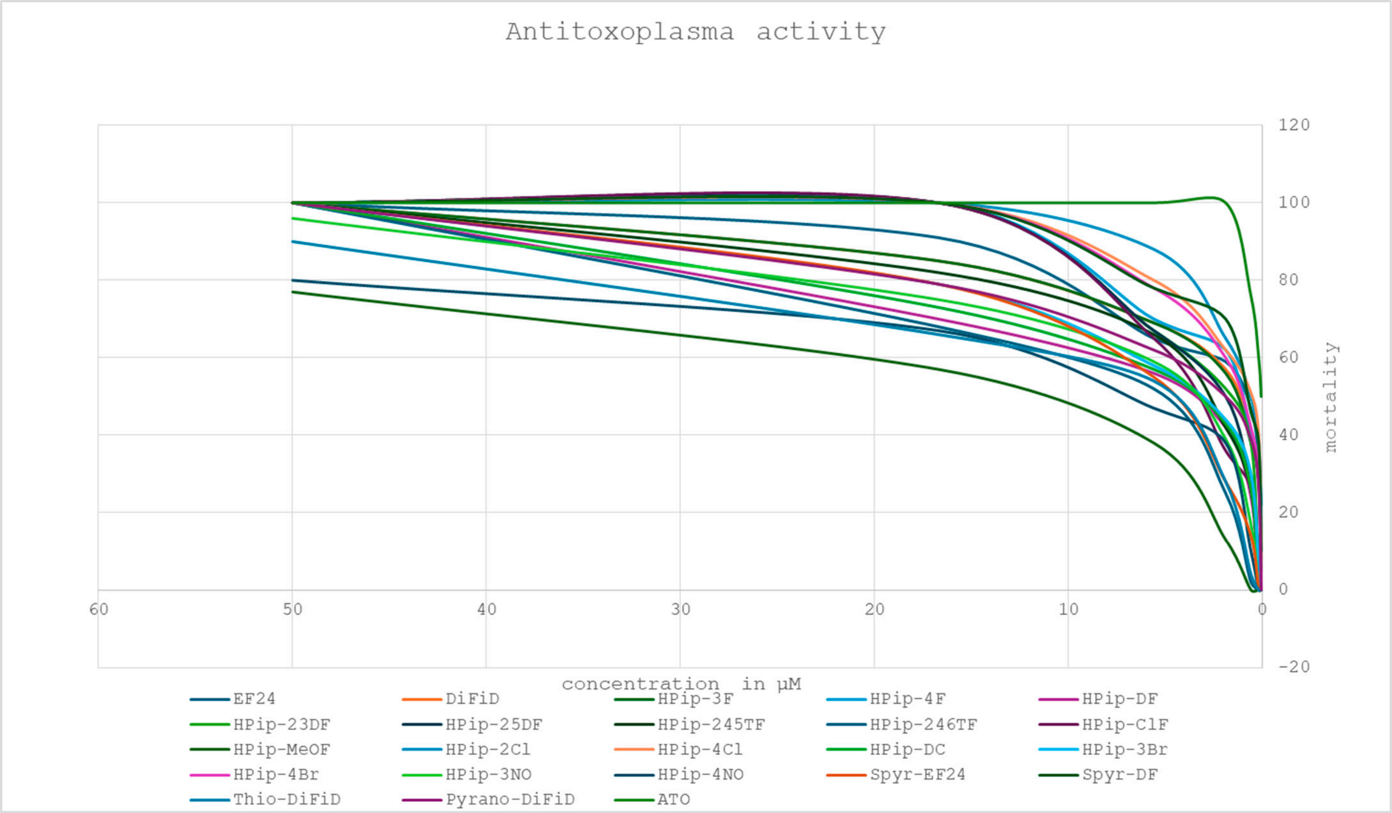

Figure S25. Dose-response curve of the inhibition of *T. gondii* by EF24-analogous curcuminoids.

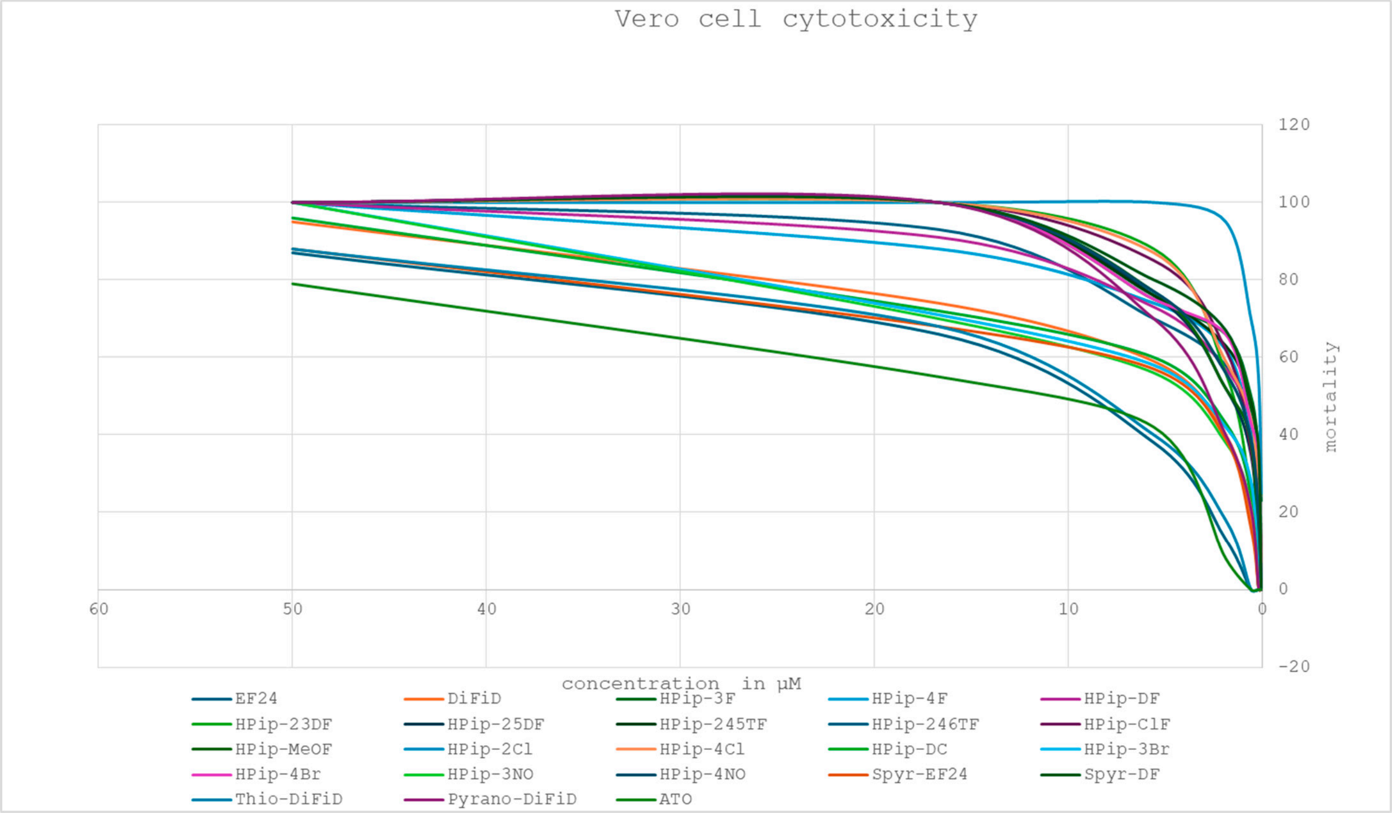

Figure S26. Dose-response curve of the inhibition of Vero cells by EF24-analogous curcuminoids.

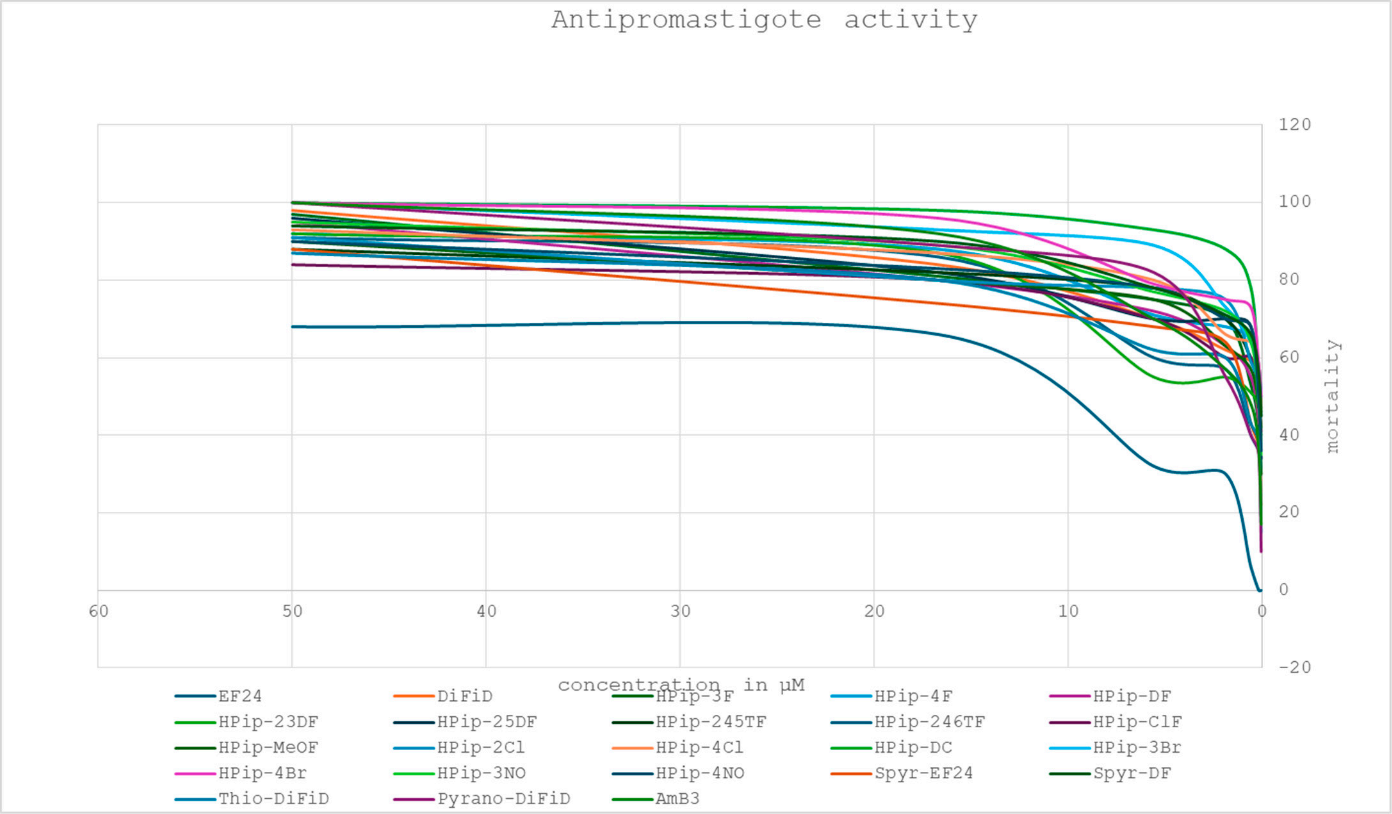

Figure S27. Dose-response curve of the inhibition of *L. major* promastigotes by EF24-analogous curcuminoids.

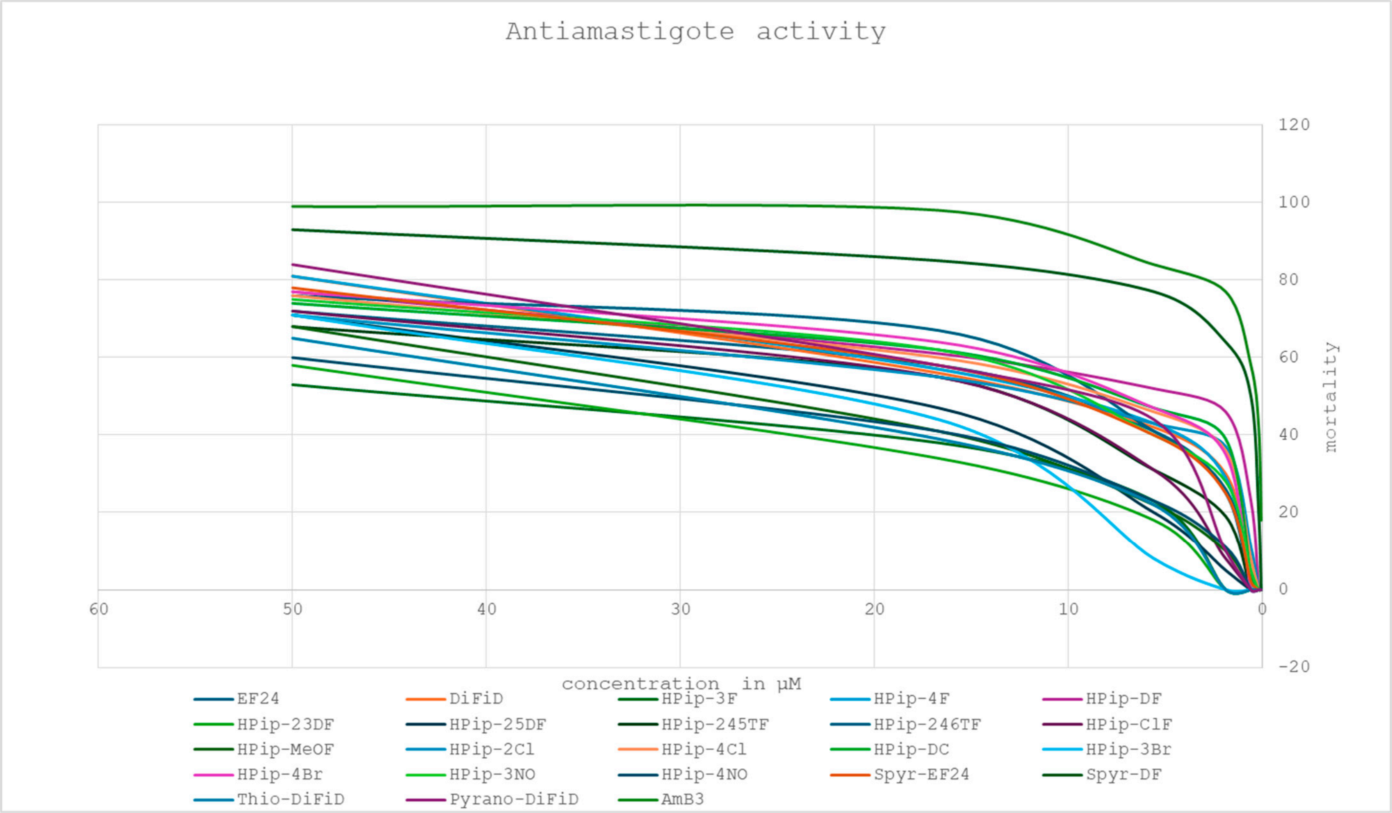

Figure S28. Dose-response curve of the inhibition of *L. major* amastigotes by EF24-analogous curcuminoids.

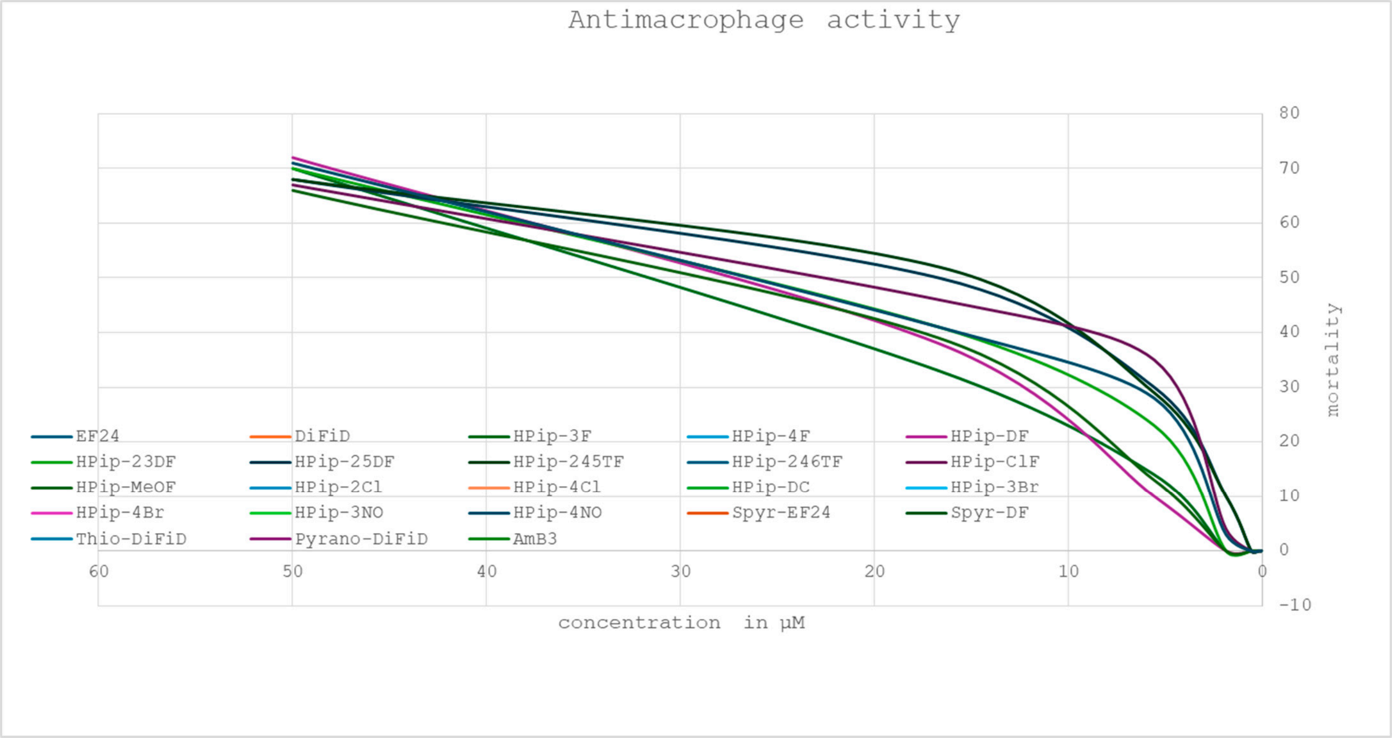

Figure S29. Dose-response curve of the inhibition of murine macrophages by EF24-analogous curcuminoids.

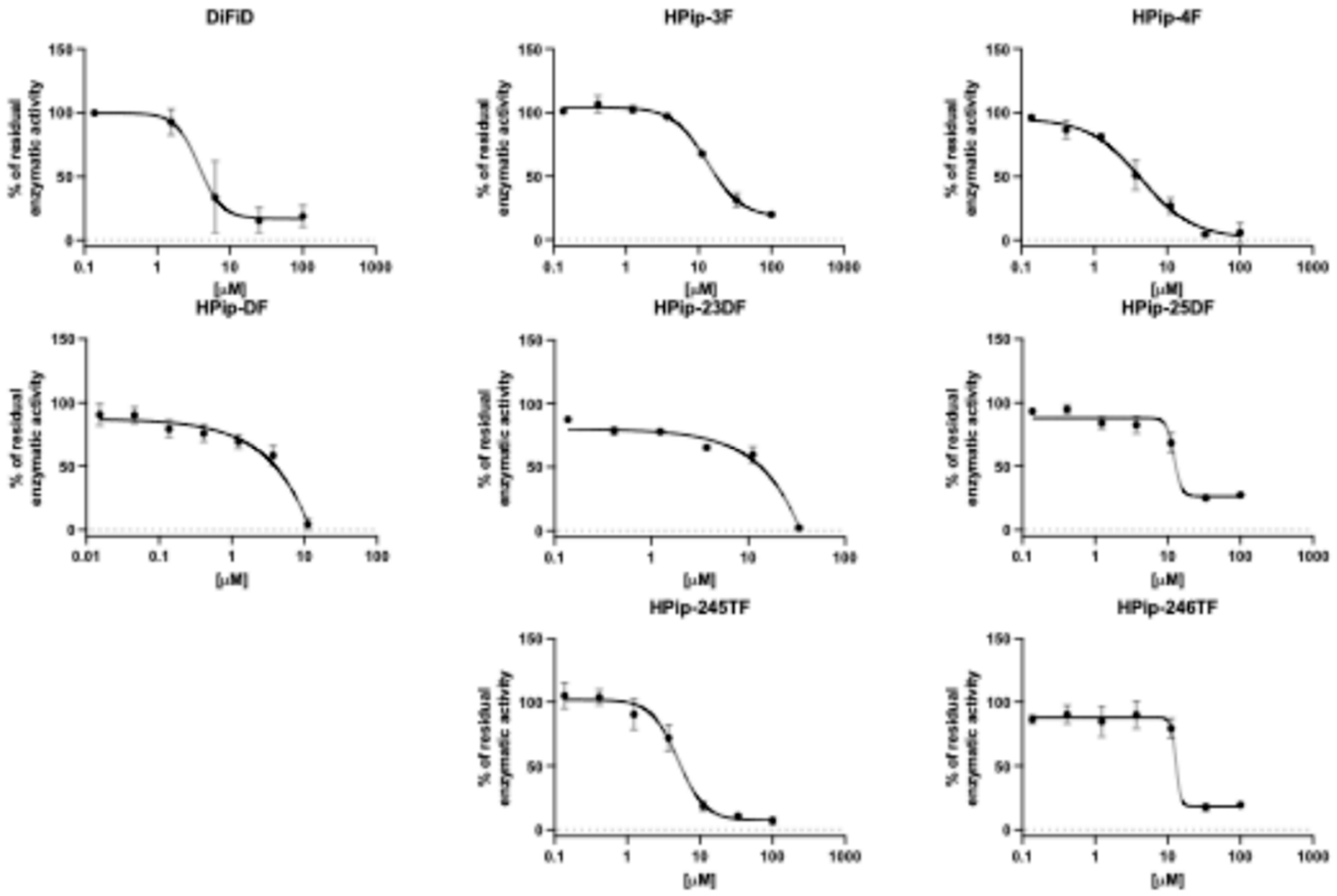

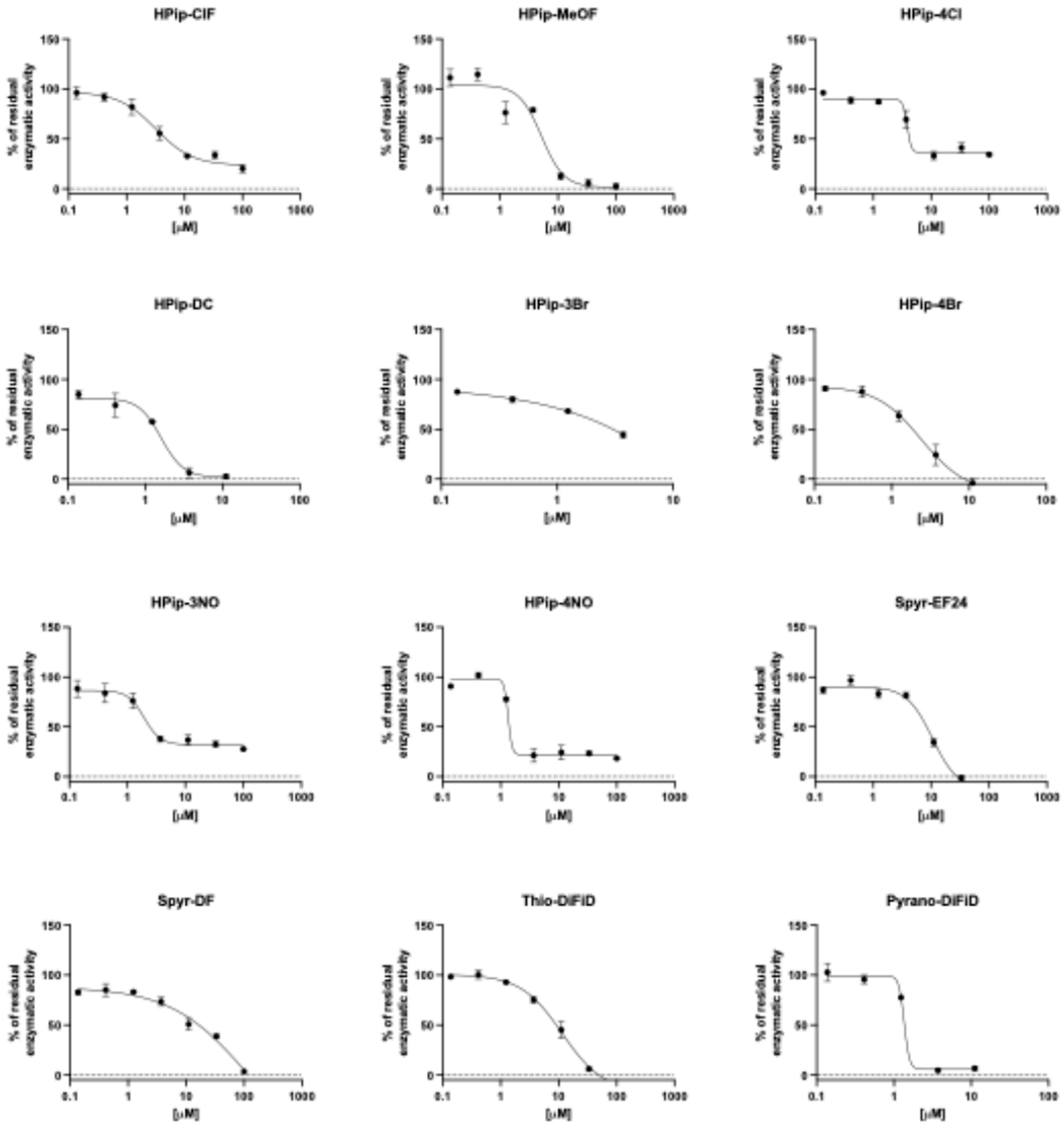

Figure S30. Dose-response curve of the HIV-1 RT associated RNase H inhibition by EF24-analogous curcuminoids.

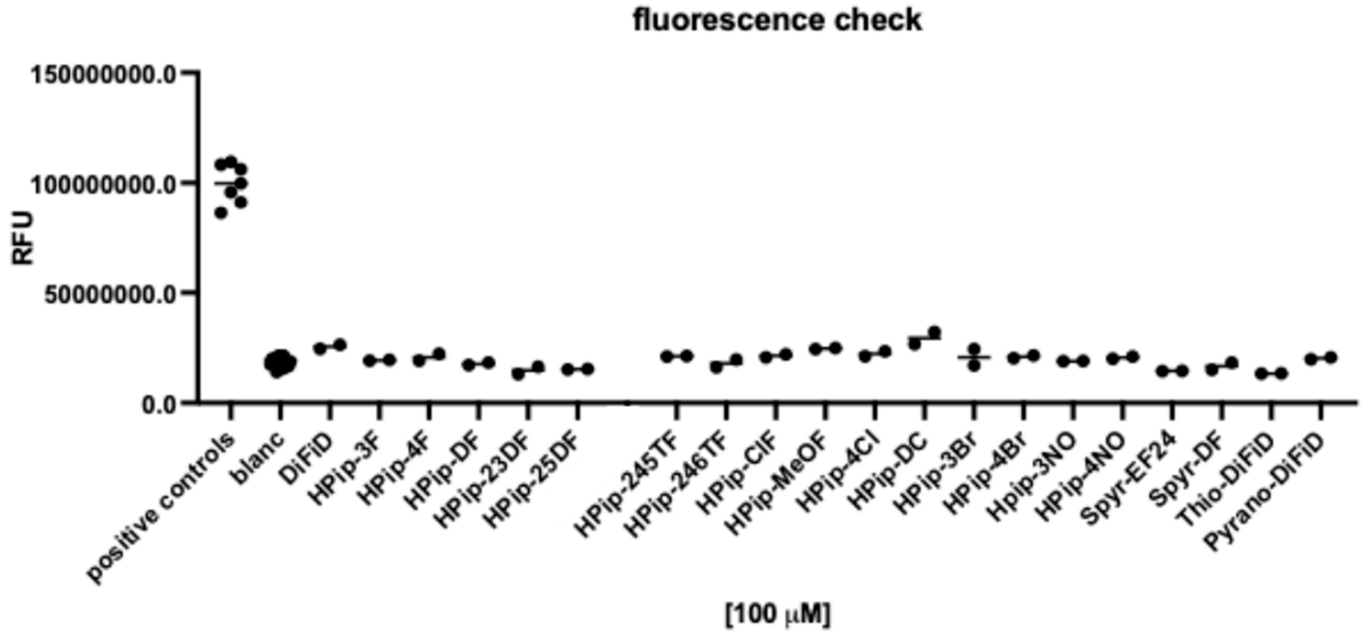

Figure S31. Fluorescence interference check.

Synthesis of Thio-DiFiD

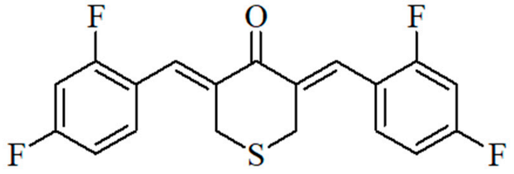

Thio-DiFiD
$C_{19}H_{12}F_4OS$
Mol. Wt.: 364,36

Tetrahydro-4*H*-thiopyran-4-one (116 mg, 1.0 mmol) and 2,4-difluorobenzaldehyde (284 mg, 2.0 mmol) were dissolved in MeOH (10 mL) and NaOH (40 mg) in H_2O (1 mL) was added. The reaction mixture was stirred at room temperature for 1 h. The formed precipitate was collected, washed with MeOH and dried in vacuum. Yield: 226 mg (0.62 mmol, 62%); yellow solid of m.p. 193-194°C; υ_{max}(ATR)/cm^{-1} 3077, 1666, 1615, 1581, 1496, 1480, 1423, 1363, 1266, 1204, 1142, 1135, 1088, 991, 962, 911, 871, 857, 834, 816, 759, 738, 719, 688, 646, 630, 608; ^{1}H NMR (300 MHz, DMSO-d$_6$) δ 3.87 (4 H, s), 7.1-7.2 (2 H, m), 7.3-7.5 (2 H, m), 7.5-7.7 (4 H, m); ^{13}C NMR (75.5 MHz, DMSO-d$_6$) δ 29.3, 104.2-104.8 (m), 111.8-112.1 (m), 118.8-119.0 (m), 126.7-126.8 (m), 132.3-132.4 (m), 136.1, 158.7-158.8 (m), 160.0-160.1 (m), 162.0-162.1 (m), 164.3-164.4 (m), 187.3; *m/z* (%) 364 (100) [M$^+$], 345 (13), 209 (16), 183 (29), 152 (98). Anal. calcd. for $C_{19}H_{12}F_4OS$: C, 62.63%; H, 3.32%; Found: C, 62.75%; H, 3.36.

Analytical data of known test compounds

EF24

^{1}H NMR (300 MHz, $CDCl_3$) δ 4.00 (2 H, s), 4.02 (2 H, s), 7.0-7.4 (8 H, m), 7.84 (2 H, s); Anal. calcd. for $C_{19}H_{15}F_2NO$: C, 73.30%; H, 4.86%; Found: C, 73.39%; H, 4.91%.

DiFiD

^{1}H NMR (300 MHz, $CDCl_3$) δ 3.98 (4 H, s), 6.8-6.9 (4 H, m), 7.2-7.3 (2 H, m), 7.77 (2 H, s); Anal. calcd. for $C_{19}H_{13}F_4NO$: C, 65.71%; H, 3.77%; Found: C, 65.78%; H, 3.80%.

HPip-3F

^{1}H NMR (300 MHz, $CDCl_3$) δ 4.13 (4 H, s), 7.0-7.1 (4 H, m), 7.1-7.2 (2 H, m), 7.2-7.3 (2 H, m), 7.72 (2 H, s); Anal. calcd. for $C_{19}H_{15}F_2NO$: C, 73.30%; H, 4.86%; Found: C, 73.40%; H, 4.88%.

HPip-4F

^{1}H NMR (300 MHz, $CDCl_3$) δ 3.97 (4 H, s), 7.2-7.3 (4 H, m), 7.5-7.6 (6 H, m); Anal. calcd. for $C_{19}H_{15}F_2NO$: C, 73.30%; H, 4.86%; Found: C, 73.38%; H, 4.90%.

HPip-DF

^{1}H NMR (300 MHz, $CDCl_3$) δ 4.10 (4 H, s), 7.0-7.2 (6 H, m), 7.66 (2 H, s); Anal. calcd. for $C_{19}H_{13}F_4NO$: C, 65.71%; H, 3.77%; Found: C, 65.77%; H, 3.80%.

HPip-23DF

^{1}H NMR (300 MHz, DMSO-d_6) δ 2.8-2.9 (1 H, m), 3.88 (4 H, s), 7.2-7.3 (4 H, m), 7.4-7.6 (2 H, m), 7.61 (2 H, s); Anal. calcd. for $C_{19}H_{13}F_4NO$: C, 65.71%; H, 3.77%; Found: C, 65.77%; H, 3.82%.

HPip-25DF

^{1}H NMR (300 MHz, DMSO-d_6) δ 2.7-2.8 (1 H, m), 3.90 (4 H, s), 7.3-7.4 (6 H, m), 7.55 (2 H, s); Anal. calcd. for $C_{19}H_{13}F_4NO$: C, 65.71%; H, 3.77%; Found: C, 65.78%; H, 3.81%.

HPip-245TF

^{1}H NMR (300 MHz, $CDCl_3$/DMSO-d_6) δ 3.98 (4 H, s), 7.1-7.3 (4 H, m), 7.53 (2 H, s); Anal. calcd. for $C_{19}H_{11}F_6NO$: C, 59.54%; H, 2.89%; Found: C, 59.58%; H, 2.93%.

HPip-246TF

^{1}H NMR (300 MHz, DMSO-d_6) δ 3.58 (4 H, s), 7.2-7.4 (6 H, m); HR-MS (ESI) calcd. for $C_{19}H_{12}ONF_6$ [M^+ + H]: 384.08176; Found: 384.08034.

HPip-ClF

^{1}H NMR (300 MHz, DMSO-d_6) δ 3.9-4.0 (4 H, m), 7.4-7.6 (6 H, m), 7.7-7.8 (2 H, m); Anal. calcd. for $C_{19}H_{13}Cl_2F_2NO$: C, 59.97%; H, 3.45%; Found: C, 60.05%; H, 3.48%.

HPip-MeOF

^{1}H NMR (300 MHz, DMSO-d_6) δ 3.89 (6H, s), 3.9-4.0 (4 H, m), 7.2-7.4 (6 H, m), 7.50 (2 H, s); Anal. calcd. for $C_{21}H_{19}F_2NO_3$: C, 67.92%; H, 5.16%; Found: C, 67.99%; H, 5.23%.

HPip-2Cl

^{1}H NMR (300 MHz, DMSO-d$_6$) δ 3.87 (4H, s), 7.4-7.5 (6 H, m), 7.5-7.6 (2 H, m), 7.74 (2 H, s); Anal. calcd. for $C_{19}H_{15}Cl_2NO$: C, 66.29%; H, 4.39%; Found: C, 66.36%; H, 4.44%.

HPip-4Cl

^{1}H NMR (300 MHz, $CDCl_3$) δ 3.96 (4H, s), 7.5-7.6 (10 H, m); Anal. calcd. for $C_{19}H_{15}Cl_2NO$: C, 66.29%; H, 4.39%; Found: C, 66.33%; H, 4.42%.

HPip-DC

^{1}H NMR (300 MHz, DMSO-d$_6$) δ 3.96 (4H, s), 7.3-7.5 (4 H, m), 7.7-7.8 (4 H, m); HR-MS (ESI) calcd. for $C_{19}H_{14}Cl_4{}^{37}ClNO$ [M^+ + H]: 413.97945; Found: 413.97823.

HPip-3Br

^{1}H NMR (300 MHz, DMSO-d$_6$) δ 3.96 (4H, s), 7.4-7.7 (10 H, m); Anal. calcd. for $C_{19}H_{15}Br_2NO$: C, 52.69%; H, 3.49%; Found: C, 52.75%; H, 3.55%.

HPip-4Br

^{1}H NMR (300 MHz, DMSO-d$_6$) δ 3.95 (4H, s), 7.43 (4 H, d, J = 8.5 Hz), 7.53 (2 H, s), 7.65 (4 H, d, J = 8.5 Hz); Anal. calcd. for $C_{19}H_{15}Br_2NO$: C, 52.69%; H, 3.49%; Found: C, 52.77%; H, 3.54%.

HPip-3NO

^{1}H NMR (300 MHz, DMSO-d$_6$) δ 4.04 (4H, s), 7.6-7.8 (4 H, m), 7.9-8.0 (2 H, m), 8.2-8.3 (4 H, m); Anal. calcd. for $C_{19}H_{15}N_3O_5$: C, 62.46%; H, 4.14%; Found: C, 62.56%; H, 4.17%.

HPip-4NO

^{1}H NMR (300 MHz, DMSO-d_6) δ 4.02 (4H, s), 7.66 (2 H, m), 7.76 (4 H, d, J = 8.5 Hz), 8.28 (4 H, d, J = 8.5 Hz); HR-MS (ESI) calcd. for $C_{19}H_{16}N_3O_5$ [M^+ + H]: 366.10845; Found: 366.10723.

Spyr-EF24

^{1}H NMR (300 MHz, $CDCl_3$) δ 3.76 (4H, s), 7.0-7.2 (4 H, m), 7.2-7.4 (4 H, m), 7.76 (2 H, s); Anal. calcd. for $C_{19}H_{14}F_2OS$: C, 69.50%; H, 4.30%; Found: C, 69.56%; H, 4.33%.

Spyr-DF

^{1}H NMR (300 MHz, $CDCl_3$) δ 3.84 (4H, s), 7.0-7.2 (6 H, m), 7.63 (2 H, s); Anal. calcd. for $C_{19}H_{12}F_4OS$: C, 62.63%; H, 3.32%; Found: C, 62.70%; H, 3.38%.

Pyrano-DiFiD

^{1}H NMR (300 MHz, $CDCl_3$) δ 4.74 (4H, s), 6.8-7.0 (4 H, m), 7.1-7.2 (2 H, m), 7.82 (2 H, s); Anal. calcd. for $C_{19}H_{12}F_4O_2$: C, 65.52%; H, 3.47%; Found: C, 65.61%; H, 3.53%.

SpinWorks 4: 12 bebi-030216-ef24

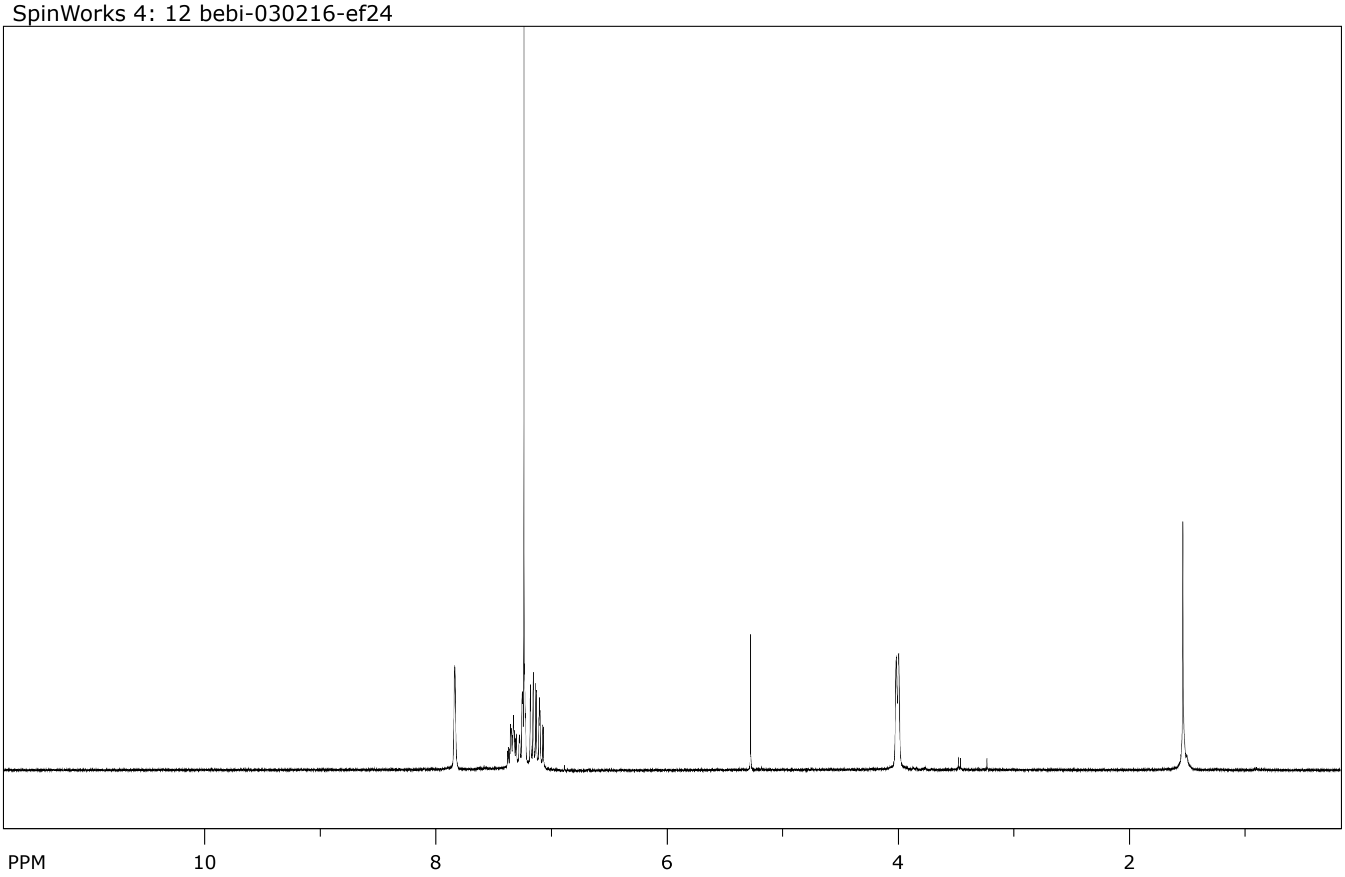

file: C:\Users\bernh\Desktop\10\fid expt: <zg30>
transmitter freq.: 300.131801 MHz
time domain size: 32768 points
width: 4496.40 Hz = 14.9814 ppm = 0.137219 Hz/pt
number of scans: 140

freq. of 0 ppm: 300.130012 MHz
processed size: 32768 complex points
LB: 0.000 GF: 0.0000

Figure S32. ^{1}H NMR spectrum of EF24.

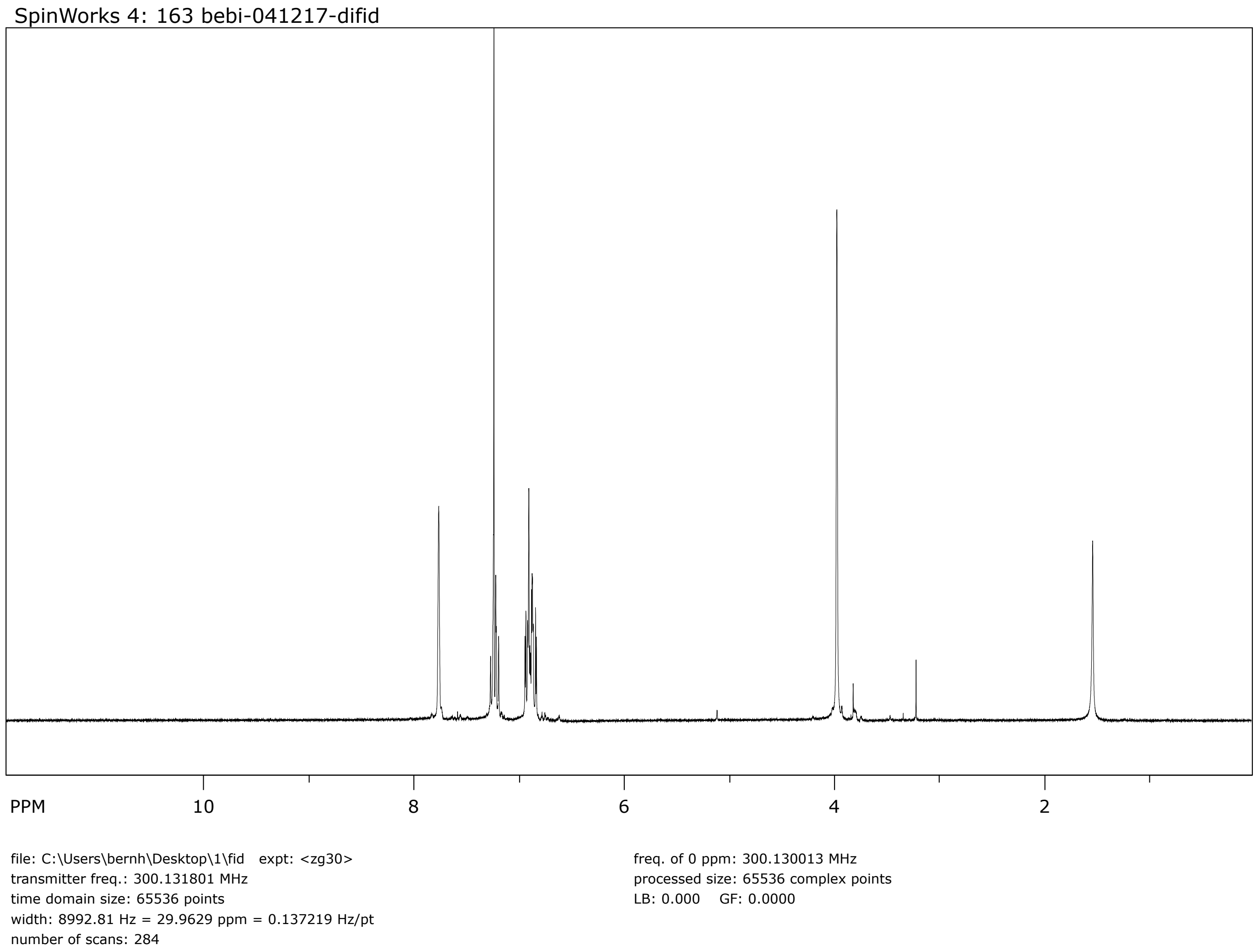

Figure S33. ^{1}H NMR spectrum of DiFiD.

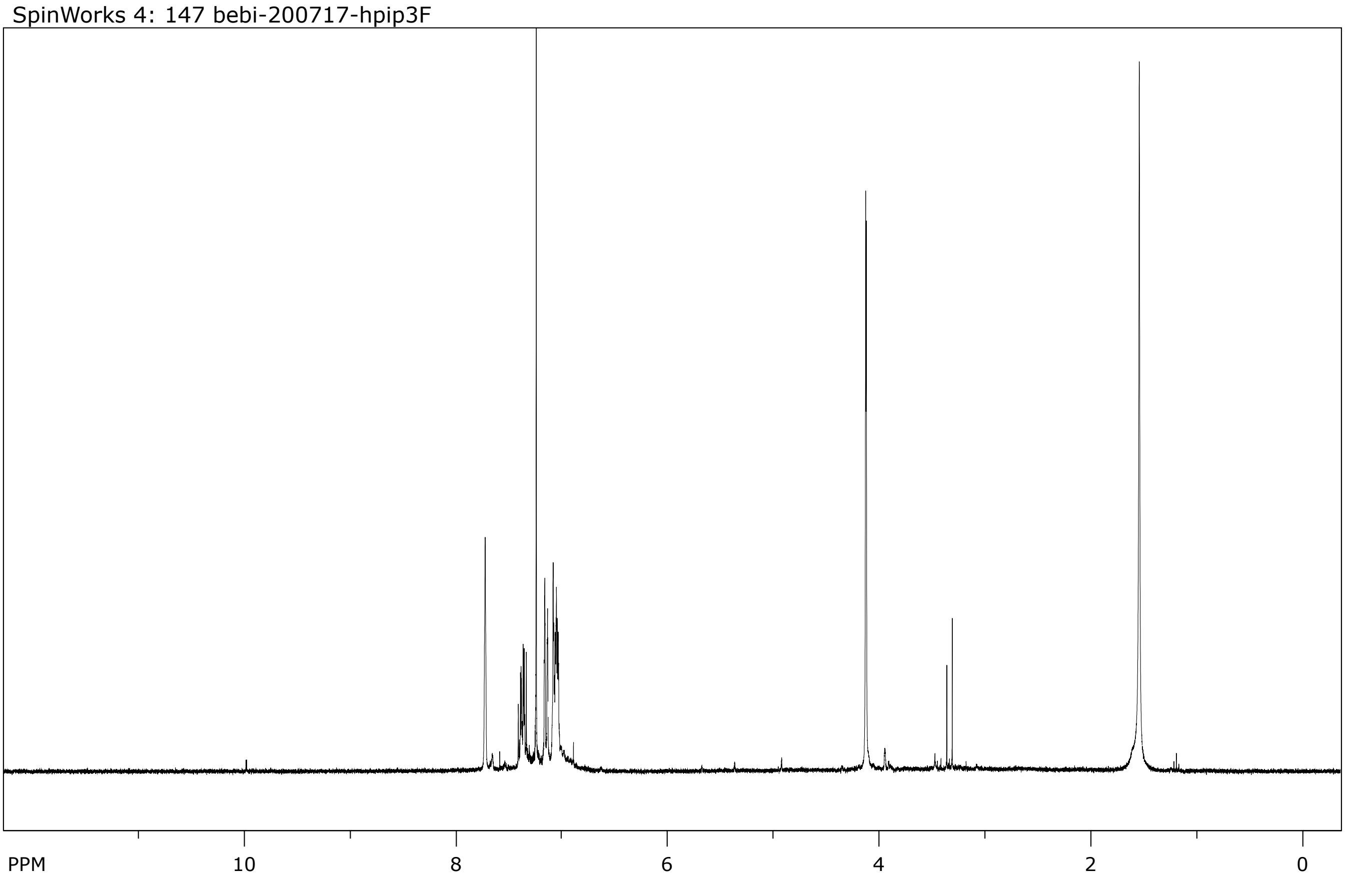

file: C:\Users\bernh\Desktop\1\fid expt: <zg30>
transmitter freq.: 300.131801 MHz
time domain size: 65536 points
width: 8992.81 Hz = 29.9629 ppm = 0.137219 Hz/pt
number of scans: 200

freq. of 0 ppm: 300.130013 MHz
processed size: 65536 complex points
LB: 0.000 GF: 0.0000

Figure S34. ^{1}H NMR spectrum of HPip-3F.

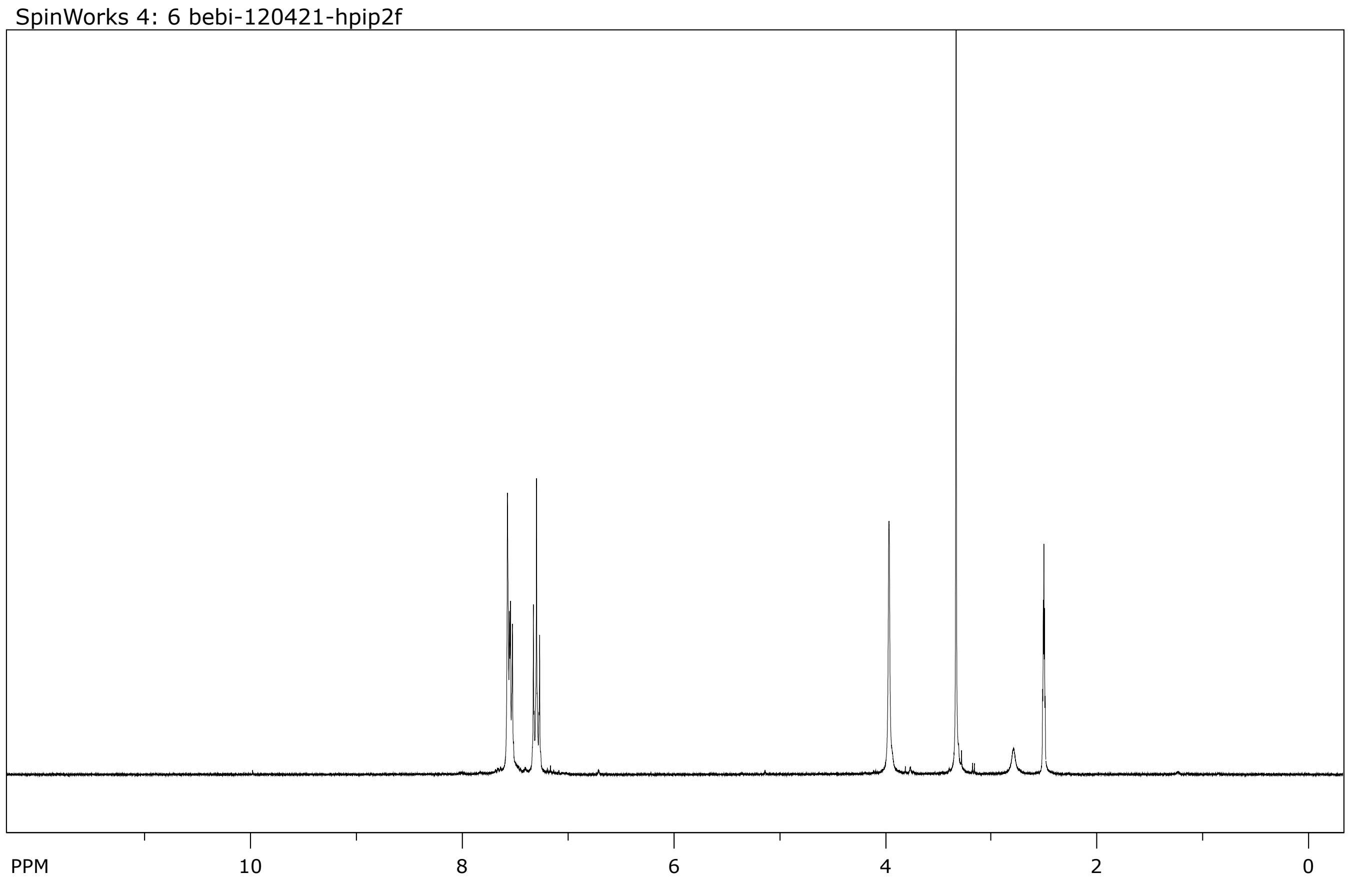

file: C:\Users\bernh\Desktop\1\fid expt: <zg30>
transmitter freq.: 300.131801 MHz
time domain size: 65536 points
width: 8992.81 Hz = 29.9629 ppm = 0.137219 Hz/pt
number of scans: 72

freq. of 0 ppm: 300.130002 MHz
processed size: 65536 complex points
LB: 0.000 GF: 0.0000

Figure S35. ^{1}H NMR spectrum of HPip-4F.

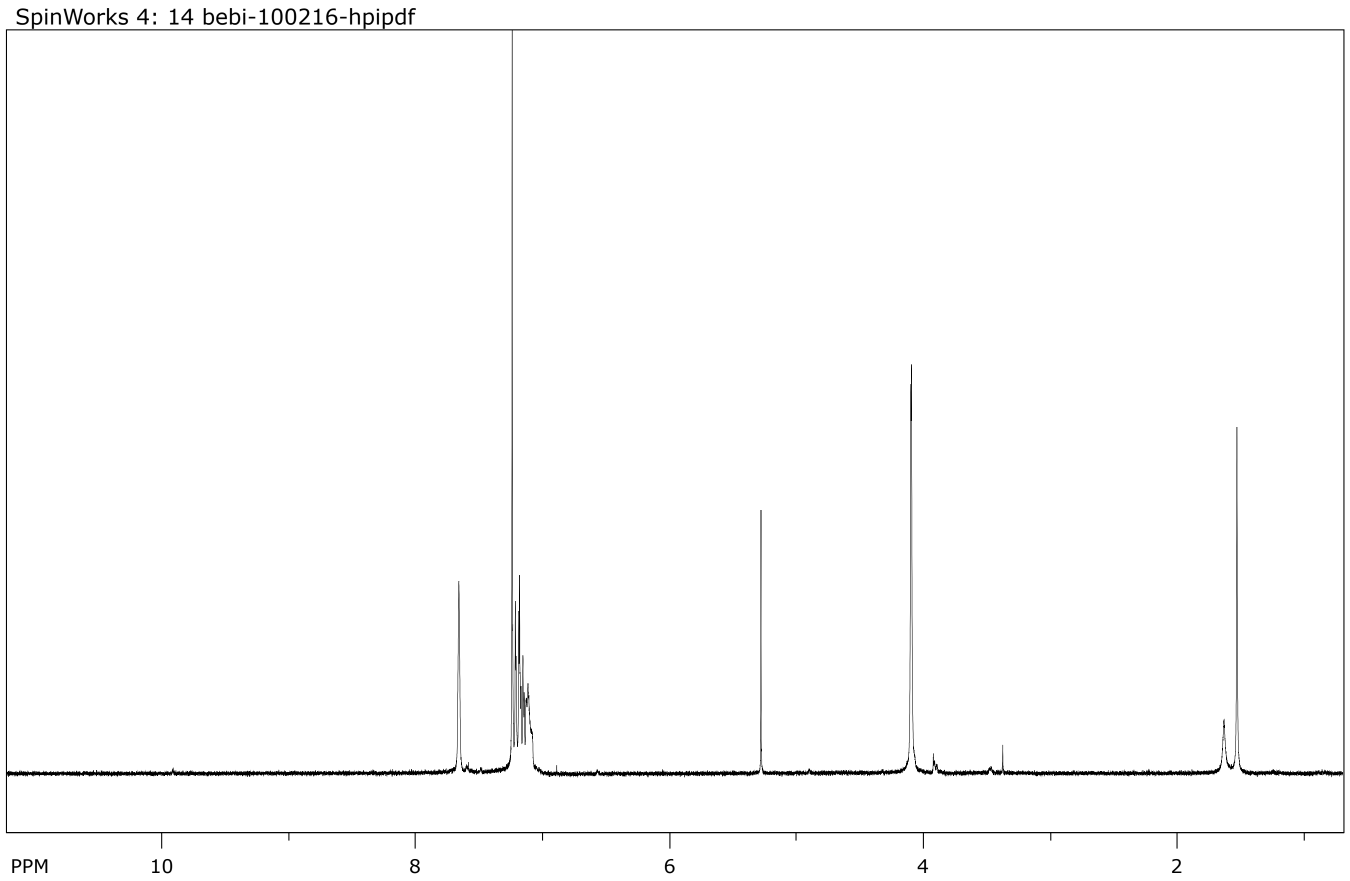

file: C:\Users\bernh\Desktop\1\fid   expt: <zg30>
transmitter freq.: 300.131801 MHz
time domain size: 32768 points
width: 4496.40 Hz = 14.9814 ppm = 0.137219 Hz/pt
number of scans: 100

freq. of 0 ppm: 300.130012 MHz
processed size: 32768 complex points
LB: 0.000   GF: 0.0000

Figure S36. ^{1}H NMR spectrum of HPip-DF.

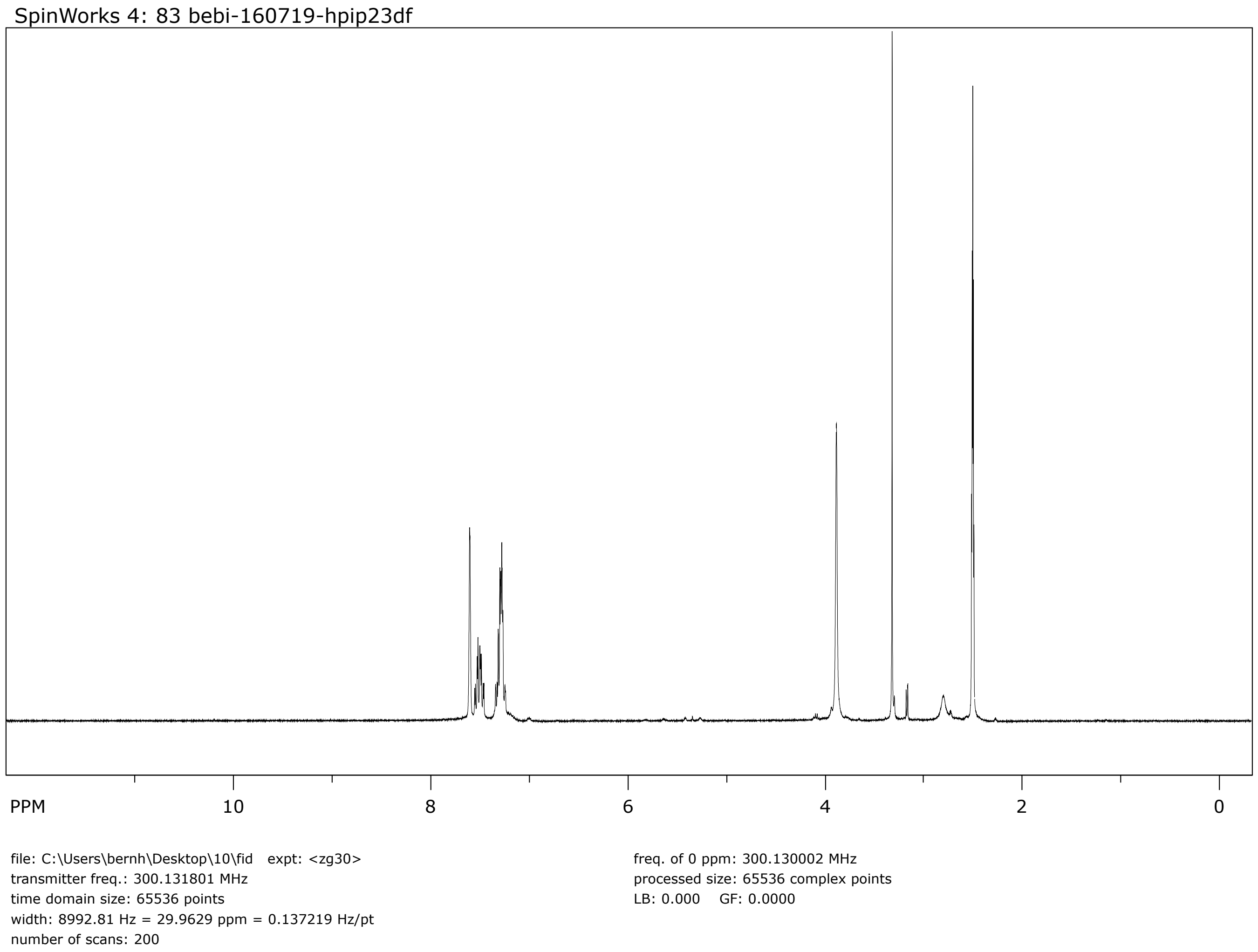

Figure S37. ^{1}H NMR spectrum of HPip-23DF.

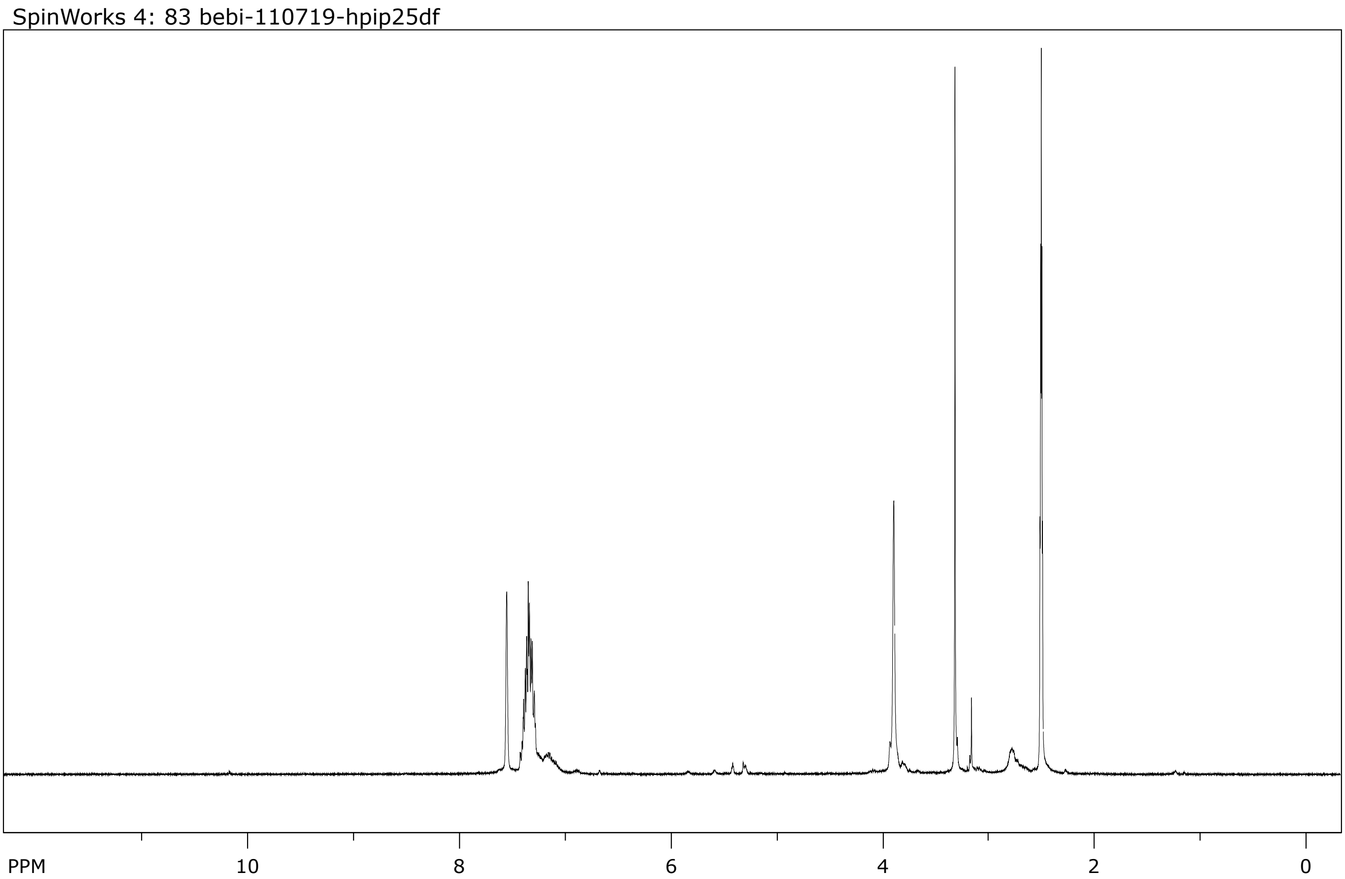

file: C:\Users\bernh\Desktop\10\fid expt: <zg30>
transmitter freq.: 300.131801 MHz
time domain size: 65536 points
width: 8992.81 Hz = 29.9629 ppm = 0.137219 Hz/pt
number of scans: 200

freq. of 0 ppm: 300.130002 MHz
processed size: 65536 complex points
LB: 0.000 GF: 0.0000

Figure S38. ^{1}H NMR spectrum of HPip-25DF.

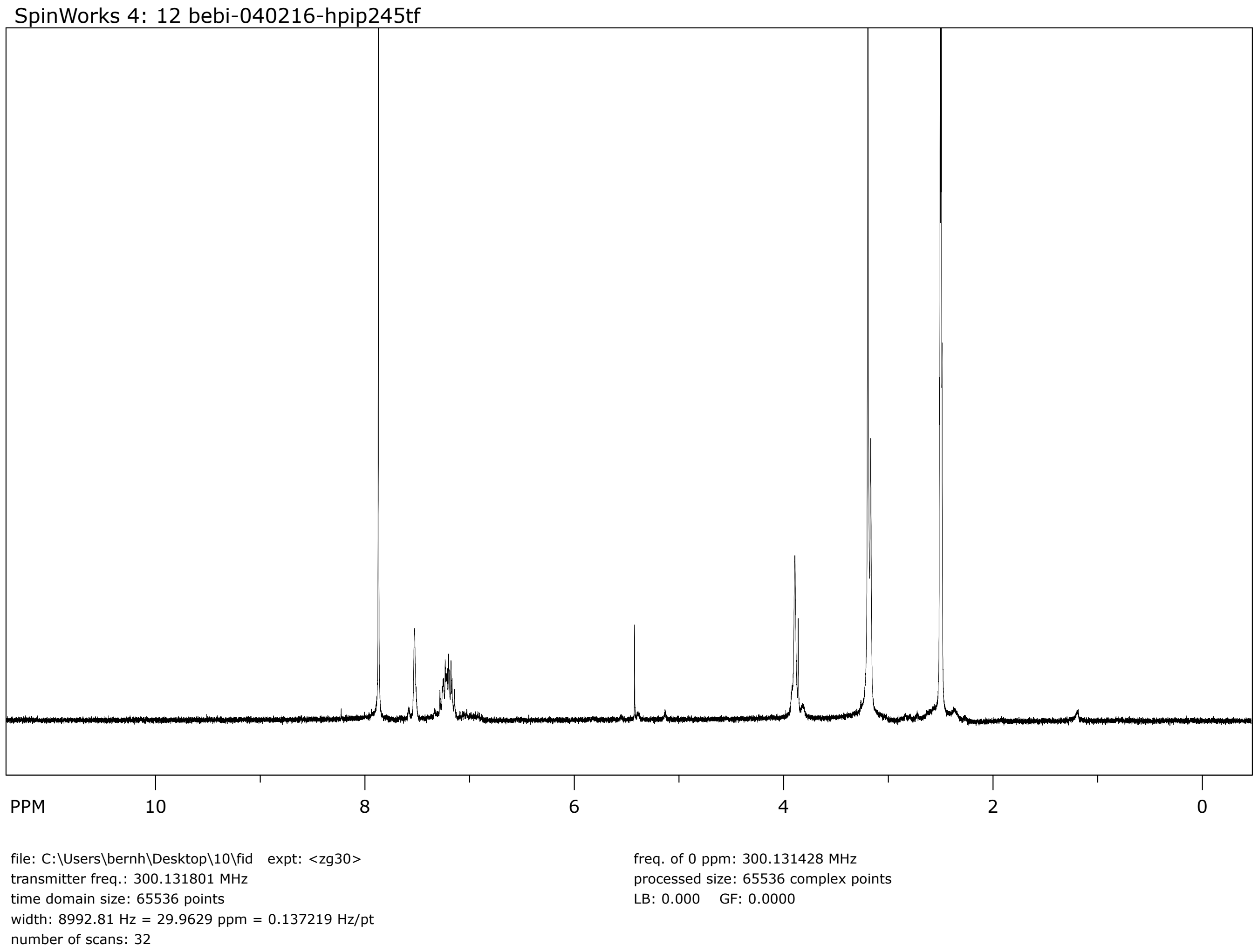

Figure S39. ^{1}H NMR spectrum of HPip-245TF.

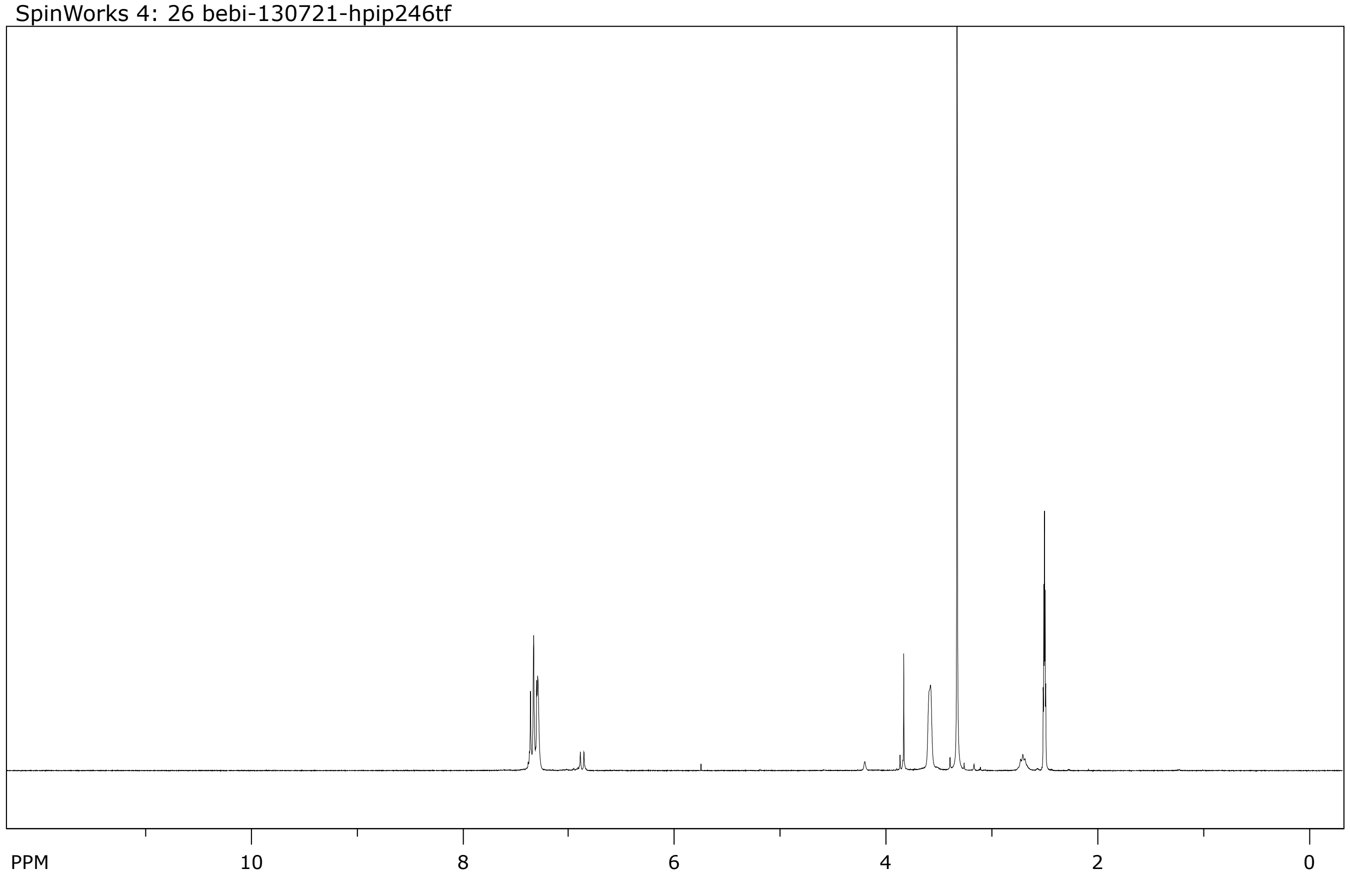

file: C:\Users\bernh\Desktop\10\fid   expt: <zg30>
transmitter freq.: 300.131801 MHz
time domain size: 65536 points
width: 8992.81 Hz = 29.9629 ppm = 0.137219 Hz/pt
number of scans: 32

freq. of 0 ppm: 300.130000 MHz
processed size: 32768 complex points
LB: 0.000   GF: 0.0000

Figure S40. ^{1}H NMR spectrum of HPip-246TF.

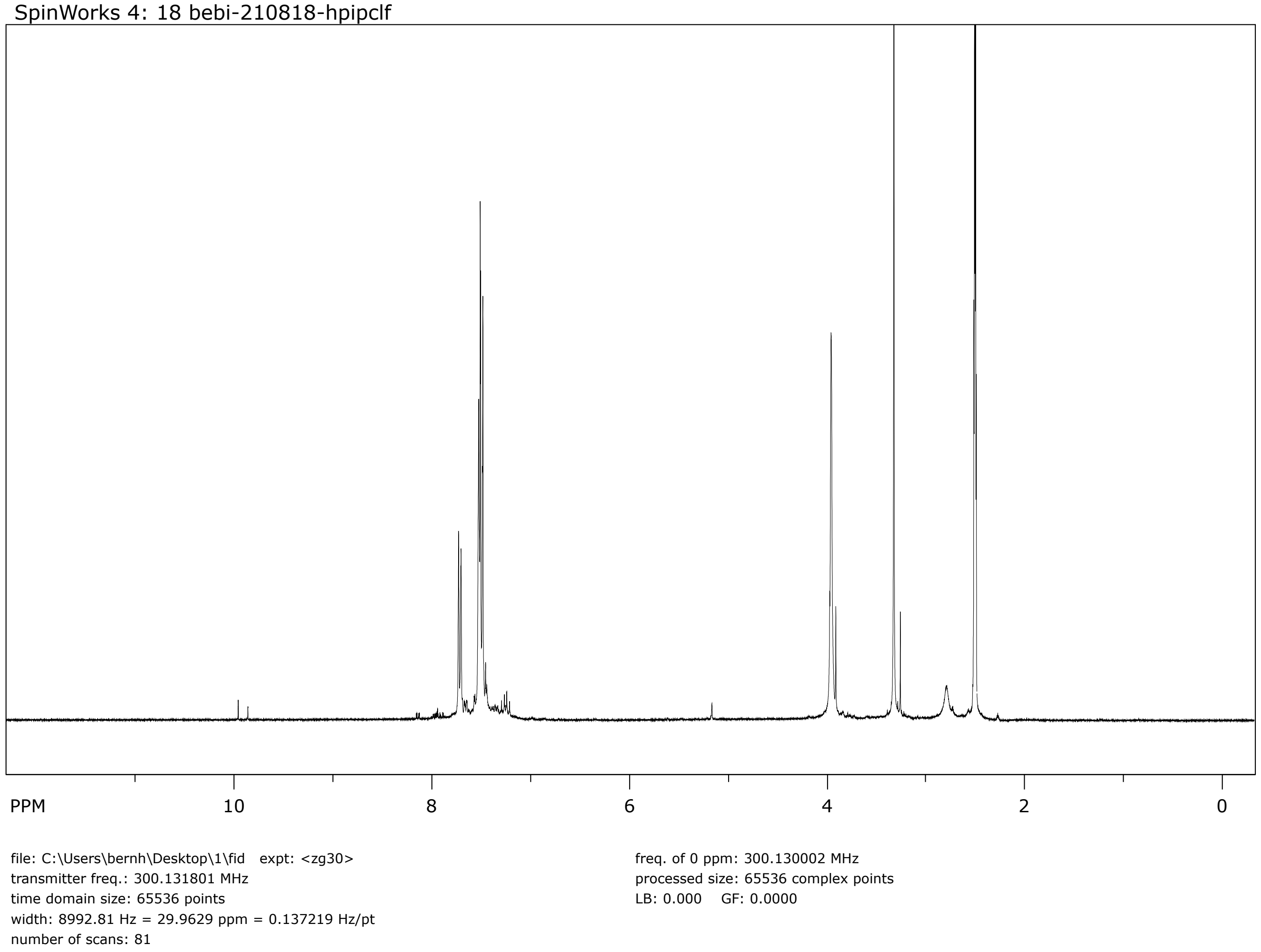

Figure S41. ^{1}H NMR spectrum of HPip-ClF.

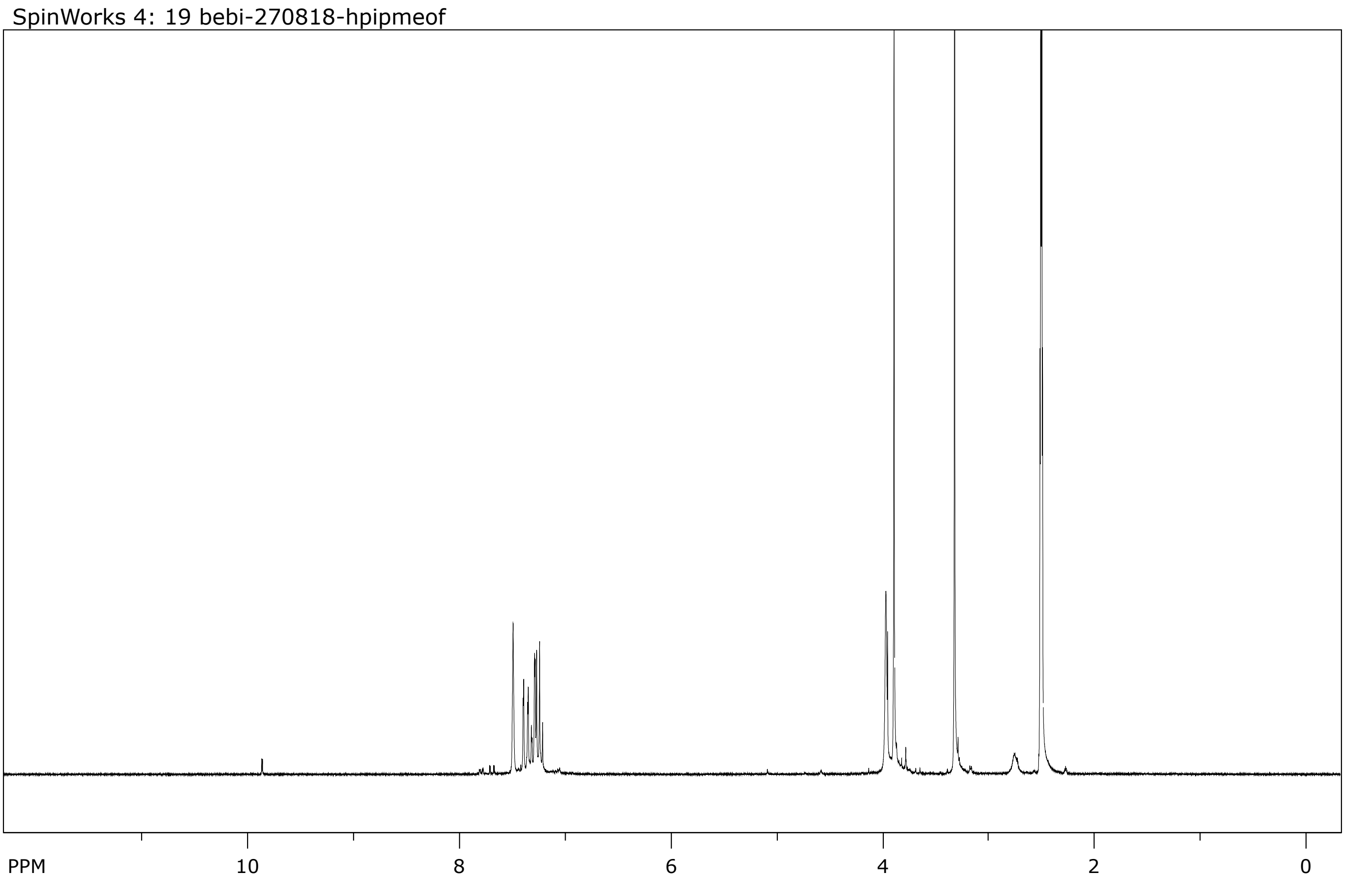

file: C:\Users\bernh\Desktop\10\fid expt: <zg30>
transmitter freq.: 300.131801 MHz
time domain size: 65536 points
width: 8992.81 Hz = 29.9629 ppm = 0.137219 Hz/pt
number of scans: 104

freq. of 0 ppm: 300.130002 MHz
processed size: 65536 complex points
LB: 0.000 GF: 0.0000

Figure S42. ^{1}H NMR spectrum of HPip-MeOF.

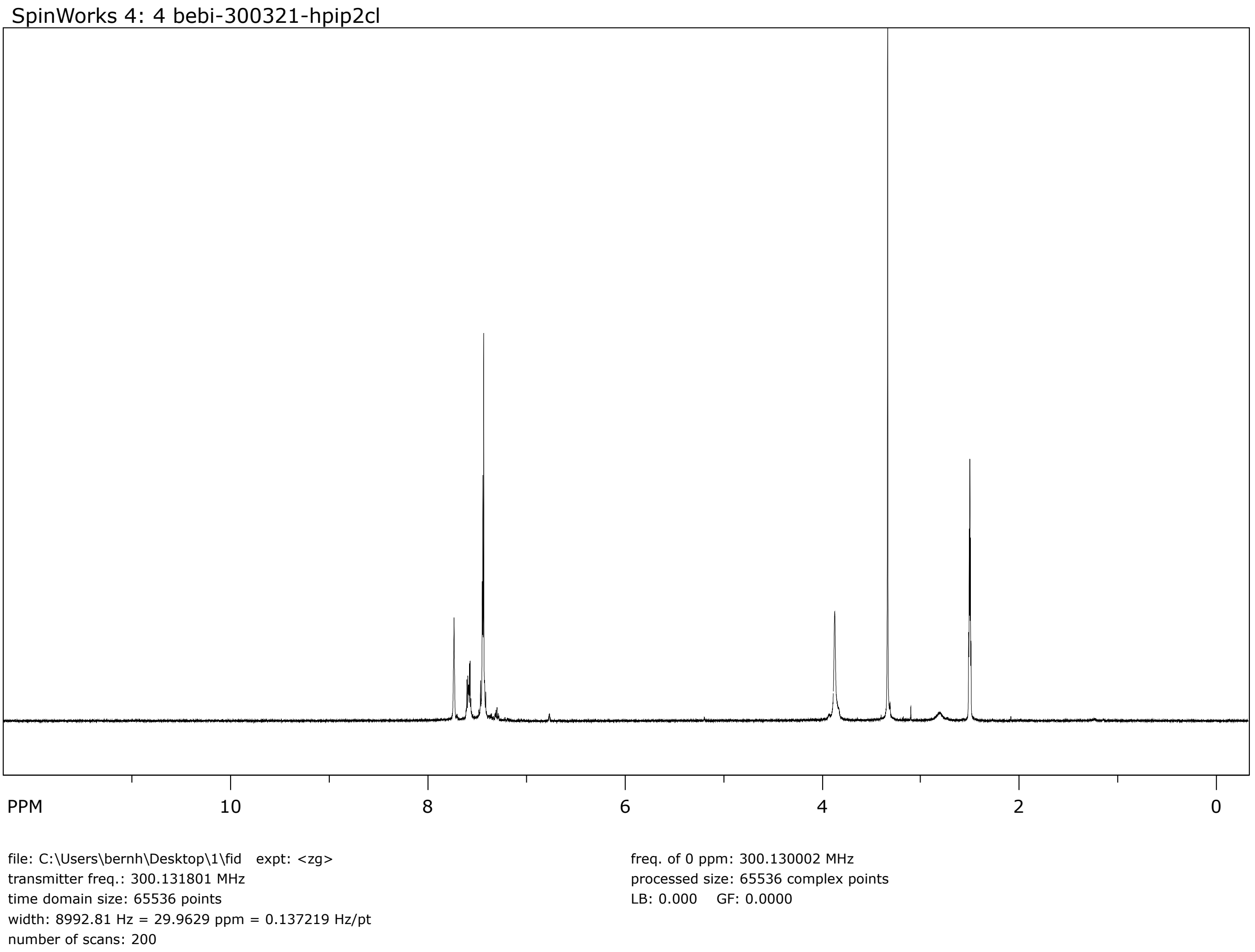

Figure S43. ^{1}H NMR spectrum of HPip-2Cl.

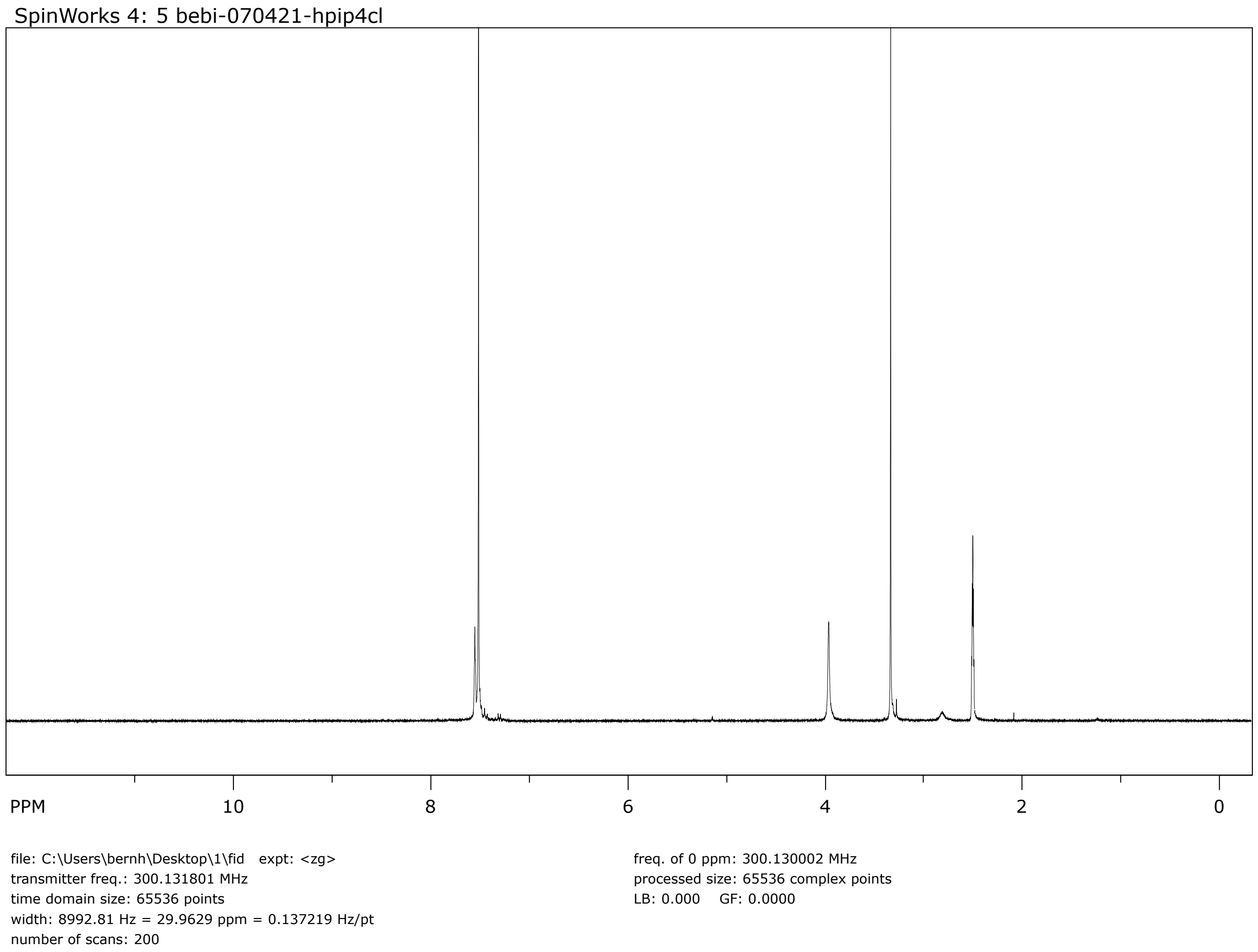

Figure S44. ^{1}H NMR spectrum of HPip-4Cl.

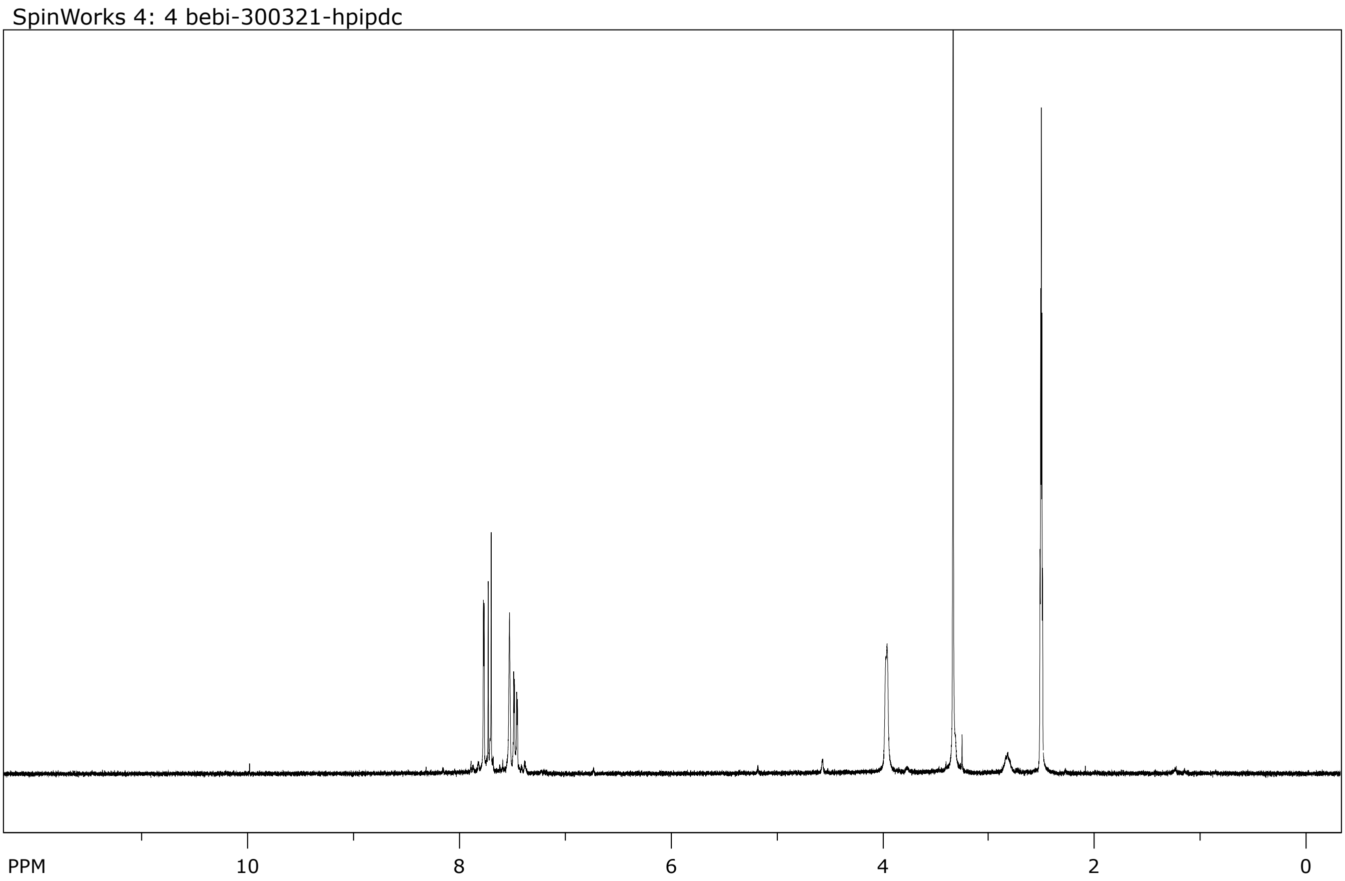

file: C:\Users\bernh\Desktop\1\fid   expt: <zg>
transmitter freq.: 300.131801 MHz
time domain size: 65536 points
width: 8992.81 Hz = 29.9629 ppm = 0.137219 Hz/pt
number of scans: 300

freq. of 0 ppm: 300.130002 MHz
processed size: 65536 complex points
LB: 0.000   GF: 0.0000

Figure S45. ^{1}H NMR spectrum of HPip-DC.

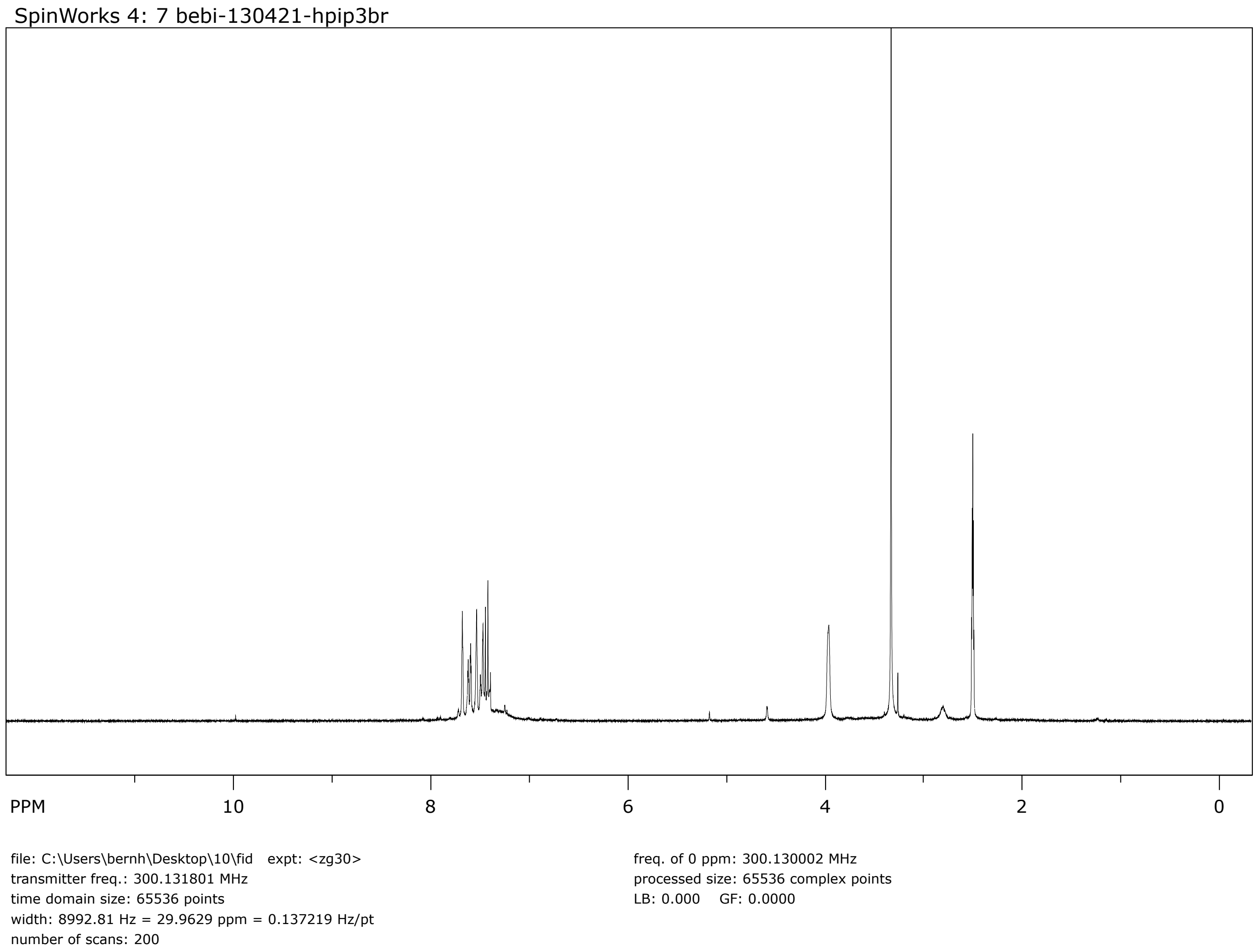

Figure S46. ^{1}H NMR spectrum of HPip-3Br.

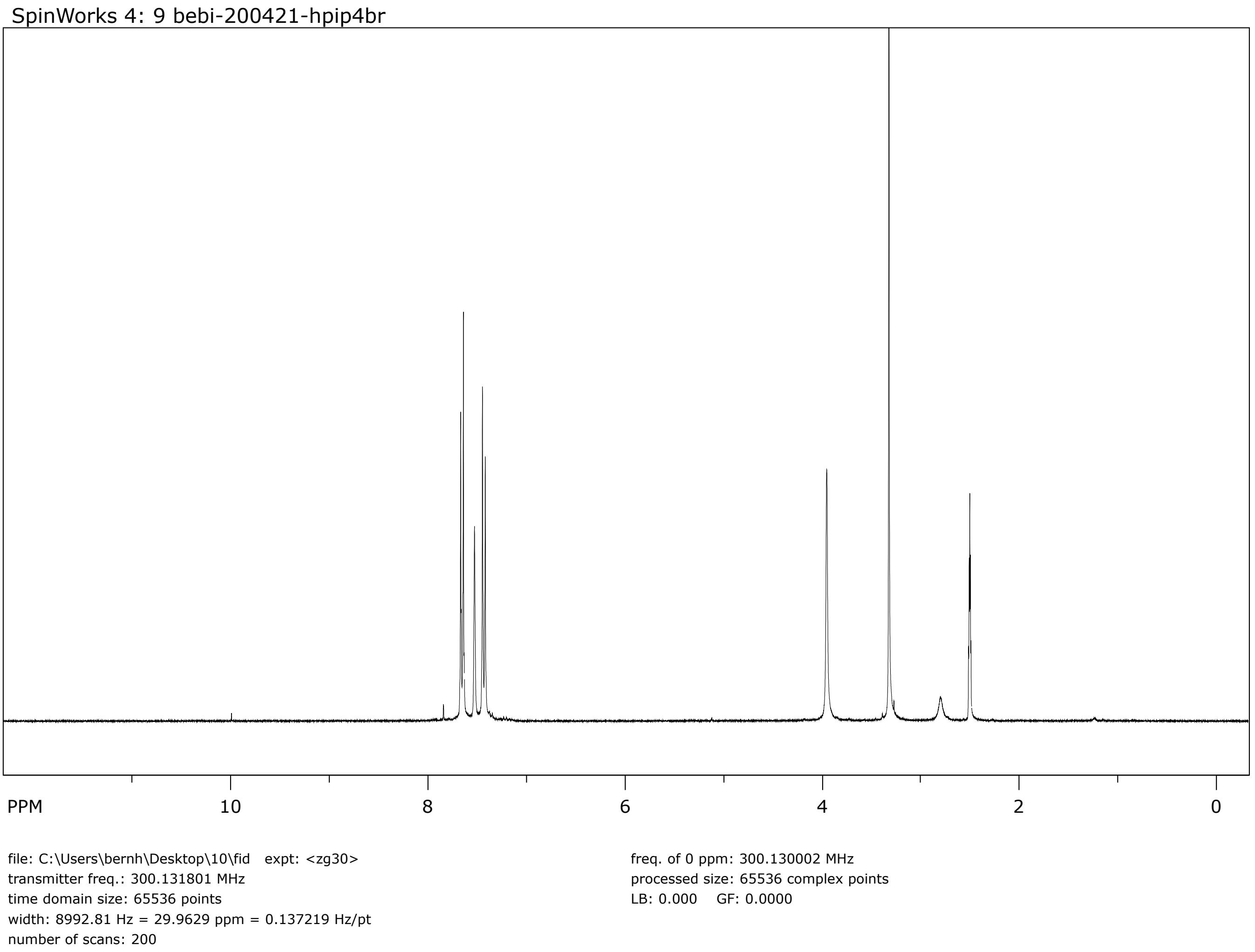

Figure S47. ^{1}H NMR spectrum of HPip-4Br.

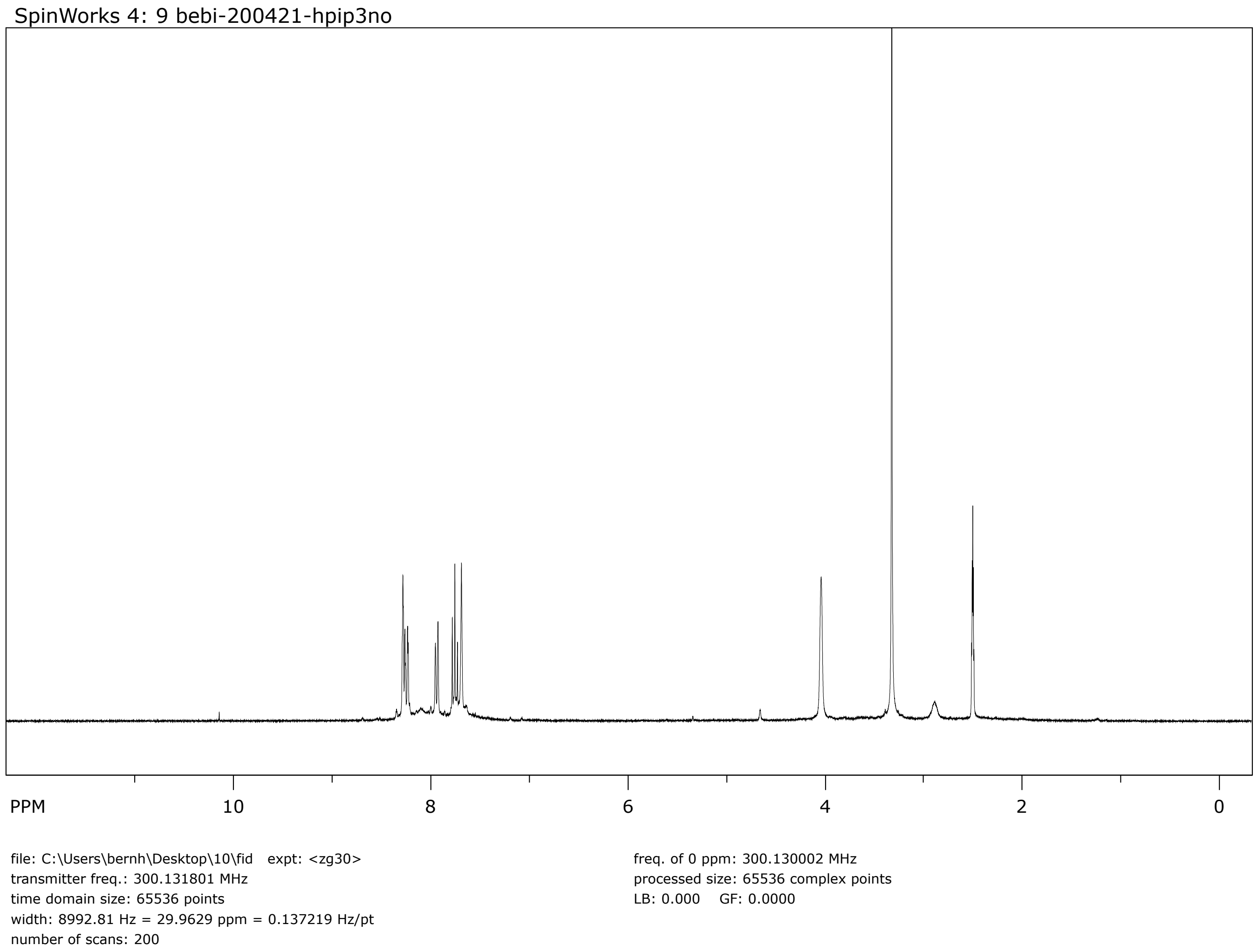

Figure S48. ^{1}H NMR spectrum of HPip-3NO.

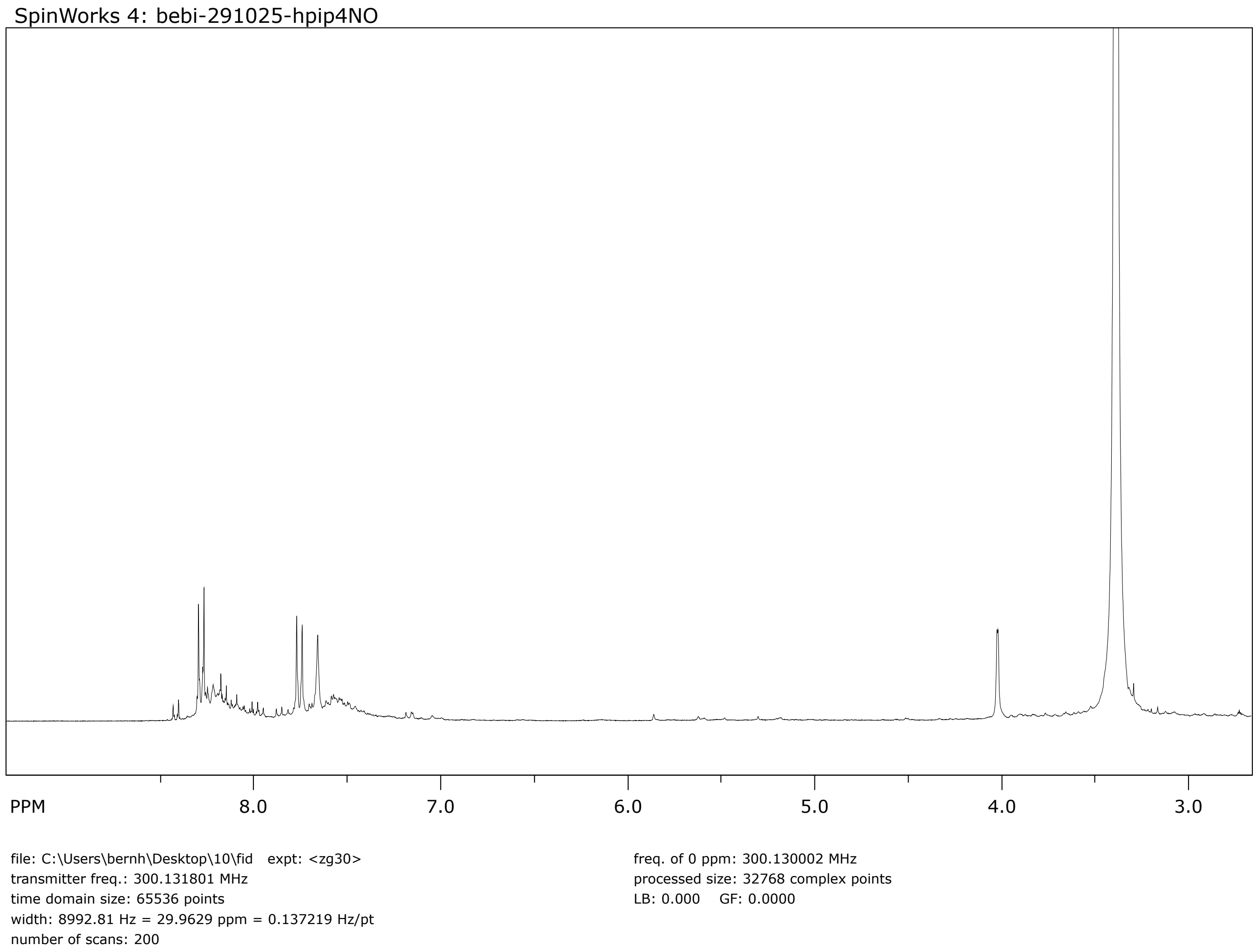

Figure S49. ^{1}H NMR spectrum of HPip-4NO.

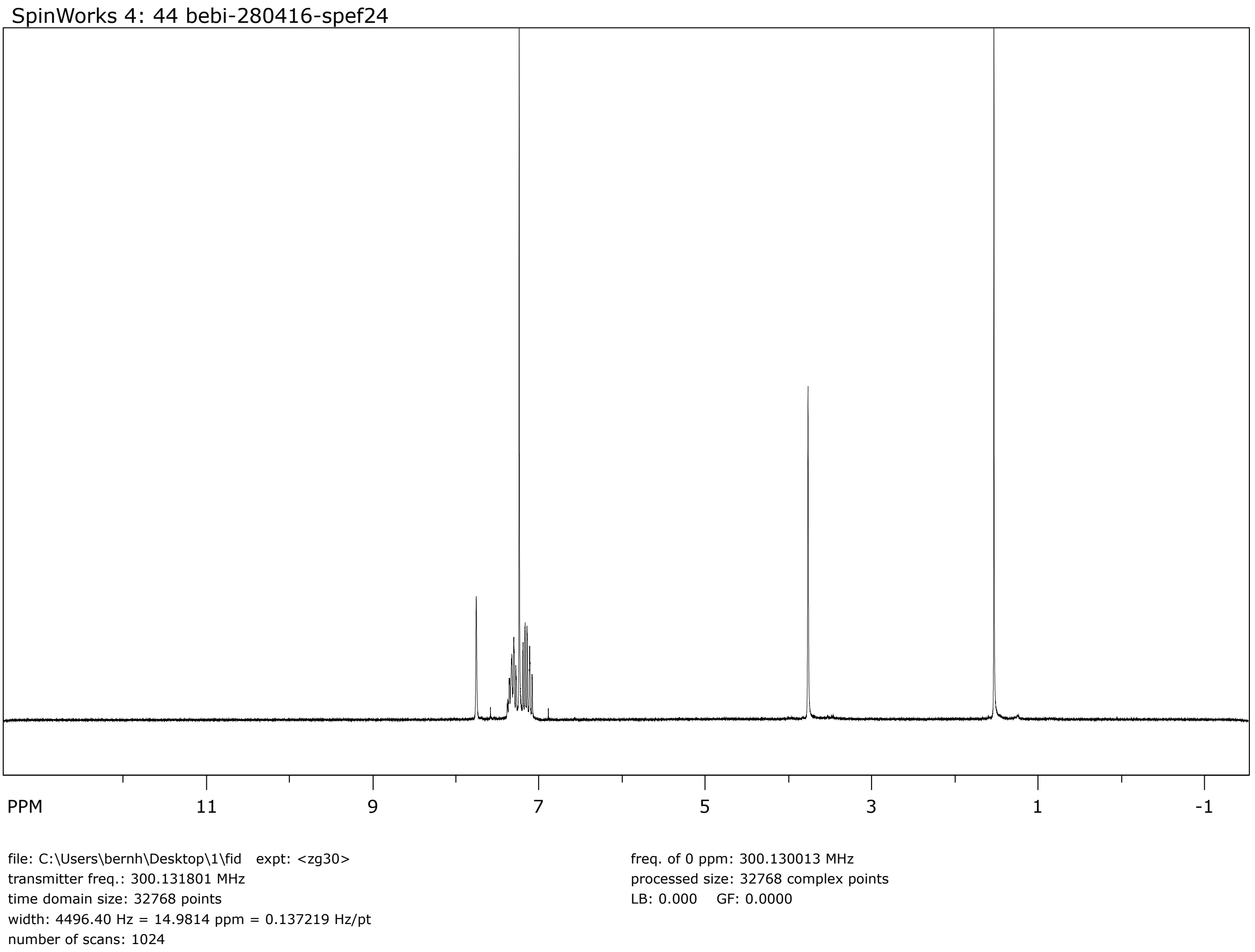

Figure S50. ^{1}H NMR spectrum of Spyr-EF24.

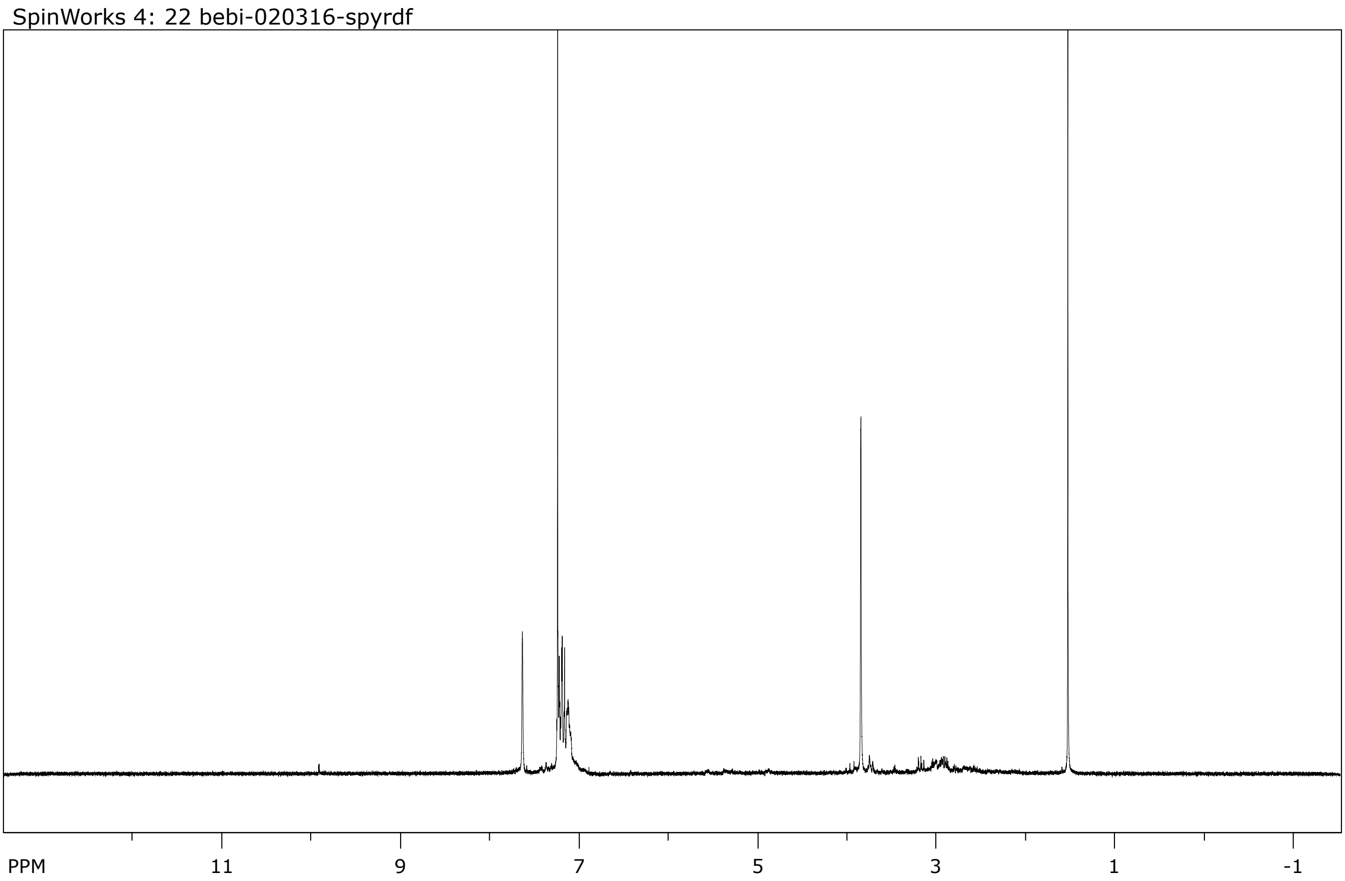

file: C:\Users\bernh\Desktop\1\fid   expt: <zg30>
transmitter freq.: 300.131801 MHz
time domain size: 32768 points
width: 4496.40 Hz = 14.9814 ppm = 0.137219 Hz/pt
number of scans: 100

freq. of 0 ppm: 300.130012 MHz
processed size: 32768 complex points
LB: 0.000   GF: 0.0000

Figure S51. ^{1}H NMR spectrum of Spyr-DF.

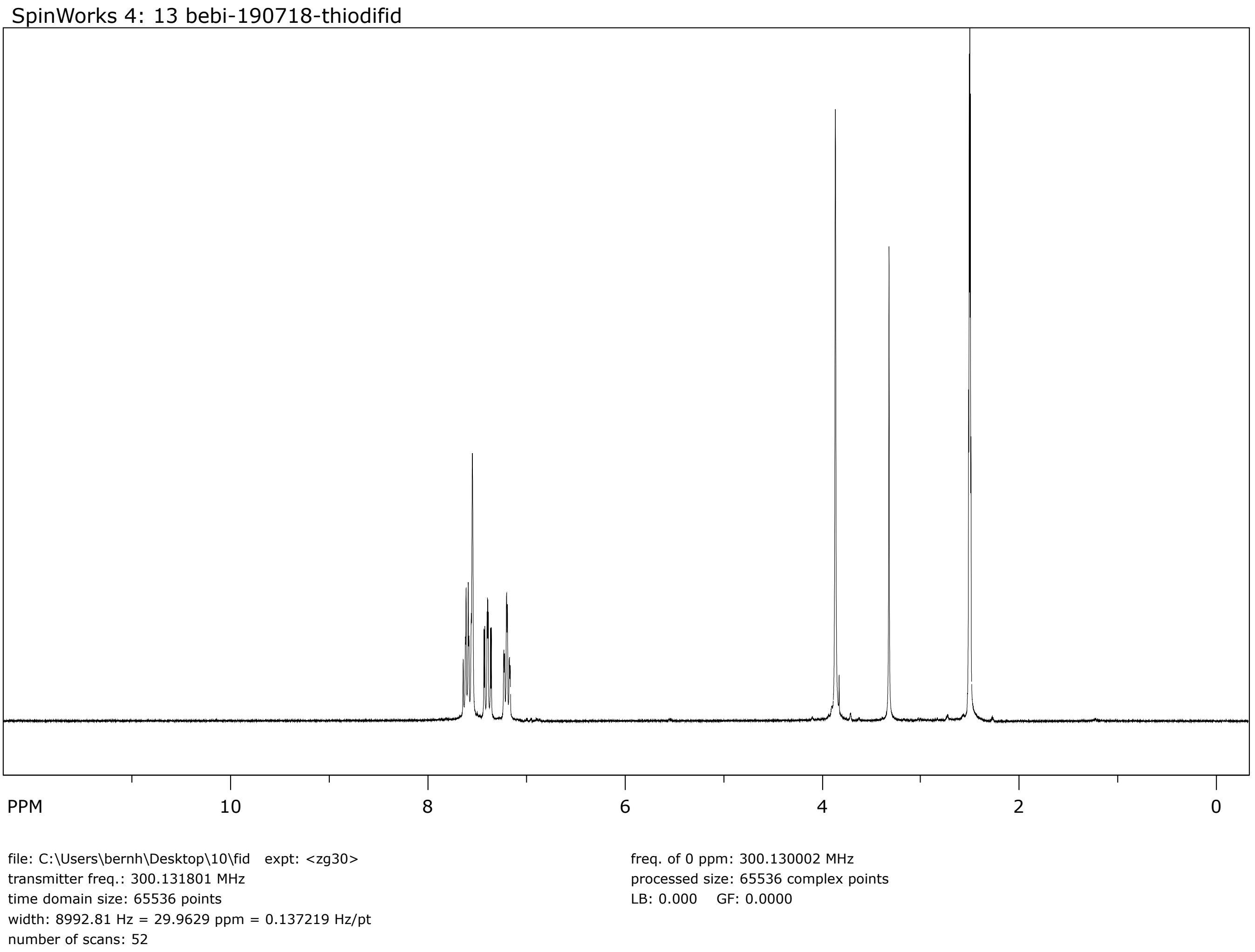

Figure S52. ^{1}H NMR spectrum of Thio-DiFiD.

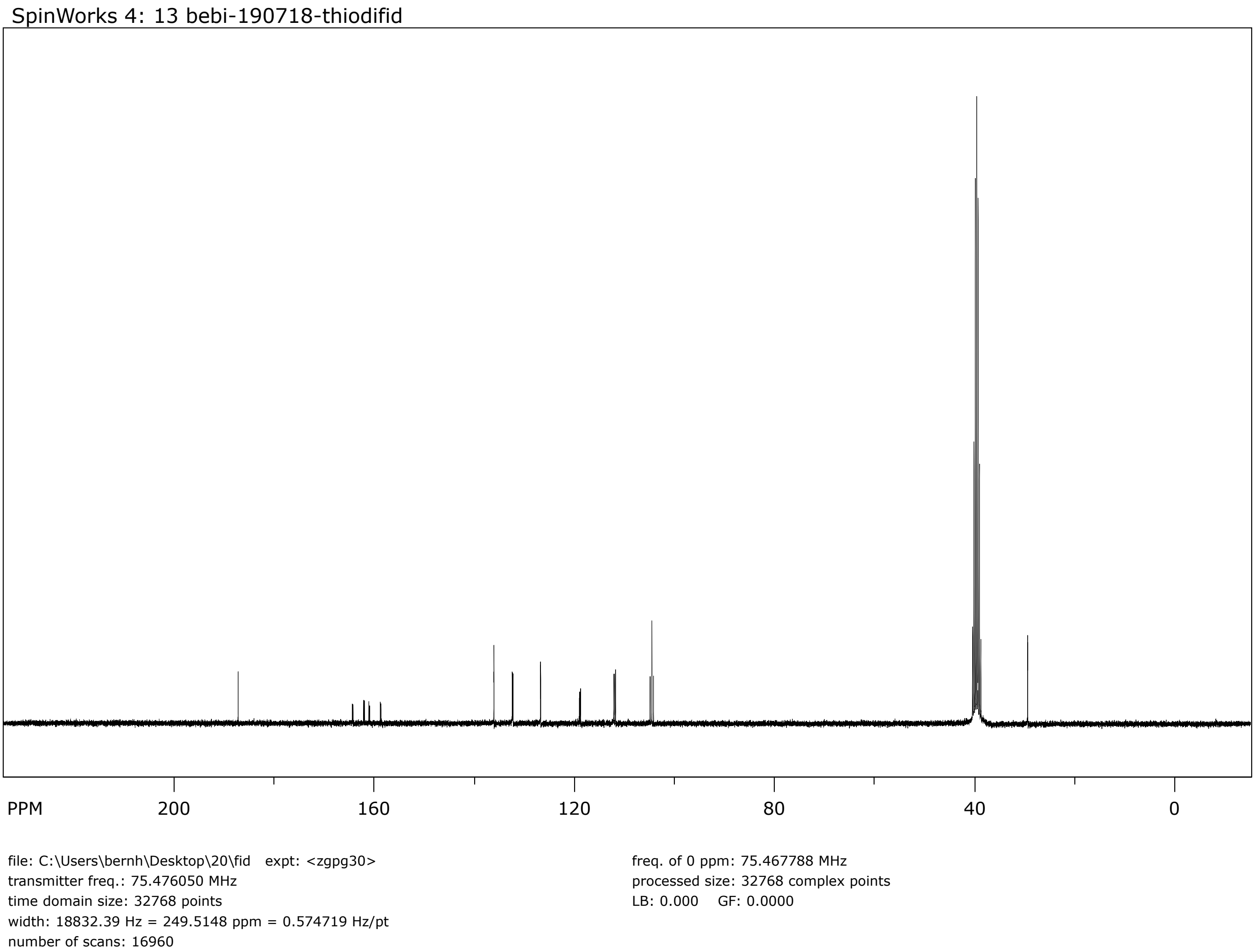

Figure S53. ^{13}C NMR spectrum of Thio-DiFiD.

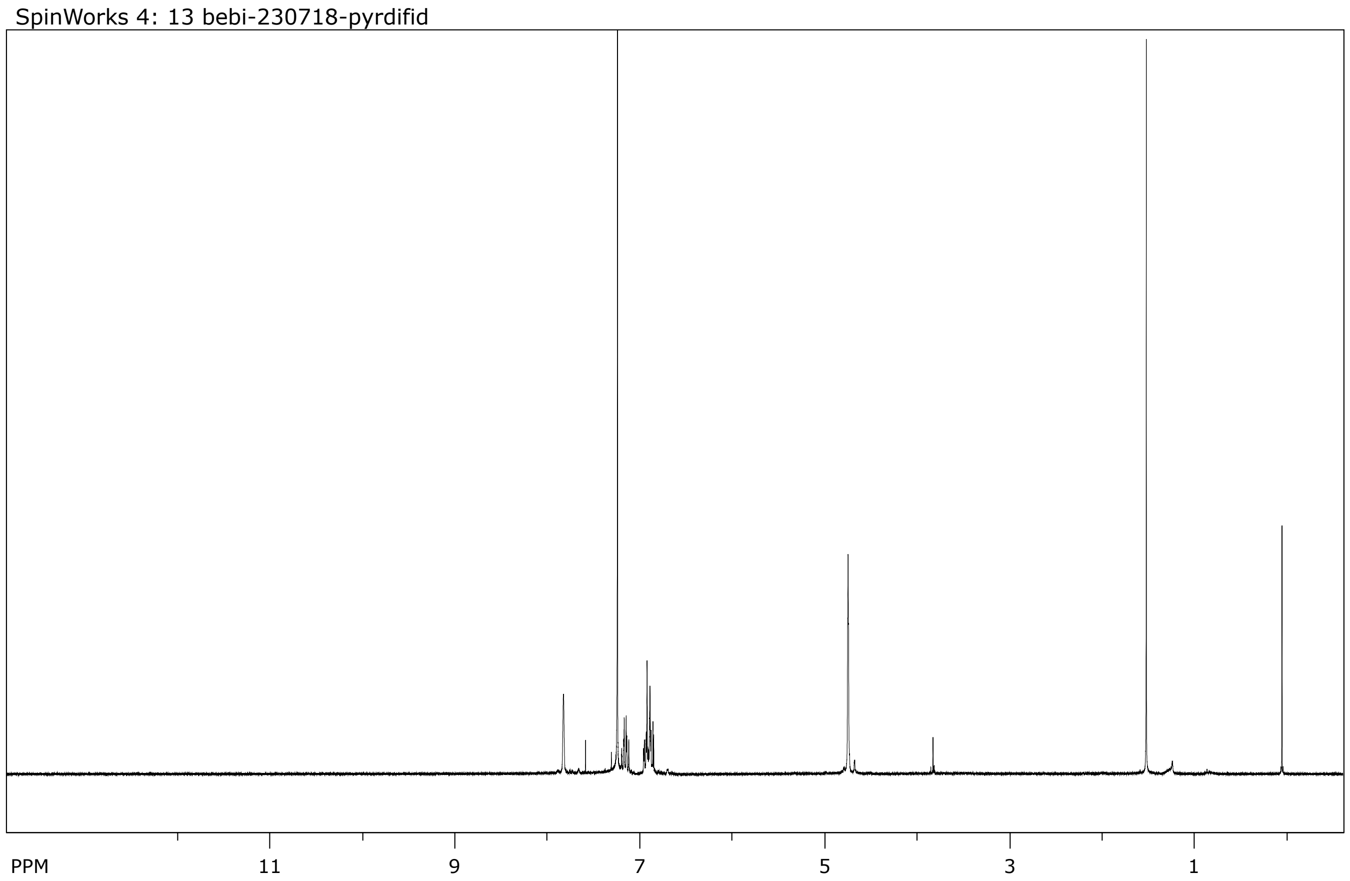

file: C:\Users\bernh\Desktop\1\fid expt: <zg30>
transmitter freq.: 300.132000 MHz
time domain size: 65536 points
width: 5787.04 Hz = 19.2816 ppm = 0.088303 Hz/pt
number of scans: 300

freq. of 0 ppm: 300.130013 MHz
processed size: 65536 complex points
LB: 0.000 GF: 0.0000

Figure S54. ^{1}H NMR spectrum of Pyrano-DiFiD.

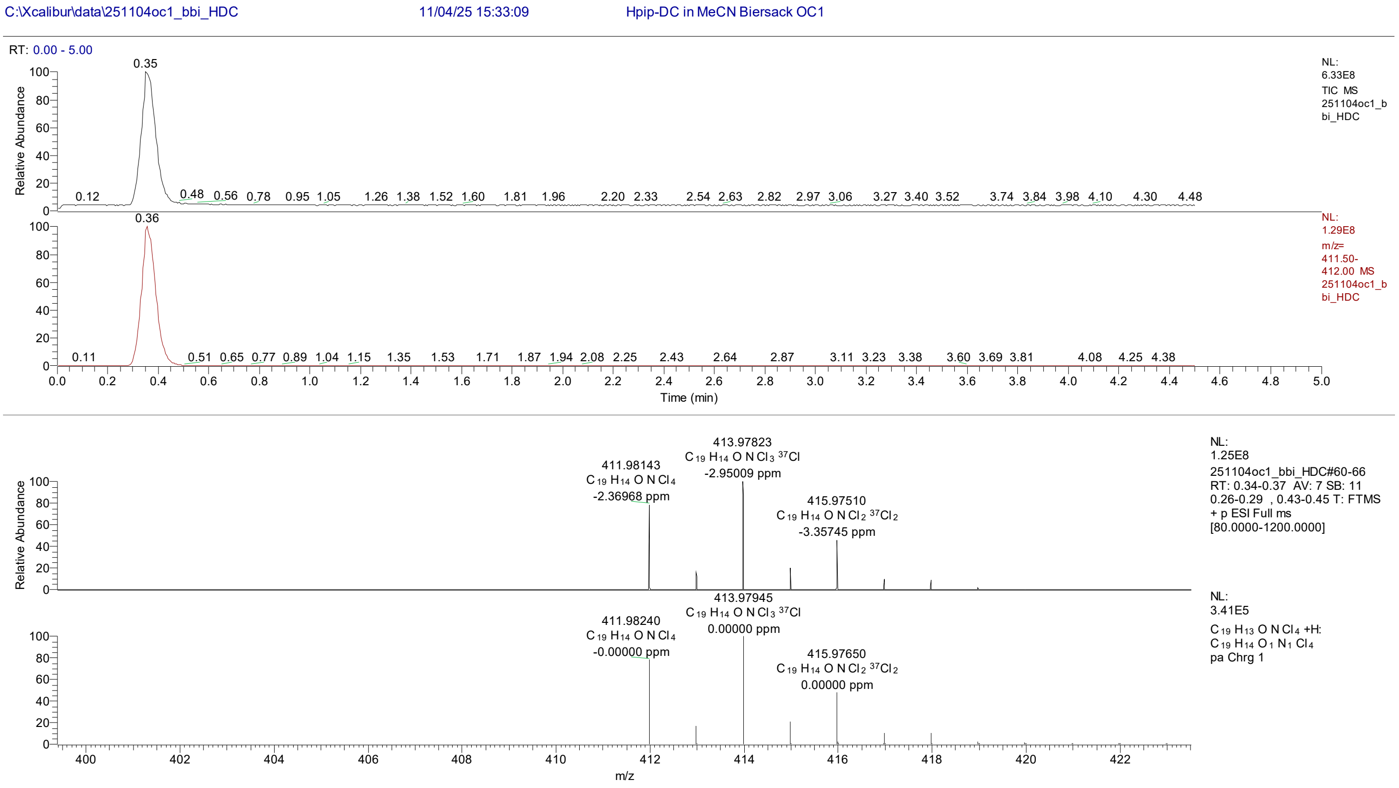

Figure S55. HR-MS spectrum (ESI) of HPip-DC.

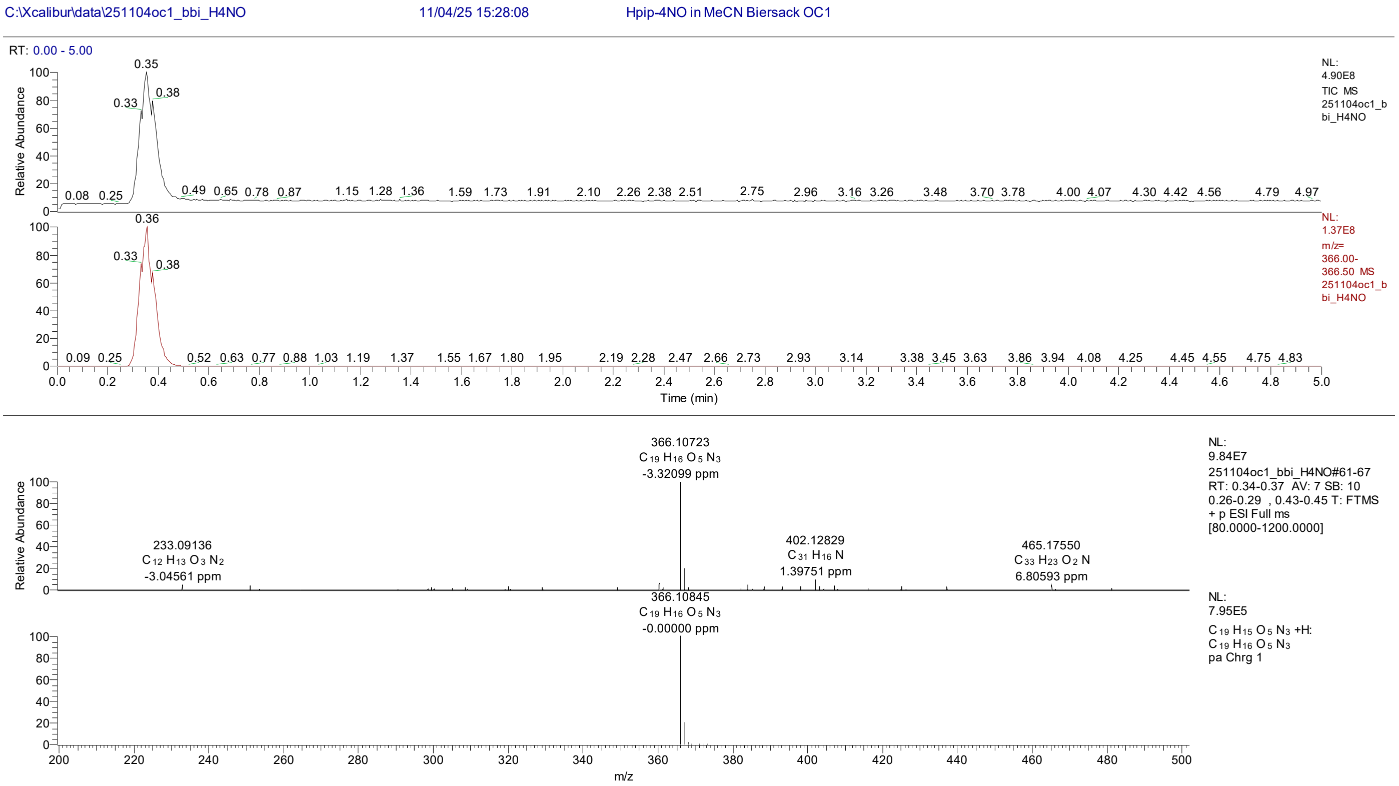

Figure S56. HR-MS spectrum (ESI) of HPip-4NO.
